# Subtelomeric assembly of a multi-gene pathway for antimicrobial defense compounds in cereals

**DOI:** 10.1038/s41467-021-22920-8

**Published:** 2021-05-07

**Authors:** Yan Li, Aymeric Leveau, Qiang Zhao, Qi Feng, Hengyun Lu, Jiashun Miao, Zheyong Xue, Azahara C. Martin, Eva Wegel, Jing Wang, Anastasia Orme, Maria-Dolores Rey, Miroslava Karafiátová, Jan Vrána, Burkhard Steuernagel, Ryan Joynson, Charlotte Owen, James Reed, Thomas Louveau, Michael J. Stephenson, Lei Zhang, Xuehui Huang, Tao Huang, Danling Fan, Congcong Zhou, Qilin Tian, Wenjun Li, Yiqi Lu, Jiaying Chen, Yan Zhao, Ying Lu, Chuanrang Zhu, Zhenhua Liu, Guy Polturak, Rebecca Casson, Lionel Hill, Graham Moore, Rachel Melton, Neil Hall, Brande B. H. Wulff, Jaroslav Doležel, Tim Langdon, Bin Han, Anne Osbourn

**Affiliations:** 1 National Centre for Gene Research, CAS-JIC Centre of Excellence for Plant and Microbial Science (CEPAMS), Centre of Excellence for Molecular Plant Sciences, Shanghai Institute of Plant Physiology and Ecology, Shanghai Institutes for Biological Sciences, Chinese Academy of Sciences (CAS), Shanghai, China; 2 John Innes Centre, Norwich Research Park, Norwich, UK; 3 Institute of Experimental Botany of the Czech Academy of Sciences, Centre of the Region Haná for Biotechnological and Agricultural Research, Olomouc, Czech Republic; 4 Earlham Institute, Norwich Research Park, Norwich, UK; 5 Joint Center for Single Cell Biology, School of Agriculture and Biology, Shanghai Jiao Tong University, Shanghai, China; 6 Institute of Biological, Environmental and Rural Sciences, Aberystwyth University, Gogerddan, Aberystwyth, Ceredigion SY23 3EE UK

**Keywords:** Agricultural genetics, Evolutionary genetics, Comparative genomics, Secondary metabolism

## Abstract

Non-random gene organization in eukaryotes plays a significant role in genome evolution. Here, we investigate the origin of a biosynthetic gene cluster for production of defence compounds in oat—the avenacin cluster. We elucidate the structure and organisation of this 12-gene cluster, characterise the last two missing pathway steps, and reconstitute the entire pathway in tobacco by transient expression. We show that the cluster has formed de novo since the divergence of oats in a subtelomeric region of the genome that lacks homology with other grasses, and that gene order is approximately colinear with the biosynthetic pathway. We speculate that the positioning of the late pathway genes furthest away from the telomere may mitigate against a ‘self-poisoning’ scenario in which toxic intermediates accumulate as a result of telomeric gene deletions. Our investigations reveal a striking example of adaptive evolution underpinned by remarkable genome plasticity.

## Abstract (web summary)

The genomic organization and origin of the avenacin biosynthetic gene cluster remain unknown. Here, the authors assemble the genome of diploid oat *Avena strigosa*, reveal the structure and organization of the consecutive genes, characterize the last two missing pathway steps, and investigate the origin of the pathway in cereals.

## Introduction

Oat belongs to the *Aveneae* tribe, which diverged from the *Triticeae* (the tribe containing the best-characterised temperate cereals, wheat and barley) around 30 million years ago, and from the *Panicoideae* (maize and sorghum) around 50–60 million years ago. A distinctive feature of oat (*Avena* species) is the ability to produce antifungal specialised metabolites (avenacins) that are synthesised in the roots and provide protection against soil-borne diseases such as take-all, a major cause of yield loss in wheat^1,2^. Previously we isolated ~100 avenacin-deficient mutants of diploid oat (*Avena strigosa* accession S75) following sodium azide mutagenesis^2^. Interestingly, genetic analysis indicated that the loci that we had defined by mutation were clustered^3^. We have subsequently cloned and characterized ten avenacin pathway genes, five of which are located on a ~300 kb BAC contig^4–6^. The remaining five^7–10^ are genetically linked to this contig^2,11^ but it is not known how physically close they are or what the full extent of clustering is. A better understanding of the organisation and evolution of the avenacin cluster would provide important insights into the origins of metabolic diversity in grasses and may also open up opportunities for engineering other cereals for enhanced disease resistance.

In addition to the avenacin cluster, biosynthetic gene clusters for a wide variety of natural products including compounds of agronomic and pharmaceutical importance have now been reported from diverse plant species^12^. A particularly intriguing question concerns the mechanisms by which these clusters of non-homologous yet functionally related genes arise, presumably in response to a particular set of selective pressures. Understanding how these clusters form and what the significance of clustering is will be crucial in understanding the relationship between genome organisation and the evolution of complex adaptive traits in eukaryotes.

Here, we employ a genomics-driven approach to investigate the nature and origin of the avenacin cluster in diploid oat and show that this 12-gene cluster has formed de novo in a subtelomeric region of chromosome 1 that lacks homology with other grasses. Intriguingly, the gene order of the cluster approximates the order of the biosynthetic steps in the pathway (a phenomenon known as ‘colinearity’ in antibiotic-producing bacteria), with the early pathway genes located nearest the telomere. Since mutations in late (but not early) avenacin pathway steps lead to accumulation of toxic intermediates^9,11^, this may mitigate against ‘self-poisoning’ as a consequence of telomeric deletion events. Our study sheds light on the mechanisms shaping genome architecture and adaptive evolution in plants.

## Results

### The genome sequence of *Avena strigosa*

**Fig. 1:**
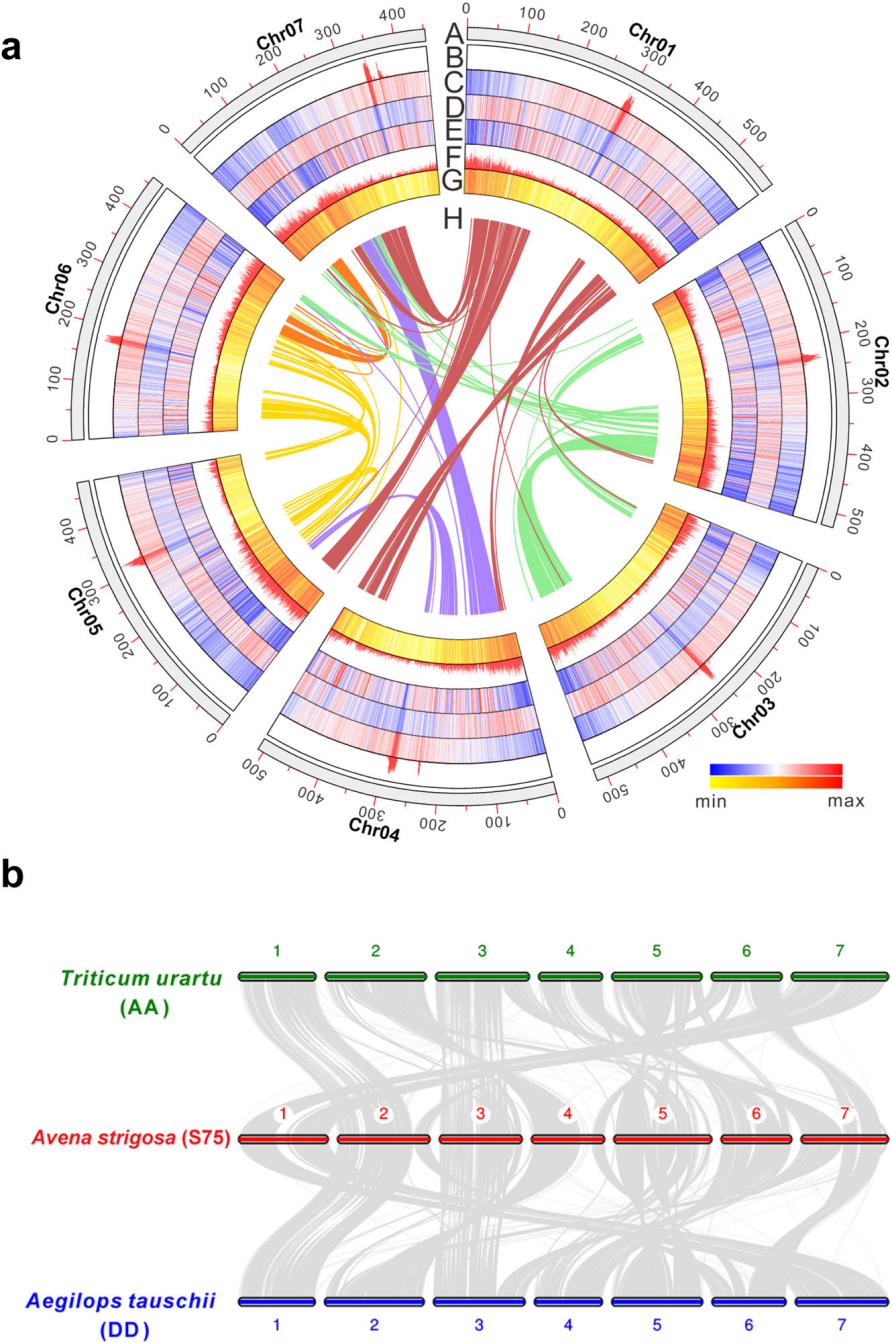
*Avena strigosa* genome features. **a** Characteristics of the seven chromosomes of *Avena strigosa*. The chromosomes are shown in track A (100 Mb intervals indicated). Tracks B–F show the densities of the long terminal repeat (LTR) retrotransposons *Cereba* (B), *Gypsy* (C), *Copia* (D), unclassified LTR retrotransposons (E), and miniature inverted-repeat transposable elements (MITEs) (F) (densities shown as percent nucleotides per 500 kb). Track G indicates the frequency of high-confidence genes (number of genes per 500 kb) and H shows syntenic blocks (1722 gene pairs, 160 blocks). **b** Synteny with *Triticum urartu* (diploid, AA) and *Aegilops tauschii* (diploid, DD).

To elucidate the complete avenacin cluster and investigate its origin we first sequenced the genome of *A. strigosa* accession S75 (2*n* = 14), the accession used in our original forward mutant screen for identification of avenacin pathway mutants^2^. The genome size was estimated as ~4.1 Gb/1C by flow cytometry (Supplementary Fig. 1) and ~3.8 Gb by *k*-mer analysis (Supplementary Fig. 2). Our flow cytometric estimate was ~6% lower than a previous report for *A. strigosa*^13^, which may reflect intraspecific variation in genome size. The genome sequence was assembled in three steps (Supplementary Fig. 3). Oxford Nanopore PromethION was applied to assemble de novo contigs using SMARTdenovo, with more than 428 Gb passed reads corrected by Canu (version 1.6). An Illumina whole-genome shotgun dataset was used to correct low-quality nucleotides and insertions/deletions (InDels) from the Nanopore sequencing by three rounds of Pilon, yielding a 3.50 Gb assembly with a contig N50 of 4.77 Mb (Supplementary Table 1). These polished contigs were then processed for hybrid assembly using Direct Label and Stain Technology (DLS) optical maps. We assembled 289 hybrid scaffolds with a total combined length of 3.53 Gb and a scaffold N50 of 73.4 Mb (Table 1). We then used Hi-C (high-throughput/resolution chromosome conformation capture) to generate a chromosome-scale assembly (Supplementary Fig. 4). The seven largest scaffolds contained 99.9% of the assembly and represent the haploid chromosomes of *A. strigosa* (Fig. 1a; Supplementary Table 2). The Illumina paired-end data were mapped with high efficiency to the assembly, with 98.4% of the reads mapping to the genome. Comparison of the assembly with the genetic linkage map for oat recently published by Latta et al.^14^ provided further evidence for the accuracy of the assembly with 5515 (~40%) of 64-base tag-level haplotypes having perfect matches to single sites on the seven chromosomes (Supplementary Fig. 5). Further details of genome sequence analysis, assembly and annotation can be found in Supplementary Information file.

**Table 1:** *A. strigosa* S75 genome statistics and gene predictions.

	Number	Size
Assembly feature		
Estimated genome size		4.1 Gb
Assembled sequences		3,530,496,476 bp
N50 length (contig)		4,770,050 bp
Longest contig		28,939,783 bp
N50 length (scaffolds)		73,363,070 bp
N80 (scaffolds)		31,630,497 bp
Number of scaffolds (>N80)	35	
Longest scaffold		272,903,458 bp
Transposable elements		
Retrotransposons		2,390,363,061 bp (67.7%)
DNA transposons		219,943,352 bp (6.2%)
Other		254,548,795 bp (7.2%)
Total		2,864,878,992 bp (81.1%)
Genome annotation		
Gene models (high confidence)	39,885	148,367,003 bp
Gene models (lower confidence)	36,816	92,666,279 bp
Noncoding RNAs	1999	197,635 bp

RNA-seq data for six different tissues of *A. strigosa* accession S75 (whole roots, root tips, leaves, panicles, shoots and spikelets) were generated previously^9^. A total of 39,885 high-confidence (HC) and 36,816 low-confidence protein-coding genes were annotated using the EvidenceModeler (EVM) annotation pipeline^15^ based on de novo prediction, homology annotation, and RNA-seq prediction (Table 1; Supplementary Fig. 6, Supplementary Table 3). Functional analysis showed that 34,928 (87.6%) of HC genes were annotated (Supplementary Table 4). The completeness of the gene space was quantified by searching for 1375 highly conserved plant-specific single-copy orthologs (database: embryophyta_odb10) using BUSCO^16^, of which 95.5% were correctly predicted (Supplementary Table 5). The LTR Assembly Index (LAI)^17^, which evaluates the assembly quality of the intergenic and repetitive sequence space, was 11.51, indicating that the 4.1 Gb genome assembly is of high quality. The total repetitive sequences accounted for 81.1% (2.87 Gb) of the assembled genome sequence (Table 1; Fig. 1a; Supplementary Table 6). Comparison of the *A. strigosa* S75 genome with *Triticum urartu*^18^ and *Aegilops tauschii*^19^, the sources of the AA and DD genomes of hexaploid wheat respectively, revealed large syntenic blocks in common with the genomes of these species (Fig. 1b).

### The 12-gene avenacin cluster

**Fig. 2:**
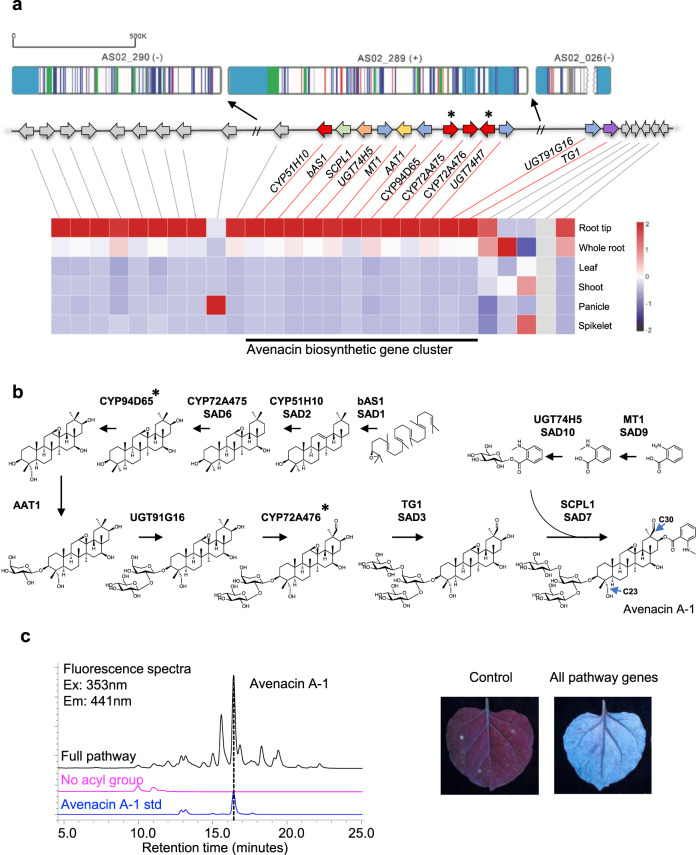
The complete 12-gene avenacin biosynthetic cluster and full pathway reconstitution by transient expression in *Nicotiana benthamiana*. **a** The region of the *A. strigosa* genome encompassing the avenacin biosynthetic gene cluster. The genes shown in colour are the nine previously characterised avenacin pathway genes^4–10,20,21^ along with two previously uncharacterised *CYP* genes (asterisked), shown in this work to catalyse the two missing pathway steps (see Supplementary Table 7 for more information about all genes in this region). *UGT74H7*, which encodes a previously characterised sugar transferase related to *UGT74H5* but with low activity towards avenacin acyl precursors^10^, is also indicated. **b** The complete pathway for the biosynthesis of avenacin A-1, including the newly validated steps catalysed by CYP94D65 and CYP72A476 (asterisked) (Supplementary Figs. 9 and 10; Supplementary Table 8). **c** Reconstitution of the avenacin pathway in *N. benthamiana* by transient expression. Full pathway: co-expression of GoldenGate constructs EC80344 (*bAS1/Sad1* + *CYP51H10/Sad2* + *CYP72A475/Sad6* + *CYP94D65* + *CYP72A476*), EC80345 (*AAT1* + *UGT91G16* + *TG1* + *P19*), EC80379 (*MT1/Sad9* + *UGT74H5/Sad10* + *SCPL1/Sad7*). No acyl group control: co-expression of EC80344 and EC80345 only. Leaves were harvested 5 days after agro-infiltration, freeze-dried, and extracts analysed by high-performance liquid chromatography. Avenacin A-1 has strong autofluorescence under ultra-violet illumination. A peak with the same retention time as the avenacin A-1 standard was detected in extracts from leaves co-expressing all of the pathway genes but not in extracts from no acyl group control leaves. Mass spectra in both positive and negative modes confirmed that this peak had the same mass as avenacin A-1 (Supplementary Fig 11). Source data underlying Fig. 2a are provided as a Source Data file.

Previously we showed that five of the genes required for avenacin biosynthesis (*bAS1/Sad1, CYP51H10/Sad2, SCPL1/Sad7, MT1*, and *UGT74H5*) are contiguous on a ~300 kb BAC contig^4,6^. Although the five other characterized pathway genes are known to be genetically linked to this cluster of genes, their physical locations in the genome (and their positions relative to each other) were not known^7–11^. Our investigation of the sequenced *A. strigosa* S75 genome revealed that the five-gene contig containing *bAS1/Sad1, CYP51H10/Sad2, SCPL1/Sad7, MT1*, and *UGT74H5* is located on scaffold AS02_289 (1.3 Mb) along with three other characterized pathway genes (*AAT1, CYP72A475/Sad6*, and *UGT74H5*) (Fig. 2a). These genes are all expressed preferentially in the root tips, the site of avenacin biosynthesis, as previously shown^4–10,20^. Two additional genes predicted to encode cytochrome P450 (CYP) enzymes of unknown function (*CYP94D65* and *CYP72A476*) are also located on this scaffold and are co-expressed with the other avenacin pathway genes (marked with asterisks in Fig. 2a). Only two other genes were located on scaffold AS02_289—*UGT74H7*, which encodes a previously characterised sugar transferase related to *UGT74H5* but with low activity towards avenacin acyl precursors^10^, and a predicted fatty acyl-CoA reductase 2-like enzyme of unknown function (Fig. 2a; Supplementary Table 7). No other intervening genes were detected in this region. AS02_289 is located very close to the end of the long arm of chromosome 1 of *A. strigosa* S75. The proximal scaffold (scaffold AS02_026, 2.6 Mb) (Fig. 2a) contains a further two recently characterized pathway genes encoding the UDP-glucose dependent sugar transferase UGT91G16 and the transglucosidase TG1/SAD3, which add the last two sugars to the avenacin trisaccharide chain^9^. These latter two genes are also root tip-expressed^9^. The scaffold distal to AS02_289 (closer to the telomere) in Fig. 2A (AS02_290, 0.9 Mb) contains a total of nine genes with various predicted functions, including in metabolism, most of which are also root-tip expressed (Fig. 2a; Supplementary Table 7). Despite the high calibre of the genome assembly as evidenced by LAI, BUSCO (Supplementary Table 5) and BAC sequence validation (Supplementary Fig. 7) it was not possible to bridge the gaps between the three scaffolds with optical mapping, most likely because of the large number of repetitive elements at the scaffold ends (Supplementary Fig. 8).

Two steps in the avenacin biosynthetic pathway remain uncharacterised: namely, the enzymes for the introduction of a hydroxyl at the C-23 position and an aldehyde group at the C-30 position (indicated on the structure of avenacin A-1 in Fig. 2b). Both of these steps are likely to be catalysed by CYP enzymes. We therefore reasoned that the two uncharacterised co-expressed *CYP* genes located on scaffold AS02_289, *CYP94D65* and *CYP72A476*, were likely candidates. We have previously used *Agrobacterium*-mediated transient expression in *Nicotiana benthamiana* to investigate the functions of other avenacin pathway genes^5–9,21^. Co-expression of CYP94D65 with bAS1/SAD1, the triterpene synthase that makes the avenacin scaffold β-amyrin (Fig. 2b) resulted in the formation of a new compound which we showed by mass spectrometry and NMR to be 23-hydroxy-β-amyrin (Supplementary Fig. 9; Supplementary Table 8). The remaining CYP, CYP72A476, was previously identified as a candidate for avenacin biosynthesis and screened for activity in *N. benthamiana* against early pathway intermediates, but no activity was detected on any of the intermediates tested^8^. More recently we have shown that oat mutants that cannot perform glycosylation at C-3 (mutated in *AAT1*/*UGT91G16*) accumulate intermediates that lack the C-30 aldehyde^7,9^, suggesting that C-30 oxidation requires prior glycosylation at the C-3 position. Co-expression of CYP72A476 with the other pathway enzymes confirmed that CYP72A476 introduces the aldehyde group at C-30 and that this step is dependent on C-3 glycosylation (Supplementary Fig. 10). We then generated expression constructs for all of the previously characterized pathway enzymes, together with *CYP94D65* and *CYP72A476*. Avenacin A-1 has strong autofluorescence under ultra-violet illumination^1,2^. Co-infiltration of *Agrobacterium tumefaciens* strains containing these constructs coupled with LC–MS resulted in production of readily detectable levels of avenacin A-1 when transiently expressed in *N. benthamiana* (Fig. 2c; Supplementary Fig. 11), indicating that the entire pathway had been delineated and successfully reconstituted.

**Fig. 3:**
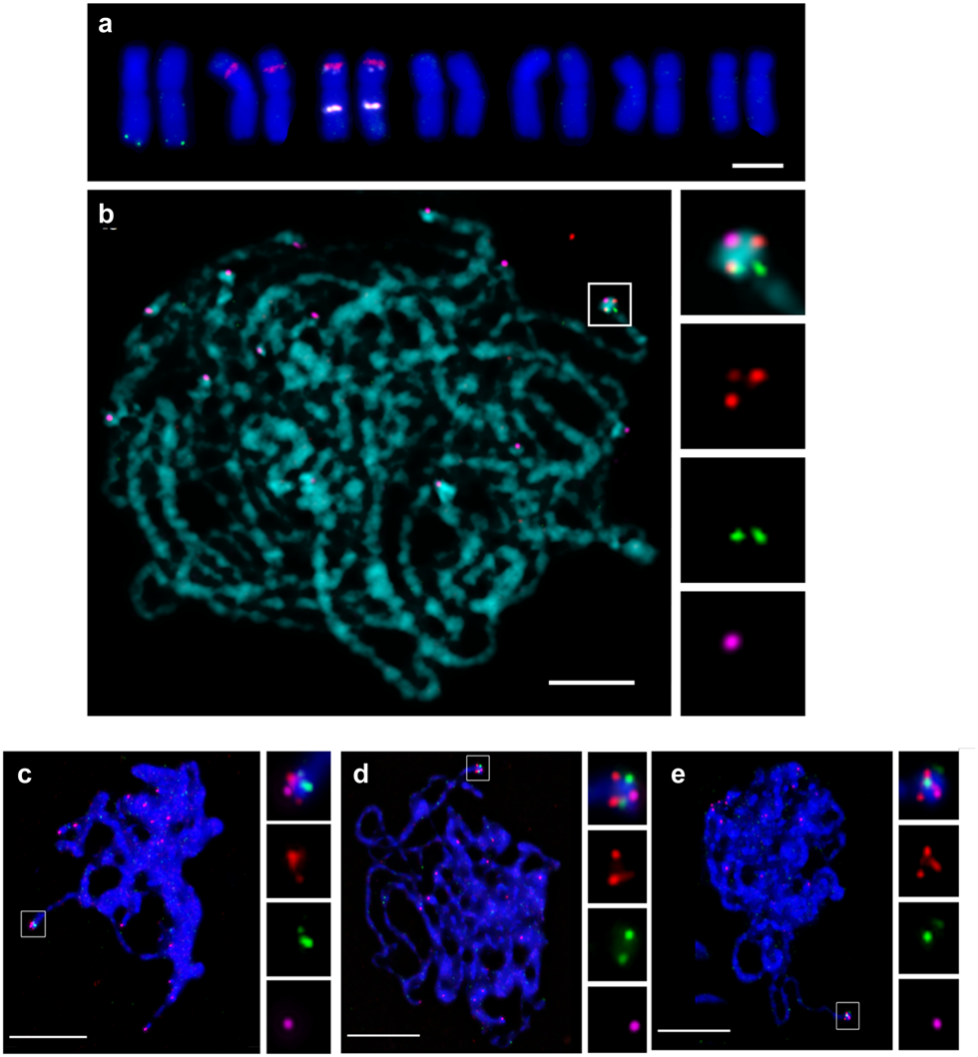
*bAS1/Sad1* and *TG1/Sad3* are co-located close to the telomere of the long arm of chromosome 1. **a** A chromosome set from a mitotic metaphase spread. Chromatin, blue; *bAS1/Sad1*, green; nucleolus organiser regions (labelled with pTa71), red; 5S rDNA loci (labelled with pTa794), white. The nucleolus organiser regions (pTa71) are localised on chromosomes 2 and 3 and the 5S rDNA loci (pTa794) on chromosome 3^98^. Chromosomes 6 and 7 are the shortest chromosomes in the genome, submetacentric in contrast to the chromosome carrying *bAS1/Sad1* and significantly shorter. A comparison of the lengths of the chromosome carrying *bAS1/Sad1* and the two other unidentified chromosomes in the genome—metacentric chromosome 4 and submetacentric chromosome 5—showed that the chromosome carrying *bAS1/Sad1* was significantly longer than the other two and is therefore chromosome 1 (*t*-test *p* values <0.01 and <0.001, respectively, *n* = 8 for each chromosome). **b** Meiotic pachytene cell: chromatin, blue; *bAS1/Sad1*, red; *TG1/Sad3*, green; telomeres, magenta. Homologous chromosomes are paired at this stage. In the enlarged views (boxed regions on the right), *bAS1/Sad1* (red) is visible as one fluorescent focus per homologous chromosome with an additional faint focus caused by bleed through coming from the telomere label (magenta). Scale bars: 5 µm. **c**–**e** FISH localisation of *bAS1/Sad1* (red) and *TG1*/*Sad3* (green) in relation to the telomere (magenta) during meiotic pachytene. *Sad1* foci appear closer to the telomere in 28% of the pachytene cells analysed (**c**); *TG1/Sad3* foci appear closer to the telomere in 7% of the cells (**d**); both foci overlap in 65% of the cells (**e**). Each pachytene FISH experiment involved 4–5 individual slides, each using a different anther. Scale bars: 10 μm. Source data are provided as a Source Data file.

We further investigated the chromosomal location and organisation of the avenacin biosynthetic gene cluster by karyotyping and DNA fluorescence in situ hybridisation (FISH). Karyotype analysis revealed that *bAS1/Sad1*, the gene encoding the first step in the avenacin pathway, is located close to the end of the long arm of chromosome 1 (Fig. 3a). DNA FISH using probes for *bAS1/Sad1*^20^ and the genetically linked locus *TG1/Sad3*^2,9,11^, which are on distinct scaffolds, revealed that these two genes are in close physical proximity and co-localise to this region (Fig. 3b). The DNA FISH results therefore support the adjacency of these two scaffolds (as suggested above and in Fig. 2a). Due to the proximity of *bAS1/Sad1* and *TG1/Sad3,* it was not possible to establish their relative positions in relation to the telomere on metaphase chromosomes. Meiotic pachytene chromosomes are on average 10–40 times longer than metaphase chromosomes from the same species, providing much higher resolution for FISH mapping^22,23^. Even so, the resolution of pachytene chromosome spreads was still not enough to give a definitive position for *bAS1/Sad1* and *TG1/Sad3* in relation to the telomere. In most cases (around 65% of cells) *bAS1/Sad1*, *TG1/Sad3* and the telomeres partially overlapped. However, *bAS1Sad1* foci appeared closer to the telomere in ~28% of the pachytene cells analysed, while *TG1/Sad3* foci appeared closer to the telomere in around 7% of the cells (Fig. 3c–e). Flow cytometric sorting of chromosome 1 in combination with DNA FISH confirmed that the avenacin gene cluster is located close to the end of this chromosome (Supplementary Fig. 12).

### De novo formation of the avenacin cluster

**Fig. 4:**
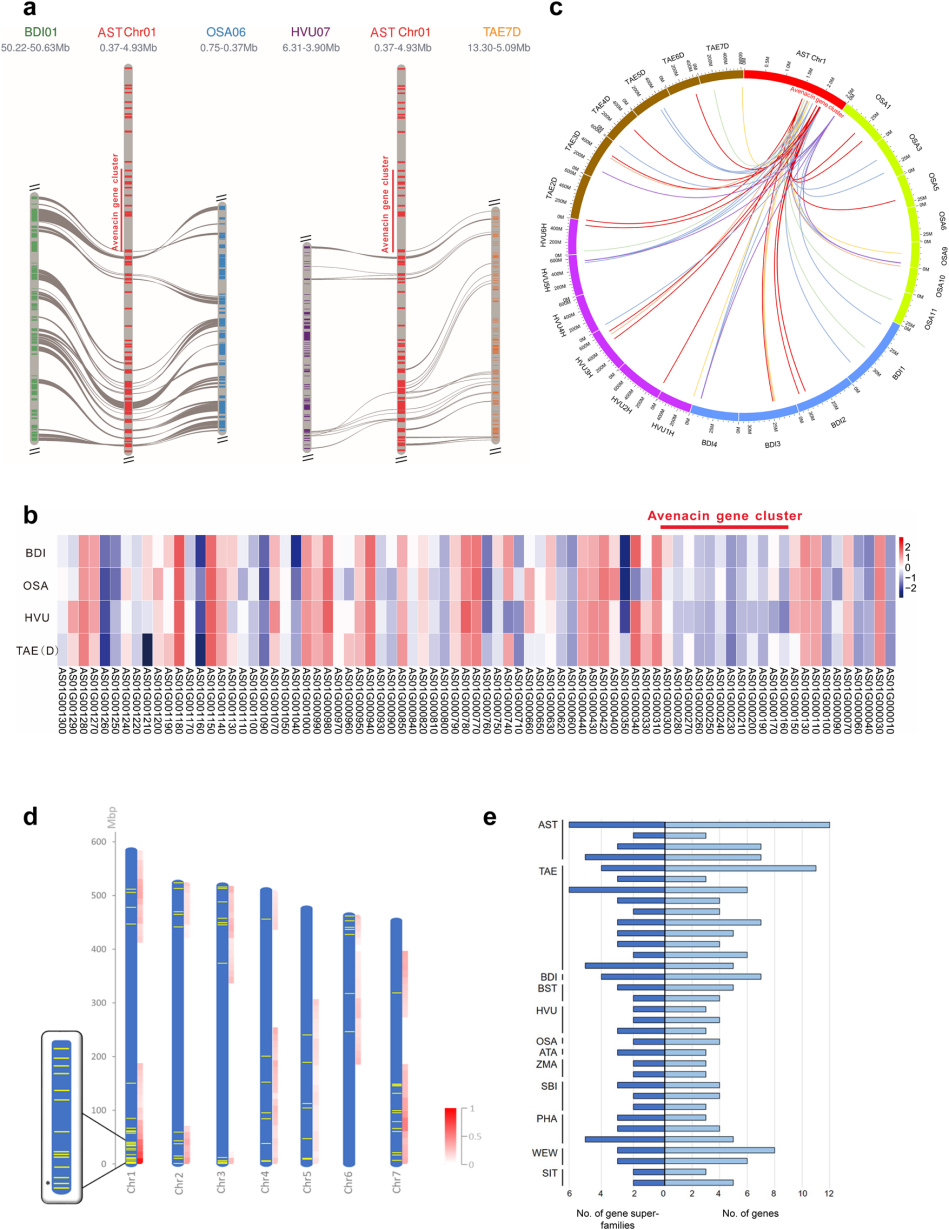
The high complexity 12-gene avenacin cluster has assembled de novo in a region of the *A. strigosa* genome that does not share synteny with other cereals. **a** Alignment of the *B. distachyon* (BDI), rice (OSA), barley (HVU) and wheat (TAE; DD genome shown) showing lack of synteny in the avenacin cluster region. **b** DNA sequence identity heatmap of the avenacin pathway genes and the other *A. strigosa* genes in the region shown in Fig. 2a  with the most closely related sequences in *B. distachyon* (BDI), rice (OSA), barley (HVU) and wheat (TAE) (Table S8). **c** Circos plot showing the locations of these closest matches on the chromosomes of *B. distachyon* (blue), rice (green), barley (purple) and wheat (DD genome) (brown). **d** Locations of plantiSMASH-predicted biosynthetic gene clusters (yellow lines) in the *A. strigosa* genome. Cluster density scores for 100 Mb-sized sliding windows are shown in red. The avenacin cluster is asterisked in the enlarged view of the terminal region of chromosome 1. **e** Schematic showing the number of genes and gene super-families per cluster in putative triterpene biosynthetic clusters predicted by plantiSMASH in the genomes of *A. strigosa* S75 (AST), wheat (TAE), *Brachypodium distachyon* (BDI), *Brachypodium stacei* (BST), barley (HVU), rice (OSA), maize (ZMA), *Sorghum bicolor* (SBI), *Panicum hallii* (PHA), wild emmer wheat (WEW), and *Setaria italica* (SIT). The avenacin cluster is shown at the top.

The pseudochromosome-level *A. strigosa* assembly and DNA FISH results both indicate that the avenacin cluster lies in a subtelomeric region at the end of the long arm of chromosome 1 and is oriented with the early pathway genes (*bAS1/Sad1*, *CYP51H10*) closest to the telomere. Estimates of the times of divergence of *A. strigosa* from other cereals and grasses based on synonymous substitutions (Ks) of orthologous gene pairs were 28.5 mya (*T. urartu*), 28.7 mya (*A. tauschii*), 31.1 mya (barley), 32.6 mya (*Brachypodium* *distachyon*), 52.9 mya (rice), 58.9 mya (sorghum) and 65.1 mya (maize) (Supplementary Fig. 13). We next analysed collinearity between *A. strigosa* and other cereals and grasses in this region. Collinearity was observed with the corresponding chromosomes of *B. distachyon*, rice, barley and wheat (DD genome shown)^24–27^ on the centromeric side flanking the avenacin cluster (Fig. 4a). However, synteny breaks down completely at the edge of the cluster. Since the genes immediately adjacent to *TG1/Sad3* have close homologues in these other species, this breakdown in synteny can be pinpointed to the region between *TG1/Sad3* and the immediate flanking gene *AS01G000310*, shown in grey in Fig. 2a.

The genes within the avenacin cluster collectively encode multiple different types of enzymes. Each of these classes of enzyme are encoded by multi-gene families in plant genomes, making comparative genomics of genes for specialized metabolism across different plant species problematic. We took all the genes within the region of oat chromosome 1 shown in Fig. 4a and searched for the most closely related sequences in *B. distachyon*, rice, barley and wheat (Supplementary Data 1). Sequence similarity analysis revealed that the avenacin cluster genes showed considerably less similarity to their corresponding top hits in these other species when compared with the genes outside the cluster (Fig. 4b), suggesting that the cluster region has undergone accelerated sequence divergence. Furthermore, these top hits are not clustered, as shown by the Circos plot in Fig. 4c. The closest matches to the genes in the region between the avenacin cluster and the telomere (Fig. 2a) were similarly not found to be clustered in *B. distachyon*, rice, barley or hexaploid wheat (Supplementary Fig. 14). Interestingly, however, in the diploid wheat species *T. urartu*^18^ and the tetraploid *Triticum turgidum ssp. dicoccoides* (wild emmer wheat)^28^ the putative homologues of the majority of genes within this region are co-located on chromosome 3 (Supplementary Fig. 15). This is not the case in the tetraploid *T. turgidum* ssp. *durum* (durum wheat)^29^ (Supplementary Fig. 15). Circos plot analysis within *A. strigosa* indicated that the closest matches to the avenacin cluster genes are distributed around the genome (Supplementary Fig. 16). Thus, the avenacin cluster appears to have assembled de novo at the end of the long arm of chromosome 1 since the divergence of oat from other cereals and grasses.

Analysis of the *A. strigosa* genome using plantiSMASH, an algorithm designed to predict biosynthetic gene clusters in plant genomes^30^, predicted a total of 83 clusters (Fig. 4d; Supplementary Table 9), including three triterpene-related clusters (i.e., clusters that include an oxidosqualene cyclase gene) in addition to the avenacin cluster. This analysis also revealed that the avenacin cluster appears to reside within a clustering ‘hotspot’; the terminal 100 Mb region of chromosome 1 contains a total of 19 putative gene clusters, of which 17 clusters include at least three co-expressed genes (*r*-val > 0.85) (Supplementary Fig. 17). To assess the significance of this cluster enrichment, we scored gene cluster density, normalised by gene density, across the *A. strigosa* genome. The cluster density score was found to be substantially higher for the chromosome 1 terminal region than any other region in the *A. strigosa* genome (Fig. 4d; Supplementary Table 9). Cluster density in this terminal region is also unusually high when compared with other cereal and grass genomes, exhibiting higher density than any other region of the genomes we analysed with plantiSMASH (Supplementary Figs. 18 and 19; Supplementary Table 9). This inter-species comparison also revealed that the avenacin cluster is a notably complex triterpene biosynthetic gene cluster, both in terms of gene number and the diversity of gene families that it contains, which were found to be higher than any other putative triterpene biosynthetic gene clusters identified in our analysis (Fig.4e, Supplementary Fig. 20).

### Comparison with other oat species

Chromosome-scale genome assemblies have also recently been reported for two other diploid oat species, *Avena atlantica* and *Avena eriantha*^31^ and hexaploid oat (*Avena sativa*—OT3098 v1, PepsiCo, https://wheat.pw.usda.gov/GG3/graingenes_downloads/oat-ot3098-pepsico)*. A. atlantica*, like *A. strigosa*, is an A_s_ genome species, while *A. eriantha* is a C-genome (C_p_) species. The genome assemblies for these two species are slightly smaller than our *A. strigosa* assembly (*A. atlantica*, 3.69 Gb; *A. eriantha* 3.78 Gb). Syntenic analysis reveals good synteny between *A. strigosa* and *A. atlantica*, and disrupted blocks of synteny and extensive rearrangements when *A. strigosa* is compared to *A. eriantha* (Supplementary Fig. 21). Comparison of the *A. strigosa* avenacin cluster region with related regions of the other diploid oat species showed that *A. atlantica* has a very similar gene cluster in the syntenic position on chromosome 1 that is also root-expressed (Supplementary Figs. 22 and 23). *A. eriantha* also has a similar region on chromosome 6 but this region is more extended and contains additional genes (Supplementary Figs. S22 and 23). Although most of these *A. eriantha* genes are expressed at moderate level in the roots, they are primarily expressed in whole seedlings and in the crowns (Supplementary Fig. 23). Related regions were also found in the genome of hexaploid (AACCDD) oat (Supplementary Fig. 24). Pairwise comparisons of the avenacin gene clusters and flanking genes between *A. strigosa* and each of the two other sequenced diploid oat species reveal that the cluster is still dynamically evolving and in general has a higher evolutionary rate in *A. eriantha*, which also possesses extensive gene duplications within the cluster (Supplementary Fig. 25).

The expression of the putative avenacin gene homologues in the aerial parts of *A. eriantha* is intriguing since avenacins have previously only been known to be produced in the roots of oat^1^. Metabolite analysis confirmed that *A. eriantha*, in contrast to *A. strigosa* and *A. atlantica*, does indeed produce avenacins in the leaves (Supplementary Fig. 26). This differential was consistent when additional A and C genome oat species were examined (Supplementary Fig. 27). Interestingly, while A-genome oats produce a different type of antifungal compounds in the leaves (steroidal glycosides known as avenacosides), C-genome oats are reported to lack these^32^. Avenacins may therefore have a broader role in protecting against foliar pathogens in C-genome oats.

## Discussion

In summary, our genomics-driven approach has shed light on the organisation and evolution of a complex biosynthetic gene cluster required for disease resistance in oat. We have identified the two remaining missing pathway steps and reconstituted the entire pathway by transient expression in *N. benthamiana*. We further show that the cluster is located in a subtelomeric region of chromosome 1 of *A. strigosa*, and that this cluster has formed since the divergence of oat from other cereals and grasses. There is compelling evidence that plant biosynthetic gene clusters have not arisen by horizontal gene transfer from microbes, but rather by recruitment and neofunctionalisation of genes from elsewhere within the genome by as yet unknown mechanisms, presumably in response to a particular set of selective pressures^12^. Subtelomeric regions of eukaryotic genomes have previously been suggested to facilitate gene recombination and transposon insertions and serve as hot beds for new gene origination^33–38^. Such regions may therefore be conducive to cluster formation. Interestingly the organisation of the avenacin cluster genes appears to be loosely co-linear with the order of the biosynthetic pathway (Fig. 2a), with the early pathway genes closest to the telomere and the late pathway genes required for avenacin glucosylation towards the other end of the cluster. Proximity to the telomere could potentially enable a gradient of sequential expression to be established across the pathway genes. Such a scenario has been demonstrated for subtelomeric clusters of chromatin-regulated genes for virulence-related surface glycoproteins in the yeast pathogen *Candida glabrata*^39^, and there is evidence to implicate chromatin remodelling in regulation of the avenacin and other plant biosynthetic gene clusters^40–42^. Of note, while mutations in most of the avenacin pathway steps have no obvious detrimental effects on plant growth^4–7,20^, mutations in *UGT91G16* and *TG1/Sad3* result in the accumulation of toxic intermediates that cause severe root stunting^9,11^. The positioning of these genes furthest away from the telomere may therefore mitigate against a scenario in which such toxins accumulate as a result of telomeric gene deletions, since the early pathway genes are more likely to be lost before the *UGT91G16* and *TG1/Sad3* genes. Thus biosynthetic gene cluster assembly may require both positive selection for the final natural product and also negative selection pressure against partial inheritance (with associated accumulation of phytotoxic pathway intermediates). Our work provides insights into genome plasticity and adaptive evolution in eukaryotes. It further opens up opportunities to engineer wheat and other cereals for resistance to take-all and other diseases.

## Methods

### Plant material

The oat accession used for genome sequencing was *A. strigosa* accession S75^2^, originally obtained from the Institute of Grasslands and Environmental Research, Aberystwyth, Wales, UK (now known as the Institute of Biological, Environmental and Rural Sciences). After sowing, plants were grown in an artificial environment cabinet (Yiheng Technical Co. Ltd, MGC-350HP) at 22 °C, 12 h light (16000 Lux)/12 h dark, at 70% relative humidity. Four weeks later, the plants were moved outside (winter in Shanghai) and grown for a further 4 weeks under natural daylight conditions.

### Genome sequencing and assembly

Genomic DNA for Illumina mate-pair sequencing was extracted from leaves of 8-week-old plants using the DNeasy Plant Mini kit (Qiagen). An amplification-free approach was used to prepare sequencing libraries with insert sizes of 400–500 and 650–750 bp for paired-end reads, following a modified version of the manufacturer’s protocol (Illumina)^43^. An integrated protocol from the Mate-Pair Library v2 Sample Preparation Guide (Illumina) and the Paired-End Library Preparation Method Manual (Roche) was used to prepare mate-pair libraries with insert sizes of 3, 8 and 13 kb. The paired-end libraries were loaded into eight lanes of an Illumina HiSeq2500 system and raw data generated with read lengths of 2 × 150 bp. The mate-pair libraries with different insert sizes were loaded into 10, 6, and 6 lanes, respectively, of an Illumina HiSeq2000 system and all data were generated with read length of 2 × 76 bp.

For Oxford Nanopore PromethION library construction and sequencing, genomic DNA was extracted from leaves of 3-week-old *A. strigosa* seedlings using the QIAGEN^®^ Genomic DNA Extraction Kit (Cat #13323, Qiagen) according to the standard protocol provided by the manufacturer. Initial DNA quantification was carried out using a NanoDrop^TM^ 1000 One UV–Vis spectrophotometer (Thermo Fisher Scientific, USA) and Qubit^®^ 3.0 Fluorometer (Invitrogen, USA). DNA purity was confirmed (OD 260/280, 1.8–2.0; OD 260/230, 2.0–2.2) and fragments in the range of 10–50 kb recovered using a BluePippin automatic nucleic acid recovery instrument (Sage Science, USA). 3′ and 5′ overhangs were converted into blunt ends with NEBNext FFPE Repair Mix (NEB, Cat #M6630) and then ‘A’ base was added to the 3′ blunt ends using the A-Tailing reaction (NEBNext End repair/dA-tailing Module, NEB, Cat· #E7546). The purified A-tailed DNA was then ligated with adaptors from the Ligation Sequencing Kit (SQK-LSK109, Oxford Nanopore Technologies) using the NEBNext Quick Ligation Module (NEB, Cat· #E6056). Ligation products were then purified and used as the constructed sequencing library. The DNA library was accurately quantified using a Qubit^®^ 3.0 Fluorometer (Invitrogen, USA) and loaded into a GridION R9.5 × 5/PromethION (Oxford Nanopore Technologies, UK) flow cell for SMRT (single molecular real-time) sequencing. Sequencing results (fast5 files) were processed using the Guppy base caller. A total of 17.2 million passed reads (Q score ≥7) totalling 428 Gbps were generated with read length N50 33,452 bps. The sequencing metrics for the flow cells used in this study are summarised in Supplementary Table 1.

Several different software packages have been developed for assemblies of large genomes from high-noise single-molecule sequencing. These include Canu (version 1.6)^44^, wtdbg2 (https://github.com/ruanjue/wtdbg2) and SMARTdenovo^45^. In the assembly of the genome of the tomato species *Solanum pennellii* it was reported that Canu required far more CPU hours than SMARTdenovo, and that a combination of Canu for correction and SMARTdenovo for assembly generated the best results^46^. In addition, wtdbg2 is able to assemble at a speed ten times faster than SMARTdenovo. We therefore sent the pass reads into Canu for read correction and made the de novo assembly using wtdbg and SMARTdenovo. We tested several parameters for wtdbg and SMARTdenovo and finally found that using Canu correction reads to SMARTdenovo with the assembler parameters ‘-c 1’ and ‘-k 19’ gave good results, yielding an assembly consisting of 1384 contigs (contig N50 4,680,507 bps).

Following assembly, the ONT contigs were polished three times with Pilon (version 1.22)^47^ using an Illumina *A. strigosa* whole-genome shotgun dataset produced from two paired-end libraries with average fragment sizes of ~400 and ~750 bps, respectively. This procedure increased the N50 size of the assembly slightly to 4.77 Mb (Supplementary Table 1).

For DLS Technology optical map construction and hybrid assembly, based on the results of preliminary simulated digestion, DLE-1 was selected as the endonuclease of choice. DNA extraction was performed following the Bionano PrepTM Plant DNA Isolation Kit protocol (Bionano Genomics). Leaves of 3-week-old *A. strigosa* seedlings were fixed in formaldehyde and crushed using a homogeniser. The nuclei were harvested by density gradient centrifugation and embedded in agarose. After proteinase K digestion, the purified genomic DNA was diluted to an appropriate concentration and mixed with the pre-formulated Label Master Mix, following the Bionano PrepTM DLS protocol. After staining and quantification, the labelled DNA samples were loaded into an IrysChip nanochannel array and imaged on an Irys imaging instrument according to the Saphyr System User Guide (https://bionanogenomics.com/support-page/saphyr-system/).

After filtering out molecules that were <150 kb in length, a total of 331.5 Gb of single-molecule data was produced with an N50 length of 240.375 kb. Pairwise aligned, high-quality labelled molecules were clustered and assembled into contigs according to the BioNano Genomics assembly pipeline using the Bionano Genomics CMAP file, which provides location information for label sites within a genome map or an in silico digestion of a reference or sequence data (Supplementary Fig. 3)^48,49^. 98.1% (526137/535867) of optical map label sites were aligned to the sequence assembly. 99.29% (3,482,662 kb/3,507,464 kb) of the sequences were covered by the optical maps. All of the optical contigs can be aligned to the sequence scaffolds. Only a small portion of segments within the optical contigs (with a total length of 75,361 kb) did not find any alignments with the sequence scaffolds. The final de novo assembly generated 268 maps with a total combined length of 3360.755 Mb. The resulting map N50 was 39.457 Mb, with the largest map being 208.606 Mb. For creating hybrid scaffolds, optical maps were aligned to assembled Nanopore contigs and scaffolded using BioNano’s hybrid-scaffold tool^50^. The final hybrid assembly yielded 289 maps with a total combined length of 3530.496 Mb. The resulting map N50 was 73.363 Mb, the largest map being 272.903 Mb (Supplementary Table 1).

For Hi-C sequencing, leaves of 3-week-old seedlings were fixed in 2% formaldehyde solution. The nuclei/chromatin was extracted from the fixed tissue and digested with DpnII (New England Biolabs). The Hi-C library was constructed following the method of Wang et al.^51^. Hi-C libraries were sequenced at the National Center for Gene Research (Shanghai, China) using the Illumina 4000 platform (Illumina) to obtain 150 bp paired-end reads. Raw reads were processed by trimming adaptor and low-quality sequences using Fastp software (version 0.12.6)^52^ with default parameters. A total of 4772 Mb clean reads was kept for the mapping process. The quantity of informative Hi-C reads was estimated by HiC-pro (version 2.10.0)^53^. The de-duplicated list of alignments of Hi-C reads to our draft assembly was generated using the Juicer pipeline (version 1.5.7)^54^. Nine base pair-delimited resolutions (2.5, 1 Mb, 500, 250, 100, 50, 25, 10, 5 kb) were used to bin the reads and describe the interaction intensity of chromosome conformation. We used 3d-dna^55^ (version 180922) in haploid mode to assemble our draft assembly into chromosome-length scaffolds with the help of linking information. Only these scaffolds >15 kb were taken into the process of cluster, order and orientation. The iterative round for mis-correction was set as zero time. Hi-C contact maps were processed using the 3d-dna visualise module and reviewed in JuiceBox (version 1.9.0) (https://github.com/aidenlab/Juicebox) (Supplementary Fig. 4).

### Estimation of genome size

Nuclear DNA amounts for flow cytometry were determined according to Doležel et al.^56^. Approximately 20 mg of *A. strigosa* S75 leaf tissue and 10 mg of maize leaf tissue (*Zea mays* cv. CE-777; 2C = 5.43 pg DNA; ref. ^57^), which served as internal reference standard, were chopped with a sharp razor blade in 500 µl Otto I buffer solution (0.1 M citric acid, 0.5% v/v Tween 20)^58^. The homogenate was filtered through a 40 µm nylon mesh (BD Falcon^TM^, Cat. #352340). Nuclei were then pelleted by centrifugation (500 g, 3 min) and resuspended in 400 µl of Otto I buffer. After 30 min incubation at room temperature, 800 µl of Otto II solution (0.4 M Na_2_HPO_4_)^58^ supplemented with 50 µg/ml RNase and 50 µg/ml propidium iodide was added. Samples were analysed using a CyFlow Space flow cytometer (Sysmex Partec GmbH, Görlitz, Germany) equipped with 533 nm laser. At least 5000 nuclei were analysed per sample (Supplementary Fig. 1). Five plants were measured, and each plant was analysed three times on three different days. The 2C DNA content of *A. strigosa* S75 was calculated using the ratio of the G1 peak means and the standard, giving a 2C value for *A. strigosa* S75 of 8.486 ± 0.074 pg DNA (mean ± SD). The 1C genome size in base pairs was calculated using the formula 1 pg DNA = 0.978 × 10^9^ bp^59^, giving a 1C *A. strigosa* S75 genome size of 4149 ± 0.036 Mb (mean ± SD).

In DNA sequences, the term k-mer refers to all possible substrings of length *k* that are contained in a sequenced read. Values of k-mers were plotted against the frequency at their occurrency (Supplementary Fig. 2). The k-mer analysis method used for moso bamboo^60^ by Phusion2 (http://www.sanger.ac.uk/science/tools/phusion2) was employed to estimate the genome size of *A. strigosa* S75. At a k-mer size of 55, the peak occurrency was at 29. As per the definition of genome size, the total number of effective k-mer words divided by the k-mer depth or the k-mer occurrence number at the peak k-mer frequency Dp, Gs = (Kn-Ks). Here Kn is the total number of k-mer words and Ks is the number of single or unique k-mer words. The genome size was therefore expected to be (124,199,810,495–12,260, 521,110)/29 = 3.86 Gb, which was close to the result obtained by flow cytometry. An alternative estimation using Kmerfreq_AR (SOAPec_v2.01 package https://sourceforge.net/projects/soapdenovo2/files/ErrorCorrection/SOAPec_v2.01.tar.gz/download) gave a predicted genome size of ~3.74 Gb at k-mer 17.

### Quality of the *A. strigosa* genome assembly

To assess the accuracy of the genome assembly, we first compared the assembled scaffolds with the sequences of seven finished bacterial artificial chromosomes (BACs). The nanopore long reads were mapped to assembled scaffolds using Minimap2^61^ with default parameters. The depth of the nanopore long reads mapped to assembled scaffolds was calculated using the programme depth in the SAMtools toolkit^62^. The seven finished BAC sequences, which were obtained by Sanger sequencing, were mapped to the assembled scaffolds by MUMmer 3.22^63^. We then retrieved the regions of scaffolds to compare with the seven BACs (Supplementary Fig. 7). A total of 255 publicly available sequences in GenBank (Supplementary Data 2) were also aligned to the scaffolds of genome using MUMmer 3.22. The Illumina paired-end data were mapped to assembled scaffolds with Bowtie 2 2.2.6^64^. The overall alignment rate was 98.37%, with 96.33% properly paired alignments. We identified 391,068 heterozygous SNPs and 19,790 short indels (10 nucleotides or less, total length 86,924 bps) in the *A. strigosa* genome. Thus, the estimated overall heterozygous rate was ~0.14 polymorphisms per kb, indicating that *A. strigosa* S75 is largely homozygous.

We also used BUSCO-3.0.2b^16^ to examine genes conserved with the Embryophyta_odb10 database in the assembly (Supplementary Table 5). Finally, we calculated the LAI using the highly accurate and sensitive programme LTR_retriever^17^. We also compared the *A. strigosa* accession S75 assembly with a genetic linkage map constructed from an F6:8 recombinant inbred population generated from a cross of the AA genome diploids *A. strigosa* (CI 3815) and *Avena wiestii* (CI 1994)^14^. The 13,873 haplotypes were mapped to the chromosomes of *A. strigosa* using the aligner SMALT (http://www.sanger.ac.uk/tool/smalt-0/). A total of 5515 64-base tag-level haplotypes out of 13,873 examined had good matches (perfect match or one base mismatch) to single sites on the seven chromosomes.

### Repeat analysis

De novo repeat prediction of the *A. strigosa* S75 genome was carried out by successively using the ab initio prediction programme RepeatModeler (http://www.repeatmasker.org/RepeatModeler.html, version1.0.5) and RepeatMasker (http://www.repeatmasker.org/, version 3.3.0). RepeatModeler was used to build the de novo repeat library from the assembled genome sequence and the consensus sequences from this were manually checked by aligning to genes from the NCBI database (nt and nr; released June 2013). Using this library of 479 consensus sequences and their classification information as a database, RepeatMasker was implemented to identify and classify homologous repeat elements in the genome of *A. strigosa*. The sequences of the repeat library were aligned to the barley (*Hordeum vulgare*) centromeric retrotransposon *cereba* using Blastn to identify the centromere cores in the *A. strigosa* genome^65^.

Full-length LTR retrotransposons were predicted by LTR_retriever^66^, which integrates the results of LTRharvest (version1.5.10)^67^ and LTR_FINDER-1.0.7^68^ and filters out the false positive LTR-RTs to obtain a high-quality LTR-RT library. RepeatMasker was then implemented to identify homologous LTR elements in the *A. strigosa* genome using this LTR-RT library. MITE Tracker^69^ was used to identify miniature inverted-repeat transposable elements (MITEs) (Fig. 1A). Microsatellites were identified using the MIcroSAtellite Identification Tool (MISA)^70^. Only microsatellites of 2–6 nucleotide motifs with at least 5 repeat units were collected.

### Identification of non-coding RNA genes

miRNA, snRNA, C/D snoRNA and H/ACA snoRNA genes were predicted using the CMsearch programme of INFERNAL-1.1 software^71^ against the Rfam database (release 11.0, 2,207 families) (http://rfam.xfam.org/) with a cutoff score of 90 and an *E*-value cutoff of 1e−10. The tRNAScan-SE-1.3 algorithm^72^ with default parameters was used for prediction of tRNA genes in the sorghum, maize, rice, Brachypodium, barley, wheat and *A. strigosa* genomes.

### Gene prediction and annotation of gene function

All predicted gene structures were integrated into consensus gene structures using the EVM annotation pipeline^15^ through the following steps: (A) the prediction software tools Augustus-3.2.1^73^ and FgeneSH++ (http://www.softberry.com) with gene model parameters trained from monocots were used in ab initio gene prediction to build the preliminary gene models on repeat-masked genome sequence; (B) protein homology detection and intron resolution was carried out using Exonerate-2.2.0-x86_64 software^74^ against the protein sequences of *A. tauschii* (downloaded from http://aegilops.wheat.ucdavis.edu/ATGSP/annotation/), *Triticum aestivum* (downloaded from ftp://ftp.ensemblgenomes.org/pub/plants/release-42/fasta/triticum_aestivum)*, T. urartu* (downloaded from http://www.mbkbase.org/Tu/), *H. vulgare* (downloaded from http://edal.ipk-gatersleben.de/repos/pgp/) and *Oryza sativa* (downloaded from Gramene http://www.gramene.org/); (C) previously generated RNA-seq data for different *A. strigosa* S75 tissues (root, root tips, leaf, panicle, shoot and spikelet)^9^ was filtered and then inputted into the genome-guided assembler StringTie^75^ and the de novo assembler Trinity-2.1.1^76^; (D) PASA^77^ was used to reassemble the transcripts based on overlapping alignments from full-length cDNAs and RNA-seq assemblies; (E) the outputs were merged by EVM-1.1.1 to yield a consensus gene set consisting of 76,701 gene models.

Two strategies were then taken to improve the accuracy of these preliminary gene models. First, the gene models were aligned to the barley and wheat (*T. urartu*) gene model sets with BLASTP with an *E*-value cutoff of 1e−20 and gene models with a coverage cutoff of 50% retained. Second, the Illumina RNA-seq sequences from the six different tissues^9^ were mapped to the coding sequences with Bowtie2^64^ and the read depth of each base position and the coverage profiles of these genes was counted using SAMtools-1.5^78^. The gene models with homologue coverage of the coding regions of ≥50% or with RNA reads mapped onto ≥50% of the coding region were retained. In combination, the alignments of the transcriptome and homology data yielded a total of 39,885 HC genes (Table 1; Supplementary Table 3; Supplementary Fig. 6). The completeness of the gene annotations was evaluated by searching the entire annotation using BUSCO software with a set of 1375 BUSCO genes (Supplementary Table 5). The motifs and domains of genes were determined by InterProScan version 5.7^79^ against protein databases including ProDom, PRINTS, Pfam, SMART, PANTHER and PROSITE. The functional ontology for each gene was retrieved from the outputs of InterPro using Gene Ontology^80^. Expression levels of HC genes were also calculated. Raw RNA-seq reads^9^ were downloaded from http://db.ncgr.ac.cn/oat/RNAseq.php. After clipping the adaptor sequences and removing the low-quality reads, RNA-seq reads with two biological replicates were mapped to the coding sequences of HC genes by HISAT2 v2-2.0.5^81^ using default parameters. Normalized read counts based on gene annotations were identified using the R package DESeq2^82^.

### Estimation of divergence time between *A. strigosa* and other grass species

The peptide sequences and CDSs of 2411 single copy orthologous gene clusters from *A. tauschii*, *T. urartu*, *H. vulgare*, *O. sativa*, *Brachypodium distachyon*, *Sorghum bicolor* and *Zea mays* identified by OrthoMCL (v2.0.9)^83^ and collinearity analysis were downloaded from Gramene (http://www.gramene.org/). For genes with alternative splice variants only the longest transcript was selected. The synonymous substitution rate (Ks) of gene pairs was calculated based on the MA model in KaKs_Calculator1.2^84^. The Ks distribution of the one-to-one orthologous pairs of *A. strigosa*–*Z. mays*, *A. strigosa*–*B. distachyon, A. strigosa*–*O. sativa*, *A. strigosa*–*S. bicolor*, *A. strigosa*– *A. tauschii*, *A. strigosa*–*T. urartu* and *A. strigosa*–*H. vulgare* suggested the different divergence times between *A. strigosa* and other grass genomes. The mean Ks was used to estimate the divergence time between different genomes (Supplementary Fig. 13) using a substitution rate of 6.5 × 10^−9^ mutations per site per year.

### Comparison genomics analysis

Genome annotation data were downloaded as follows:

*A. tauschii:* http://aegilops.wheat.ucdavis.edu/ATGSP/annotation/;

*T. urartu*: http://www.mbkbase.org/Tu/;

*A. atlantica:* https://genomevolution.org/coge/GenomeInfo.pl?gid=53337;

*A. eriantha*: https://genomevolution.org/coge/GenomeInfo.pl?gid=53381;

*T. turgidum*: ftp://ftp.ensemblgenomes.org/pub/plants/release-45/fasta/triticum_turgidum/;

*T. aestivum*: ftp://ftp.ensemblgenomes.org/pub/plants/release-42/fasta/triticum_aestivum.

The genome assembly of *A. sativa* was downloaded from https://wheat.pw.usda.gov/GG3/graingenes_downloads/oat-ot3098-pepsico. The gene models of *A. sativa* were predicted by FgeneSH++ (http://www.softberry.com) with gene model parameters trained from monocots. To investigate chromosomal structure variation between *A. strigosa* and diploid wheat (*A. tauschii* and *T. urartu*), tetraploid wheat (*T. turgidum*), hexaploid wheat (*T. aestivum*), hexaploid oat (*A. sativa*) and other diploid oat species (*A. atlantica* and *A. eriantha*), software MCscan (Python version, https://github.com/tanghaibao/jcvi/wiki/MCscan-(Python-version)) was used to identify orthologous blocks and visualise macrosynteny with default parameters. We also searched the collinear paralogous relationship in oat using MCscan (Python version) (Fig. 1A). The Circos software (v0.69)^85^ was used to illustrate the positional relationships among syntenic blocks and genomic features in *A. strigosa* genome.

### Characterisation and evolutionary analysis of the avenacin cluster

RepeatMasker was implemented to identify homologous LTR elements in the genome of *A. strigosa* using the LTR-RT library annotated by LTR_retriever, and the regions masked by arms of the LTR library and full-length LTR-RTs in the region encompassing the avenacin biosynthetic gene cluster retrieved. There are a large number of LTR-RTs (e.g. AS02_069:323541..325997_LTR) at the ends of the three scaffolds shown in Fig. 2A (AS02_290, AS02_289, AS02_026), which may be the reason why the gaps between the three scaffolds were not bridged. The collinearity in the region of the avenacin biosynthetic gene cluster was retrieved from the synteny between *A. strigosa* and diploid, tetraploid and hexaploid wheat, and also other diploid oat species, and then microsynteny of this region was visualised with the software MCscan (Python version) (Supplementary Figs. 15, 20 and 21). Identification of orthologous blocks and visualisation of macrosynteny between *A. strigosa* and *B. distachyon*, rice, barley and wheat (DD genome) was carried out using the software MCscan (Python version). The genes in the subtelomeric region at the end of the long arm of chromosome 1 (Supplementary Data 1) were aligned to those of *B. distachyon*, rice, barley and wheat (DD genome) by Blastp with an *E*-value of 1e−20. The identity heatmap of these genes with their closest homologues in *B. distachyon*, rice, barley and wheat (Fig. 4B) was drawn using the Pheatmap package for R (https://cran.r-project.org/web/packages/pheatmap/index.html).

The evolutionary rates of the avenacin gene cluster and flanking genes when *A. strigosa* is compared with each of the two other sequenced diploid oat species, *A. atlantica* and *A. eriantha*, was estimated by pair-wise comparisons using codeml from PAML 4.9. The avenacin gene cluster is more conserved between *A. strigosa* and *A. atlantica* than between *A. strigosa* and *A. eriantha* in terms of gene order and copy number. We therefore estimated pair-wise dN/dS ratios not only between orthologous sequences (as for *A. strigosa* versus *A. atlantica*), but also for duplicated homologous sequences (as for *A. strigosa* versus *A. eriantha*). To obtain the maximum likelihood estimates of ω, we first set the control file as: runmode = −2, model = 0, NSsites = 0, Fix_omega = 0; and then run it as: runmode = −2, model = 0, NSsites = 0, Fix_omega = 1, Omega = 1. The log likelihood values from the two executions were subtracted. The negative of twice of this value was used for likelihood ratio test. Statistical significance was assessed using a chi-square (*χ*^2^) distribution with one degrees of freedom. Only dN/dS ratio with *p* < 0.05 were considered as significant and plotted (see Supplementary Fig. 24).

### Flow sorting of chromosome 1 and amplification of chromosomal DNA

Chromosome 1 was purified by flow cytometric sorting. Suspensions of intact mitotic metaphase chromosomes were prepared from synchronised root tips^86^ and stained by DAPI at 2 μg/ml. The samples were analysed using a FACSAria II SORP flow cytometer and sorter (BD Biosciences, San Jose, USA) and the resulting distribution of relative fluorescence intensity (flow karyotype) comprised three major peaks (Supplementary Fig. 12). A total of 50,000 chromosomes were flow sorted from the peak indicated in Supplementary Fig. 12b into 40 μL of sterile deionized water in 0.5 ml PCR tubes. Chromosomal DNA was amplified using the Illustra GenomiPhi V2 DNA Amplification Kit (GE Healthcare Bio-Sciences) according to Šimková et al.^87^, yielding a total of 8.63 μg DNA. The identity of flow-sorted chromosome 1 was confirmed by PCR using 500 chromosomes sorted from the selected peak as template and two set of primer pairs, Amy109 and Amy610, which amplify 272 bp and 329 bp segments of the *Sad1* gene, respectively (Amy109F: TATCCATTATGACGACGAATCAACC; Amy109R: tctataccaacCTGTGCCTTCATTCC; Amy610F: GTCGCTACATTTACAATCAACAGgcat; Amy610R: catacCGACAACCATATTTTTCCCC). The chromosome identity was independently confirmed by analysing 1000 chromosomes, which were sorted from the selected peak onto a microscopic slide and subjected to FISH with a digoxigen-labelled probe for the *Sad1* gene. The sites of probe hybridisation were detected using anti-digoxigenin FITC conjugate and the chromosomes were counterstained by DAPI (Supplementary Fig. 12). The DNA template from chromosome 1 was sequenced in six Illumina Hiseq lanes, and 101 Gb paired-end reads (150 bp) were generated. The paired-end reads were aligned to the seven assembled *A. strigosa* chromosomes using bwa-0.7.17 with default parameters^88^. Reads lacking primary alignment (SAM Flag 256) and with mapping quality below 30 were filtered out. The distribution of these paired-end reads on the seven chromosomes was investigated to verify the accuracy of Hi-C assembly.

### Karyotyping and DNA fluorescence in situ hybridisation

The preparation of mitotic metaphase spreads and subsequent FISH was carried out as described in Rey et al.^89^. The *Sad1* gene sequence was split into three parts: a 3 kb promoter region, a region spanning from the ATG translation initiation site to the stop codon (including the introns), and a 1 kb terminator region. These sequences were domesticated for the use of the Golden Gate modular cloning technology (Moclo)^90^ by introducing silent, single nucleotide mutations to remove any endogenous *Bpi*I, *Bsa*I, *Esp*31 and *Dra*III restriction sites. Level 0 modules corresponding to these gene parts were synthetised using the Invitrogen GeneArt Gene Synthesis service (Thermo Fisher Scientific) and were subsequently used for the assembly of level 1 vectors, using the pL1-R2 pICH47811 vector backbone^90^. The *Sad1* gDNA sequence was excised from the pL1-R2 backbone using the *Dra*III restriction enzyme (Supplementary Data 3), then separated on a 1% agarose gel and retrieved using the QIAquick Gel Extraction Kit (Qiagen). The *Sad1* probe was labelled with DIG-11-dUTP (Sigma) using the DIG-nick translation mix (Sigma) and detected with mouse anti-digoxigenin antibody (Abcam) followed by anti-mouse Fab fragments conjugated with Alexa Fluor 488. The ribosomal sequences pTa71 and pTa794 were synthesized according to Rey et al.^89^. pTa71 was labelled with ChromaTide Alexa Fluor 568-dUTP (Thermo Fisher Scientific) and pTa794 was labelled with biotin-16-dUTP (Sigma) by nick translation. pTa794 was detected with Streptavidin-Alexa Fluor 647 (Thermo Fisher Scientific).

For meiotic pachytene spread preparation, one of the three synchronised anthers present in each floret was squashed in 0.1% acetocarmine stain and examined under the light microscope to identify the pachytene stage of meiosis. The two remaining anthers were fixed in freshly prepared 3:1 absolute ethanol/glacial acetic acid (v/v) for 2 h at room temperature, then re-fixed with fresh fixative, and stored at 4 °C until use. Spread preparation and FISH were carried out following the method of Cabrera et al.^91^. The telomere repeat sequence probe was amplified by PCR^92^ and labelled with biotin-16-dUTP using the Biotin-nick translation mix (Sigma, St. Louis, MO, USA) according to the manufacturer’s instructions. The *Sad3* probe was generated following the exact same procedure as for *Sad1 and* was labelled with DIG-11-dUTP using the DIG-nick translation mix (Sigma). The *Sad1* probe was labelled with tetramethyl-rhodamine-5- dUTP (Sigma) by nick translation^91^. Biotin-labelled probes were detected with Streptavidin-Cy5 (Thermo Fisher Scientific, Waltham, Massachusetts, USA). Digoxigenin-labelled probes were detected with anti-digoxigenin-fluorescein Fab fragments (Sigma).

Images were acquired using 63× and 100× NA1.4 oil objectives on a Leica DM5500B microscope equipped with an X-Cite 200DC (Lumen Dynamics) metal halide light source and a Hamamatsu ORCA-FLASH4.0 camera and controlled by Leica LAS X software v2.0. Images were processed using Fiji (an implementation of ImageJ, a public domain programme by W. Rasband available from http://rsb.info.nih.gov/ij/) and Adobe Photoshop CS4 (Adobe Systems Incorporated, USA) version 11.0 × 64.

### Transient expression in *Nicotiana benthamiana*

AsCYP72A476 had previously been cloned into the pEAQ-HT-DEST1 expression vector^93^ as part of an earlier investigation of avenacin CYPs^94^. For reconstitution of the avenacin pathway in *N. benthamiana*, expression constructs were made using Golden Gate technology^95^. Each coding sequence was domesticated for use in the Golden Gate modular cloning system^90,95^, synthesized, and then inserted into pMS (GeneArt) to form sequence-verified level 0 standard parts. In order to optimise the levels of expression of the genes of interest, the CPMV hypertrans (CPMV-*HT*) system^96^ was domesticated and incorporated into two extra level 0 modules: EC80361-pL0M-CPMV-HT 5′UTR and EC81088-CPMV-HT 3′UTR-T-35S. For each avenacin A-1 biosynthetic gene, GoldenGate assembly was used to generate CaMV35S promoter—CPMV-*HT* 5′ UTR—GOI CDS—CPMV-*HT* 3′ UTR- CaMV35S terminator transcription units, with the exception of the *SCPL1/Sad 7* gene, which was included in a CaMV35S promoter—TMV Omega UTR—Sad7 CDS—*Sad7* terminator transcription unit. The individual transcription units were combined to produce a set of three binary expression vectors (Supplementary Data 4): EC80344 (*bAS1/Sad1* + *CYP51H10/Sad2* + *CYP72A475/Sad6* + *CYP94D65* + *CYP72A476*); EC80345 (*AAT1* + *UGT91G16* + *TG1* + *P19*) and EC80379 (*MT1/Sad9* + *UGT74H5/Sad10* + *SCPL1/Sad7*)^3–8,19,20^. In the three vectors, position 1 corresponds to a pNOS:Kanamycin selection cassette, EC15029, provided by the Engineering Nitrogen Symbiosis for Africa (ENSA) project (https://www.ensa.ac.uk/). A p19 gene (suppressor of gene silencing), under the control of CaMV35S promoter and CaMV35S terminator was also incorporated into the EC80345 and EC80379 binary vectors for optimal expression of the avenacin A-1 biosynthetic pathway.

*N. benthamiana* plants were grown in greenhouses maintained at 23–25 °C with 16 h of supplementary light per day and agro-infiltration carried out as described in Reed et al.^97^. In brief, expression constructs were transformed into *Agrobacterium tumefaciens* strain GV3101 and the strains infiltrated into *N. benthamiana* leaves. To co-express combinations of genes, *A. tumefaciens* strains containing different expression constructs were diluted to 0.9 OD_600_ and mixed in equal volumes to result in a final concentration per strain of 0.1 OD_600_. A strain containing a construct for expression of HMG-CoA reductase, HMGR (GenBank accession number—KY284573), was included in all combinations to increase triterpene production^97^. Leaves were harvested 5 days after agro-infiltration and freeze-dried. Freeze-dried leaf material (20 mg per sample) was ground twice at 1000 rpm for 30 s (Geno/Grinder SPEX Sample Prep 2010). After centrifugation at 13,000 × *g* for 10 s, the ground leaf material was extracted with 1 ml of 80% MeOH with 20 μg/mL digitoxin standard (Merck) at 18 °C for 20 min with shaking at 1400 rpm (Thermomixer Comfort, Eppendorf). Samples were centrifuged at 20,000 × *g* at 4 °C for 2 min and 0.8 ml of supernatant was partitioned twice with 400 μL hexane on ice. Aqueous fractions were dried in a Genevac EZ-2 Elite centrifugal evaporator maintaining the temperature below 30 °C and stored at −80 °C.

For high-performance liquid chromatography (HPLC), samples were resuspended in 75 μL methanol and filtered through Corning®Costar®Spin-X®centrifuge tube filters (Sigma-Aldrich). The filtrate (50 μL) was combined with 50 μL 50% MeOH and 10 μL aliquots analysed by reverse phase HPLC using a 50 × 2.1 mm 2.6 μ Kinetex XB-C18 column (Phenomenex). The gradient of 100% water (Buffer A) versus 100% acetonitrile (Buffer B), run at 0.3 ml/min and 30 °C was: 20% Buffer B from 0–3 min; 20–60% Buffer B from 3–28 min; 60–100% Buffer B from 28–30 min; 100% Buffer B between 30–33 min; 100 to 20% Buffer B from 33–34 min, and held at 20% Buffer B until 35 min. Avenacins were detected by fluorescence (353 nm excitation, 441 nm emission) and mass spectrometry (Shimadzu LC-2020 dual source MS, dual ESI/APCI ionisation) collected in positive and negative modes from *m/z* 50–1500. Non-fluorescent compounds were analysed as for avenacins with the following changes: the gradient was 15% Buffer B from 0 to 1.5 min; 15–60% Buffer B from 1.5 to 26 min; 60–100% Buffer B from 26 to 26.5 min; 100% Buffer B between 26.5 and 28.5 min; 100–15% Buffer B from 28.5 to 29 min, and held at 15% Buffer B until 30 min, detection was by charged aerosol detector (CAD, Corona Ultra RS from Dionex), and MS was collected in negative mode.

The purification of 23-hydroxy-β-amyrin was based on the method of Reed et al.^97^. Briefly, freeze dried leaves (91 g) were extracted using a Büchi SpeedExtractor 914 using 3 cycles of 100 % ethyl acetate, cycle 1: 0 min hold time, cycles 2 and 3:5 min hold time, with a 2 min solvent flush and a 12 min air flush. The extract was dried, dissolved in ethanol, treated with 50 ml Ambersep 900 hydroxide form ion-exchange resin (Sigma) and agitated at room temperature by slow rotation for 30 min as the resin changed colour from pink to green. This step was repeated twice more until the colour change of resin was no longer observed. The mixture was filtered through a column of diatomaceous earth and the column washed with 200 ml ethanol, 200 ml 1:1 ethanol hexane mixture, and 200 ml hexane. The washes were combined, adsorbed onto silica and dry-loaded onto a SNAP Ultra 50 g cartridge (Biotage) for column chromatography using a Biotage Isolera One. The mobile phase gradient was as follows: Solvent A: [hexane] Solvent B: [ethyl acetate]; gradient 5% [B] to 100% [B] over 15 column volumes collecting 90 mL fractions. Fractions were analysed by TLC and those identified as containing the product of interest (fractions 7–10) were combined and dried. The dried extract from fractions 7–10 (~370 mg) was subjected to a further round of flash chromatography using a KP-Sil 25 g cartridge (Biotage). The mobile phase was as follows: Solvent A [dichloromethane] Solvent B [ethyl acetate]; Isocratic 20% [B] over 31 column volumes collecting 22 ml fractions. The fractions containing the highest content of 23-hydroxy-β-amyrin were determined by TLC and GC-MS^97^ and fractions 30–40 were pooled and dried giving 59 mg of a pale yellow crystalline solid. Finally, recrystallisation was performed using hot methanol, yielding a total of 30.2 mg of pure 23-hydroxy-β-amyrin as white needle-like crystals.

The purified product of CYP94D65 was subjected to NMR analysis in CDCl_3_. NMR spectra were recorded in Fourier transform mode at a nominal frequency of 600 MHz for ^1^H NMR, and 150 MHz for ^13^C NMR. Chemical shifts were recorded in ppm and referenced to an internal TMS standard. The product was determined to be 23-hydroxy-β-amyrin. This assignment was made via a combination of ^1^H, ^13^C, DEPT-135, DEPT-edited HSQC, COSY, HMBC and 2D NOESY experiments (Supplementary Table 8). The spectra were also found to be consistent with the literature^94^.

### Metabolite analysis of root and leaf extracts of different *Avena* accessions

Twenty seeds of each accession were dehusked and sterilised by washing in 5% sodium hyperchlorite solution followed by 3 washes in distilled water. The seeds were placed on distilled water agar and kept at 4 °C for 2 days before transferring to a growth cabinet for germination (22 °C 16 h/8 h day/night cycle). After 5 days, roots and leaves were excised and placed in 1 ml 100% MeOH along with 2 tungsten beads and ground at 14,000 rpm for 2 min (Geno/Grinder SPEX Sample Prep 2010). The samples were centrifuged at 1600 × *g* for 10 min and the supernatant dried down by vacuum centrifugation and resuspended in 100% MeOH at 10 µl per 5 mg tissue. For TLC analysis, 10 µl aliquots were loaded onto silica gel 60 TLC plates and the TLCs run in chloroform:methanol:water (13:6:1 v/v) before visualisation under UV illumination. For LC–MS analysis, 5 µl aliquots were analysed by reverse phase HPLC as described above for fluorescent compounds produced in *N. benthamiana*. For high-resolution LC–MS analysis, 5 µL aliquots were analysed on a Vanquish UHPLC, followed by a flow-splitter supplying a CAD and a QExactive Orbitrap MS. Separation was performed on a Kinetex column 2.6 μm XB-C18 100 Å, 50 × 2.1 mm (Phenomenex) and the gradient of 0.1% formic acid in water (Buffer A) versus 0.1% formic acid in acetonitrile (Buffer B), run at 0.6 ml/min and 40 °C was: 15% Buffer B from 0–0.75 min; 15–60% Buffer B from 0.75 to 13 min; 60–100% Buffer B from 13 to 13.25 min; 100% Buffer B between 13.25 and 14.25 min; 100 to 15% Buffer B from 14.25–14.5 min, and held at 15% Buffer B until 16.5 min. The CAD evaporator temperature was 35 °C, and it collected data at 10 Hz with a 5 s filter constant. The MS was set up to carry out full MS and data-dependent MS2 (top 3 precursor ions), in negative mode. The full MS scans were *m/z* 200–2500 with a resolution of 70,000, 3 × 10^6^ ions automatic gain control (AGC) target, and a maximum ion time of 100 ms. MS2 scans were at 17,500 resolution with 1 × 10^5^ AGC target, 50 ms maximum ion time, an isolation window of *m/z* 4.0, and 30% normalised collision energy. Once an ion had been selected as a precursor, it was excluded (dynamic exclusion) in favour of less abundant ions for 5 s. Ionisation was by electrospray in negative mode: Spray voltage 2800 V, 320 °C capillary temperature, 9 units sheath gas, 0 units aux gas. Data analysis was carried out in FreeStyle 1.6 (Thermo).

### plantiSMASH and cluster density analyses

For cluster density analysis, the plantiSMASH algorithm^30^ and published genome annotations were used to determine locations of gene clusters and genes, respectively. Cluster density scores were calculated for a 100 Mb-sized sliding window, slide size 10 Mb. The score per 100 Mb region was calculated as the number of clusters in region/the number of genes in region. For co-expression analysis of plantiSMASH-predicted biosynthetic gene clusters, Pearson correlation coefficient values (*r*-val) were calculated from DESeq2-normalised RNA-seq data^9^. A representative gene from each cluster was used as bait for co-expression analysis. Gene expression heatmaps were generated from *Z*-scores derived, per each gene, from the same RNA-seq data described above, with Morpheus (https://software.broadinstitute.org/morpheus/) (Supplementary Data 5).

### Reporting summary

Further information on research design is available in the Nature Research Reporting Summary linked to this article.

## Supplementary information

Supplementary Information

Supplementary Data 1

Supplementary Data 2

Supplementary Data 3

Supplementary Data 4

Supplementary Data 5

Description of Additional Supplementary Files

Reporting Summary

## Data Availability

Data supporting the findings of this work are available within the paper and its Supplementary Information files. A reporting summary for this article is available as a Supplementary Information file. The datasets and plant materials generated and analysed during the current study are available from the corresponding authors on request. DNA sequence data can be found in the European Nucleotide Archive under accession number PRJEB25739 which includes: ERS2620538, whole-genome shotgun sequence, Oxford Nanopore sequence, optical map and Hi-C data for *A. strigosa* accession S75; ERS2620540, flow-sorted chromosome 1 sequence; ERS2620539, RNA-seq data (the RNA-seq data was published previously^9^). Raw and assembled RNA-seq data can also be accessed at http://db.ncgr.ac.cn/oat/RNAseq.php and sequences can also be found in GenBank (accession nos. MN396758–MN396761). The *A. strigosa* S75 genome and the flow-sorted chromosome 1 assemblies, the BLAST searches and related information are also available from https://figshare.com/s/a2f71d7644c5aa5b09ff and http://www.ncgr.ac.cn/oat. The Rfam database for identification of non-coding RNA genes was downloaded from ftp://ftp.ebi.ac.uk/pub/databases/Rfam/11.0. Embryophyta_odb10 database used for BUSCO was downloaded from https://busco-data.ezlab.org/v4/data/lineages/. Source data are provided with this paper.

## Source data

Source Data
