# Supplementary material for: Subtelomeric assembly of a multi-gene pathway for antimicrobial defense compounds in cereals
Source: Nat Commun. 2021 May 7;12:2563. doi: 10.1038/s41467-021-22920-8 (PMC8105312; doi:10.1038/s41467-021-22920-8)
Supplement: Supplementary file 1 — Supplementary Information [file 41467_2021_22920_MOESM1_ESM.pdf]

Subtelomeric assembly of a multi-gene pathway for antimicrobial defense compounds in cereals

Li *et al.*

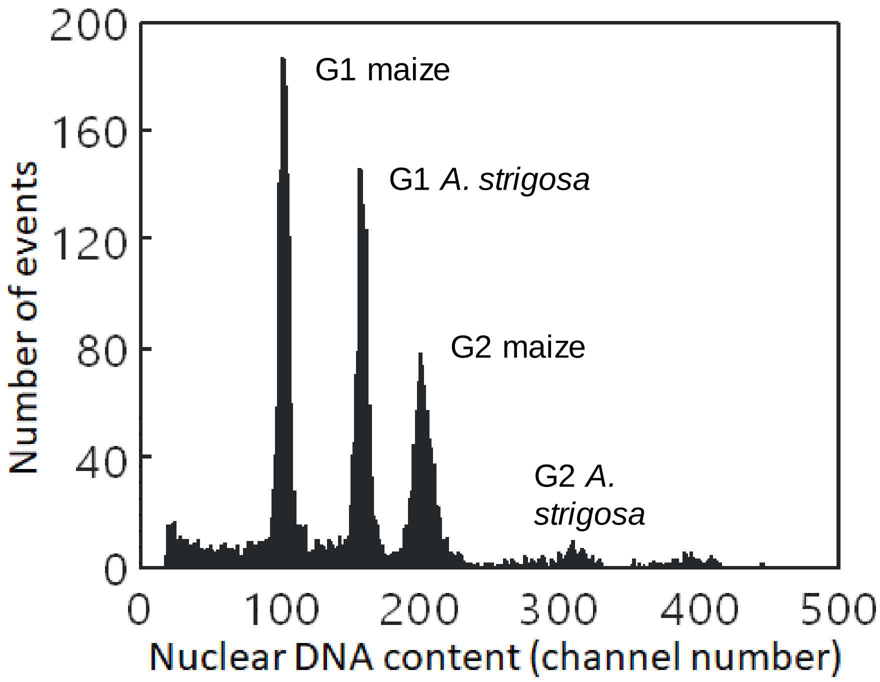

Supplementary Fig. 1. Flow cytometric estimation of the nuclear genome size of *Avena strigosa*. Nuclei were isolated from *A. strigosa* accession S75 and maize (*Zea mays* cv. CE.77; used as an internal reference standard), stained and analyzed simultaneously. The 2C DNA content was calculated using the ratio of the G1 peak means of *A. strigosa* S75 and the maize standard, giving a 2C value for *A. strigosa* S75 of 8.486 ± 0.074 pg DNA (mean ± SD). The 1C genome size in base pairs was calculated using the formula 1pg DNA = 0.978 x10^9 bp[1], giving a 1C *A. strigosa* S75 genome size of 4149 ± 0.036 Mb (mean ± SD).

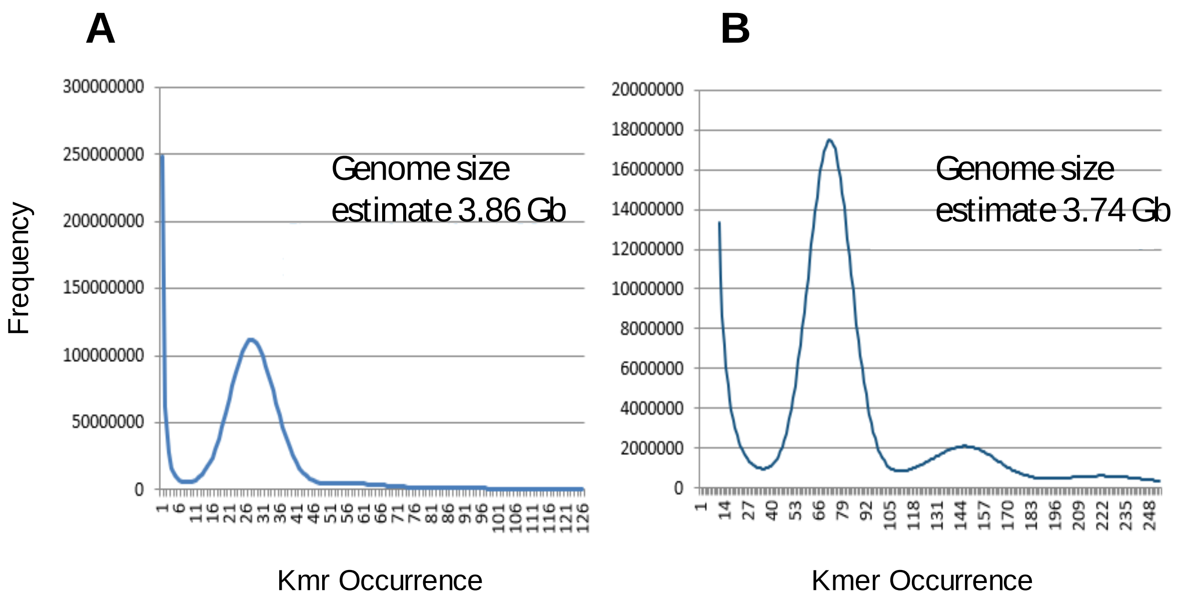

Supplementary Fig. 2. Estimation of the nuclear genome size of *A. strigosa* accession S75 by K-mer analysis. *k*-mer values were plotted against the frequency (y-axis) at their occurrence (x-axis). Genome size was estimated by Phusion2 (**A**) and kmerFreq_AR (**B**).

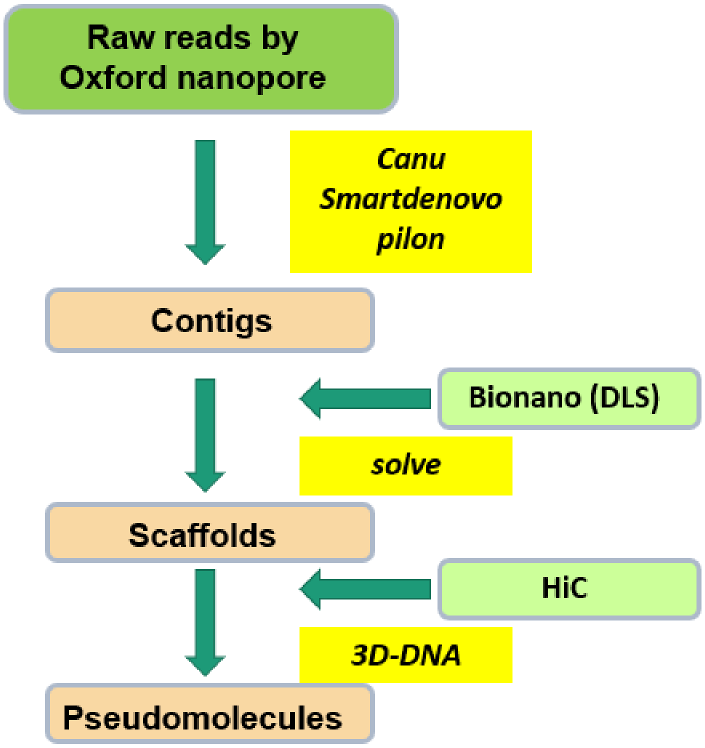

Supplementary Fig. 3. Strategy for assembly of the *A. strigosa* S75 genome sequence

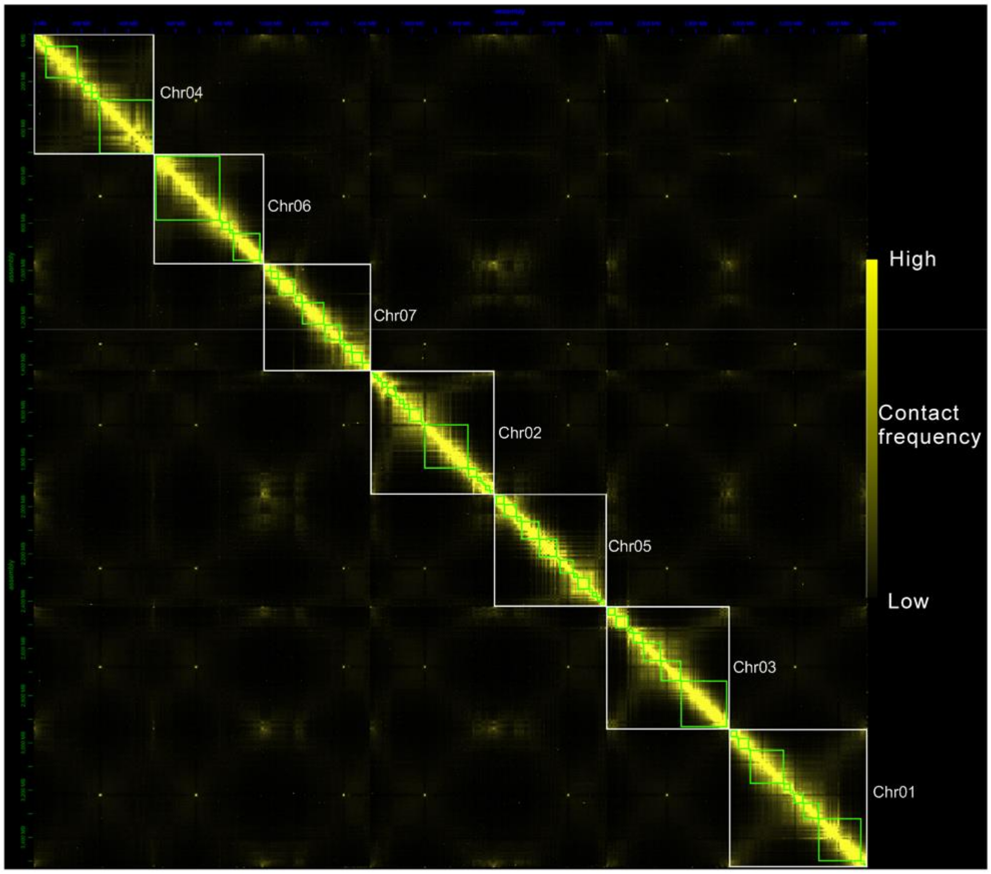

Supplementary Fig. 4. Hi-C contact map for each chromosome. The white and green boxes respectively represent the chromosomes and their corresponding scaffolds.

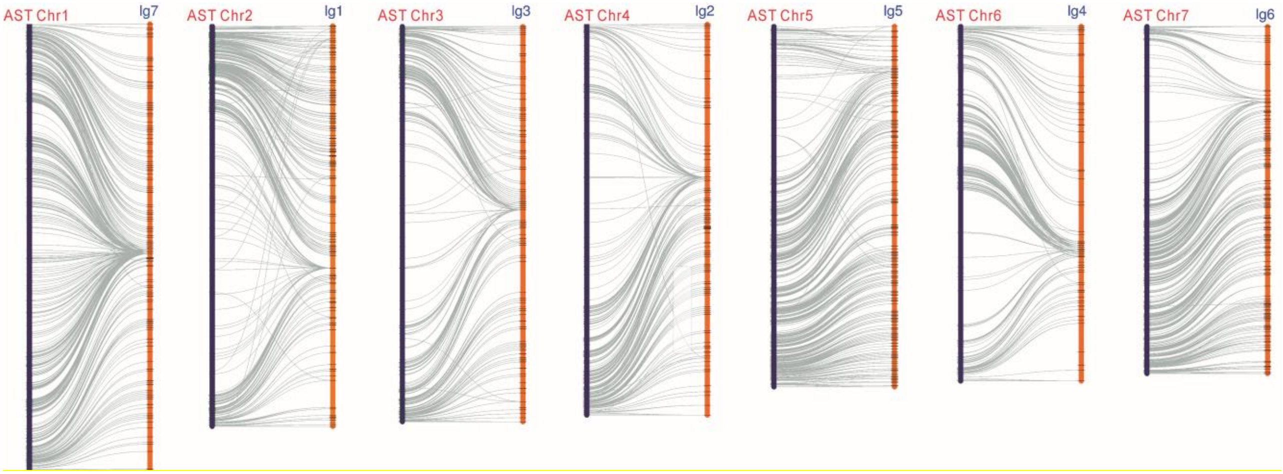

Supplementary Fig. 5. Comparison of the *A. strigosa* accession S75 assembly with a genetic linkage map constructed from an F6:8 recombinant inbred population generated from a cross of the AA genome diploids *A. strigosa* (CI 3815) and *Avena wiestii* (CI 1994)[2]. A total of 5515 64-base tag-level haplotypes out of 13,873 examined had good matches (either perfect or one base mismatch) matches to single sites on the seven chromosomes.

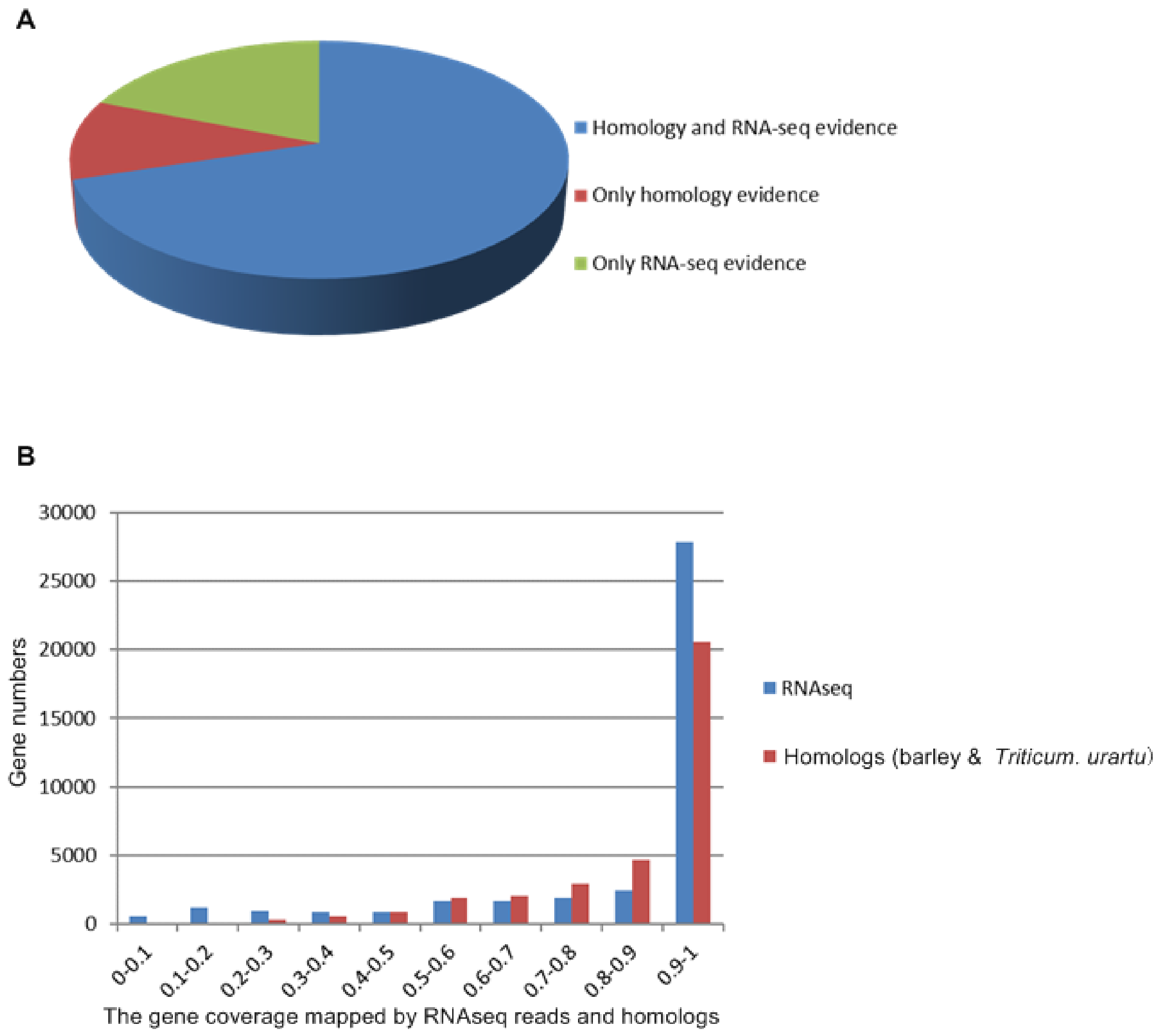

Supplementary Fig. 6. High confidence gene models identified in the *A. strigosa* S75 genome assembly. (**A**) Proportions of gene models supported by evidence from protein-based homology searches and expression data. (**B**) RNA-seq and homolog-based evidence for the high confidence gene models. RNAseq reads were mapped to gene coding sequences. The coverage is the ratio of total aligned reads length to the coding sequence length of the gene. The gene models of *A. strigosa* were aligned to those of barley and *Triticum urartu* (red wild einkorn wheat; AA genome) by Blastp. The coverage of each *A. strigosa* gene is the ratio of aligned protein sequence length to total protein length of the corresponding gene. Source data are provided as a Source Data file.

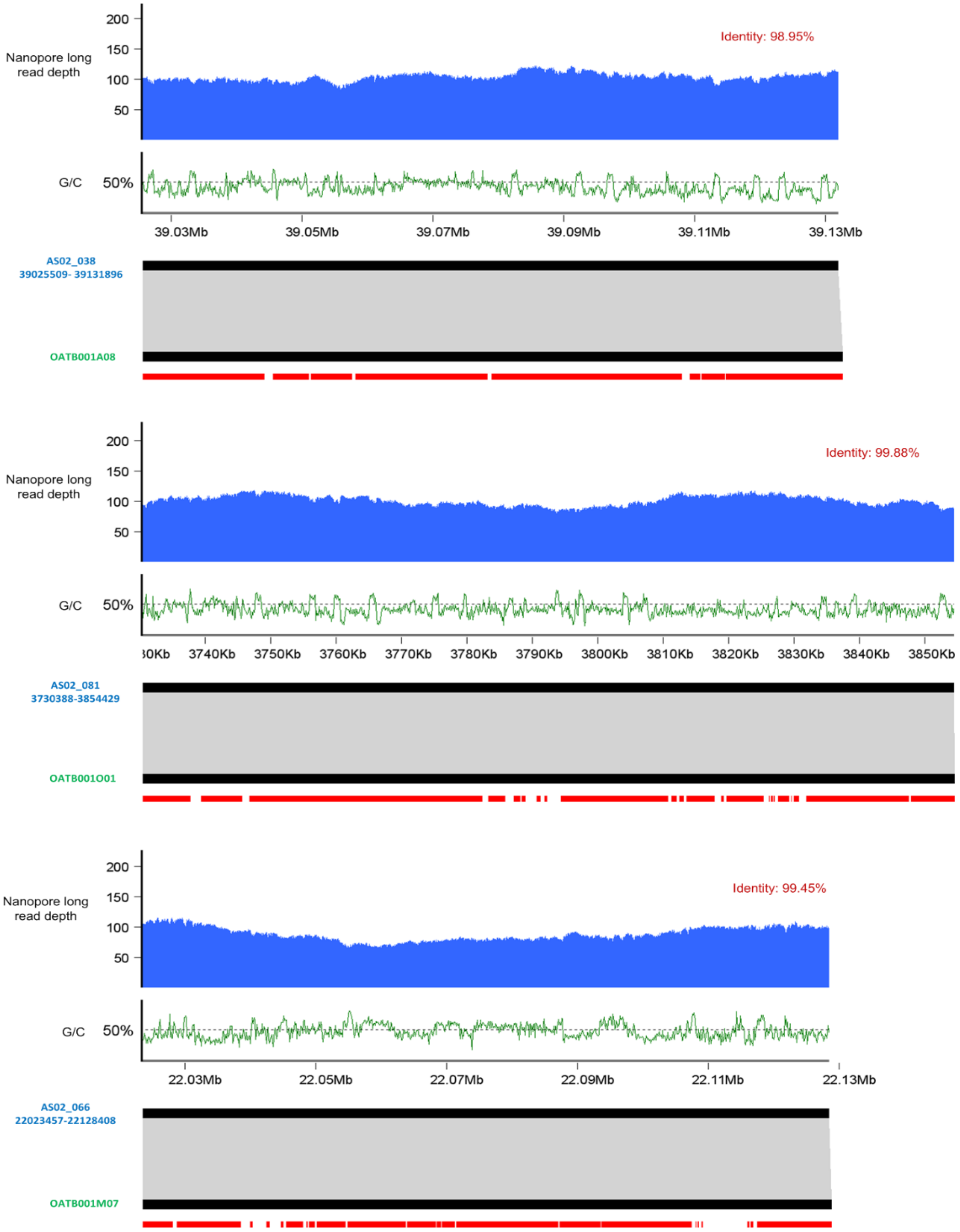

Supplementary Fig. 7. Comparison of assembled scaffolds with *A. strigosa* S75 BACs sequenced using the Sanger method. The depth of the nanopore long reads (blue) was calculated by mapping paired-end reads onto the BAC sequences. The grey blocks show aligned regions between the BAC sequences and the scaffolds. RepeatMasker-annotated transposable elements (Repeats) within the BAC sequences are shown in red. The white blocks indicate the unfilled gaps on the scaffolds. GC content is shown in green.

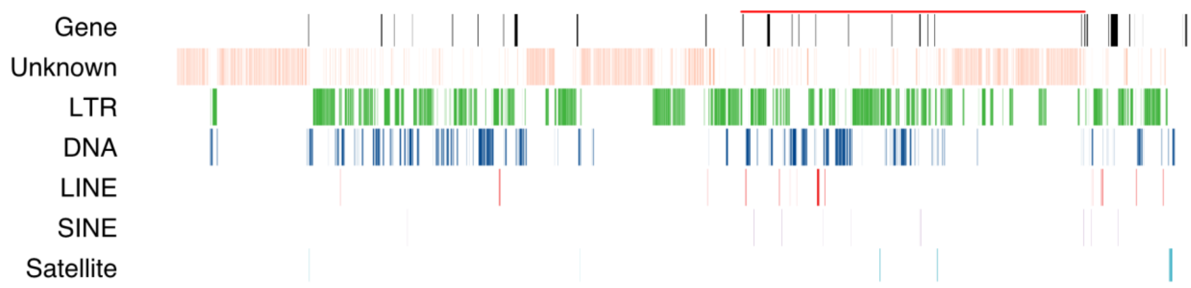

Supplementary Fig. 8. Occurrence of different types of repetitive elements within the avenacin cluster region. The avenacin gene cluster is indicated by the red line.

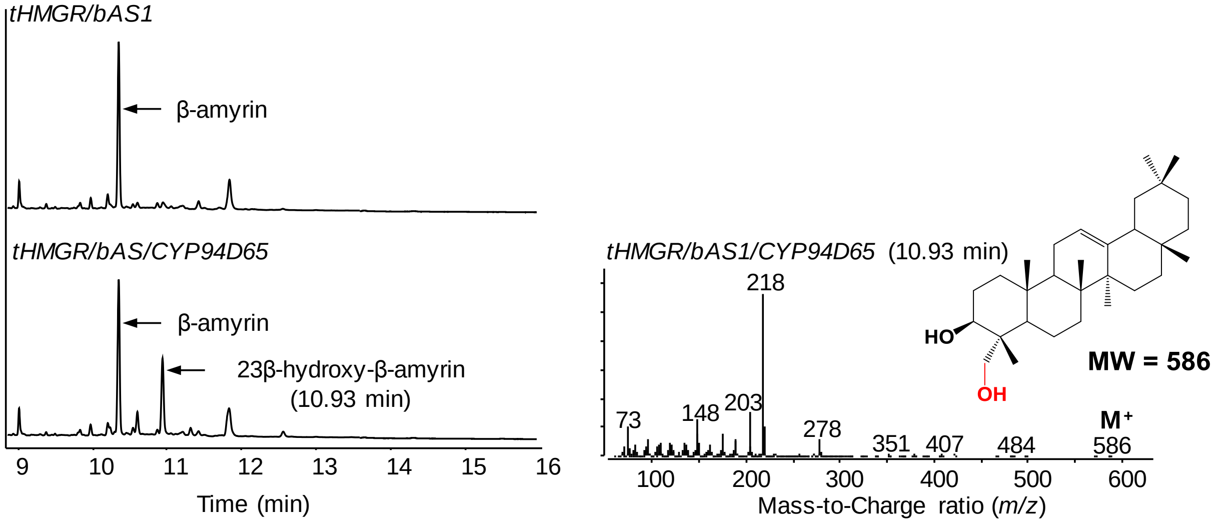

Supplementary Fig. 9. CYP94D65 is a C-23 hydroxylase. Left: GC-MS total ion chromatograms of extracts from *N. benthamiana* leaves expressing *tHMGR* and *bAs/Sad1* without or with CYP94D65. Right: EI mass spectrum and inferred structure for the oxidized form of β-amyrin generated by CYP94D65.

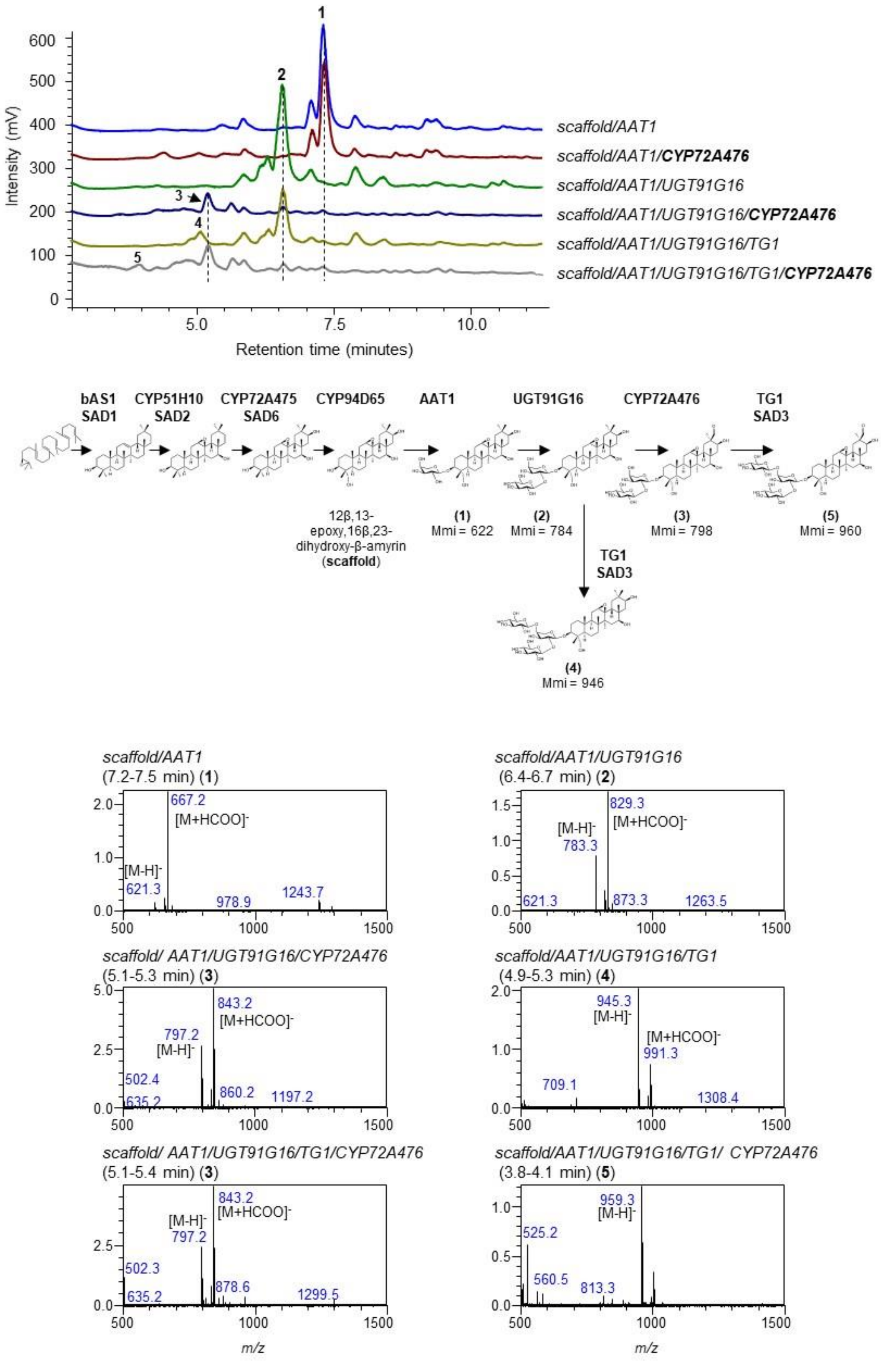
1
2
3
4
5
Intensity (mV)
Retention time (minutes)
scaffold/AAT1
scaffold/AAT1/CYP72A476
scaffold/AAT1/UGT91G16
scaffold/AAT1/UGT91G16/CYP72A476
scaffold/AAT1/UGT91G16/TG1
scaffold/AAT1/UGT91G16/TG1/CYP72A476
bAS1 SAD1
CYP51H10 SAD2
CYP72A475 SAD6
CYP94D65
AAT1
UGT91G16
CYP72A476
TG1 SAD3
12β,13-epoxy,16β,23-dihydroxy-β-amyrin (scaffold)
(1) Mmi = 622
(2) Mmi = 784
(3) Mmi = 798
(5) Mmi = 960
TG1 SAD3
(4) Mmi = 946
scaffold/AAT1 (7.2-7.5 min) (1)
667.2 [M+HCOO]-
[M-H]- 621.3
978.9
1243.7
scaffold/AAT1/UGT91G16 (6.4-6.7 min) (2)
829.3 [M+HCOO]-
[M-H]- 783.3
621.3
873.3
1263.5
scaffold/ AAT1/UGT91G16/CYP72A476 (5.1-5.3 min) (3)
843.2 [M+HCOO]-
797.2 [M-H]-
502.4
635.2
860.2
1197.2
scaffold/AAT1/UGT91G16/TG1 (4.9-5.3 min) (4)
945.3 [M-H]-
[M+HCOO]- 991.3
709.1
1308.4
scaffold/ AAT1/UGT91G16/TG1/CYP72A476 (5.1-5.4 min) (3)
843.2 [M+HCOO]-
[M-H]- 797.2
502.3
635.2
878.6
1299.5
scaffold/AAT1/UGT91G16/TG1/ CYP72A476 (3.8-4.1 min) (5)
959.3 [M-H]-
525.2
560.5
813.3
m/z

Supplementary Fig. 10. CYP72A46 introduces the C-30 aldehyde group. Analysis of *N. benthamiana* leaf extracts from plants co-expressing *HMGR, AsbAS1/Sad1, AsCYP51H10/Sad2, AsUGT99D1/AsAAT1, AsUGT91G16, AsTG1* and *CYP72A476* in different combinations. HPLC-CAD-MS traces for extracts from agroinfiltrated leaves are shown.

Co-expression of *bAS1/SAD1* with the three CYPs, *CYP51H10/SAD2, CYP72A475/SAD6* and *CYP94D65,* and the arabinosyltransferase, *AAT1/UGT99D1* yields 3β-(α-L-arabinopyranosyloxy)-12β,13-epoxy,16β,23-dihydroxy-β-amyrin (**1,** retention time =7.3 min, *m/z* = 621, top left mass spectrum). The addition of CYP72A476 does not result in the accumulation of new peaks, suggesting that CYP72A476 is not active on **1**.

Co-expression of *bAS1/SAD1, CYP51H10/SAD2, CYP72A475/SAD6, CYP94D65* and *AAT1/UGT99D1* with the glucosyltransferase *UGT91G16* results in the disaccharide (**2,** retention time = 6.6 min, *m/z* = 783, top right mass spectrum). The addition of *CYP72A476* results in the clear consumption of the disaccharide peak (**2**) and the appearance of a new more polar peak with the mass of (**2**) with the addition of an aldehyde group (**3,** retention time = 5.2 min, *m/z* = 797, middle left mass spectrum).

Co-expression of *bAS1/SAD1, CYP51H10/SAD2, CYP72A475/SAD6, CYP94D65, AAT1/UGT99D1* and *UGT91G16* with *TG1* results in a reduction of the disaccharide peak (**2**) and the appearance of the trisaccharide (**4,** retention time = 5.1 min, *m/z* = 945, middle right mass spectrum). Co-expression with *CYP72A476* results in the conversion of the disaccharide and trisaccharide peaks to new products, (**3,** retention time = 5.2 min, *m/z* = 797, bottom left mass spectrum) and (**5,** retention time = 4.0 min, *m/z* = 959, bottom right mass spectrum) respectively, that have masses consistent with that of the addition of an aldehyde group.

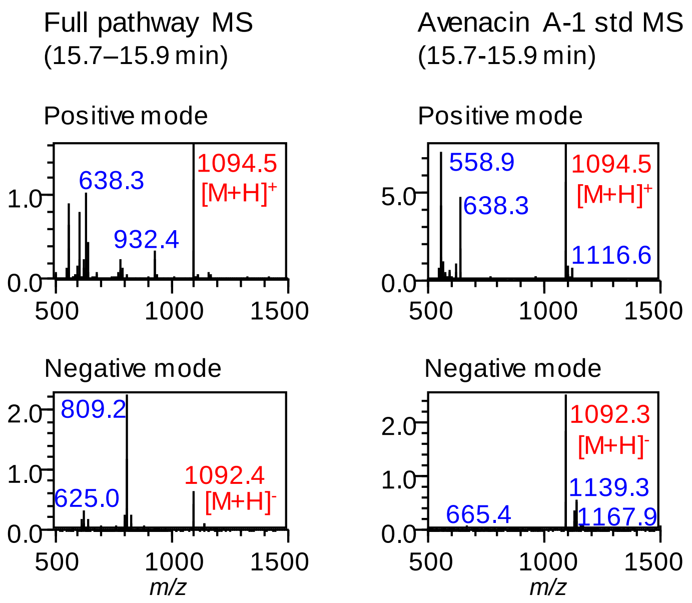

Supplementary Fig. 11. Mass spectra (positive and negative modes) for the peaks at 15.8 min in Fig 2C for extracts from *N. benthamiana* expressing the full avenacin pathway and an avenacin A-1 standard.

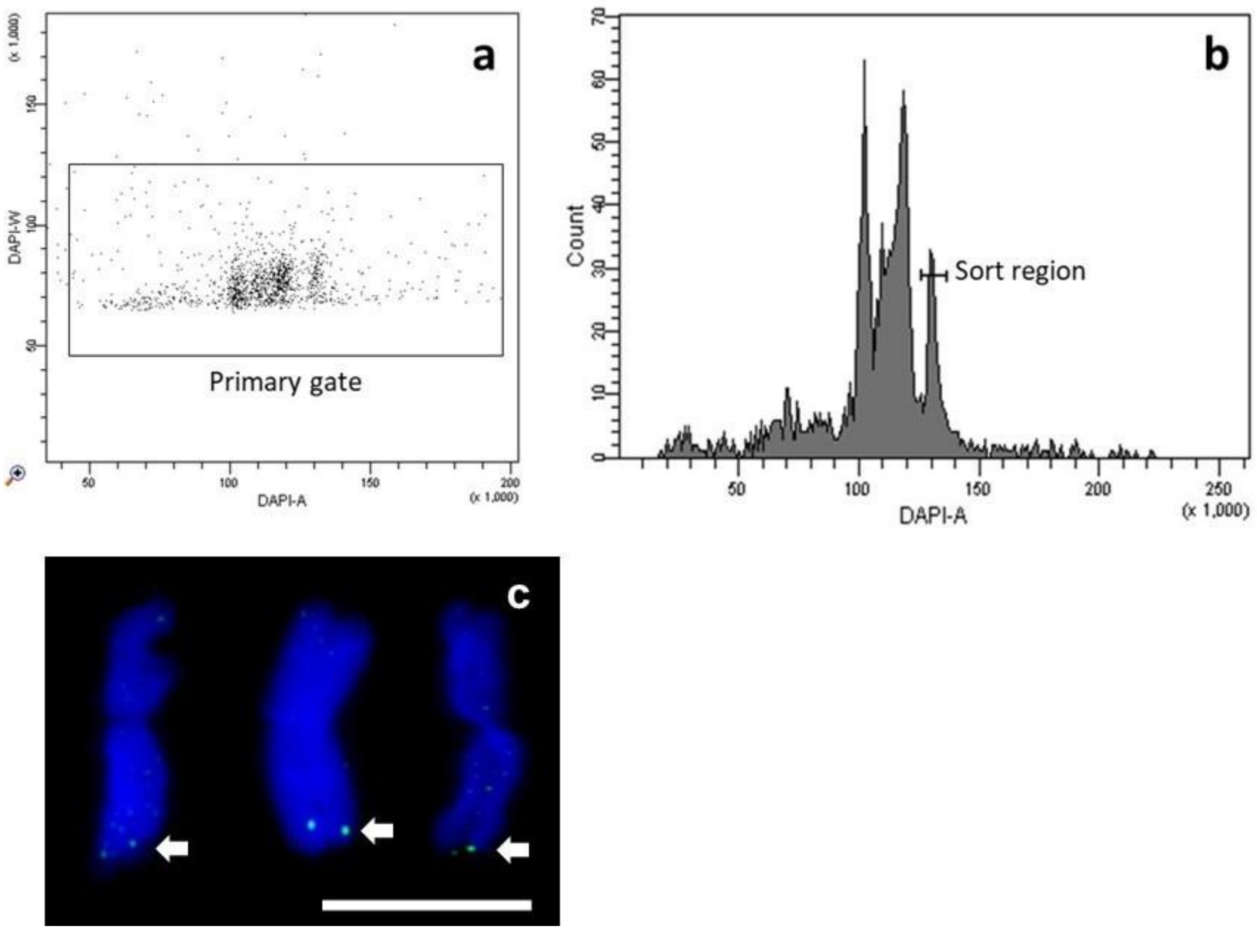

Supplementary Fig. 12. Flow-sorting of chromosome 1 of *Avena strigosa.* (a,b) Gating strategy to sort chromosome 1 of *Avena strigosa.* The primary gate was set on a dot-plot DAPI-W vs DAPI-A to discriminate the population of intact chromosomes **(a)**; subsequent dependent sort region was set on a histogram DAPI-A to sort chromosome 1 **(b)**. **(c)** Images of three flow-sorted chromosomes 1. The chromosomes were identified by fluorescence *in situ* hybridization (FISH) with a probe for the *bAS1/Sad1* gene (yellow-green). The fluorescent signals were weak due to small probe size; their positions are indicated by white arrows. Chromosomal DNA (blue) was stained using DAPI. Bar = 10 μm. A total of 50,000 chromosomes were flow-sorted for the peak shown in **(b)**, from which 8.63 μg DNA was prepared. The identity of the sorted chromosome population was also confirmed by PCR using gene-specific primers (see Methods for further information).

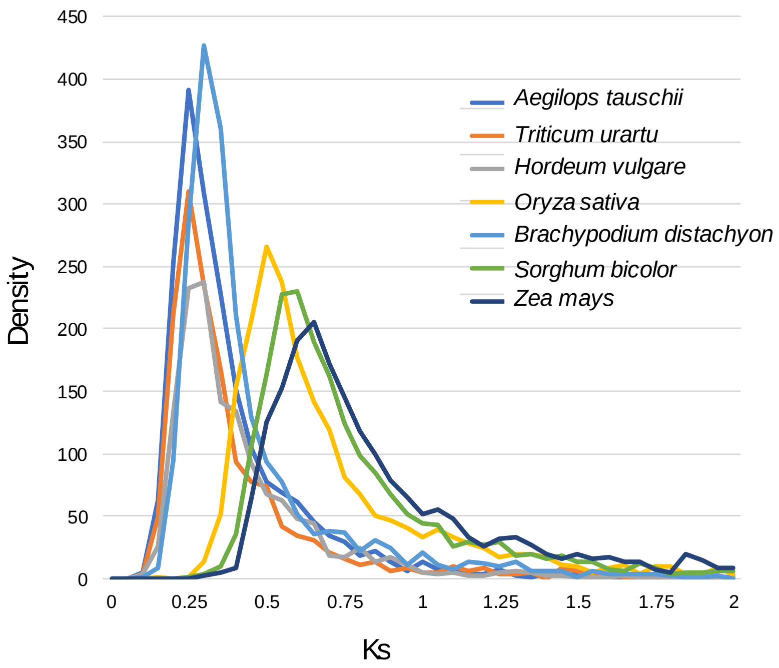

Supplementary Fig. 13. Distributions of synonymous substitution rate (Ks) between *Avena strigosa* S75 and grass species from other subfamilies. Source data are provided as a Source Data file.

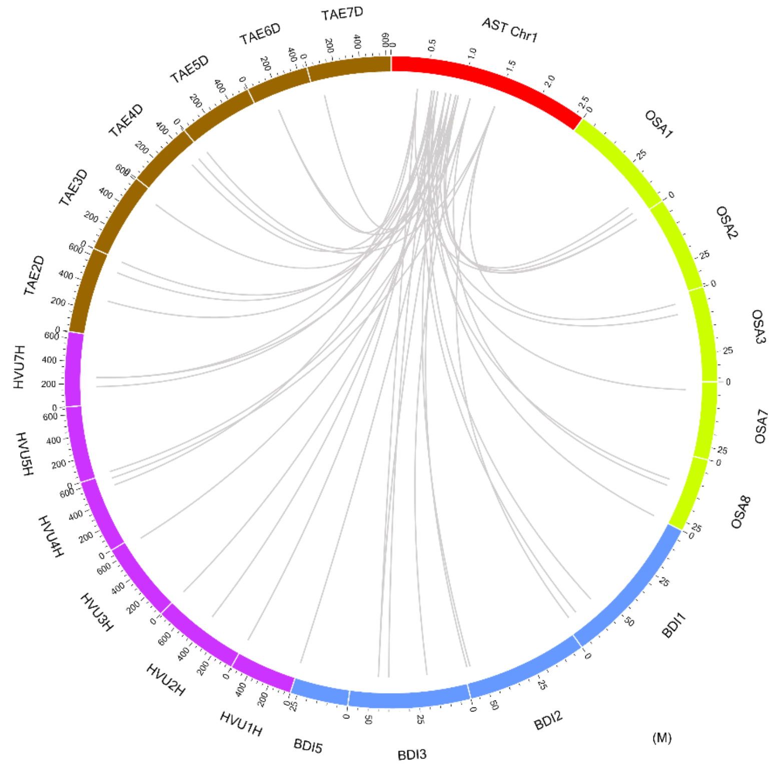

Supplementary Fig. 14. Circos plot showing the locations of the closest orthologs of the ten *A. strigosa* genes between the avenacin cluster and the telomere (region shown in red) on the chromosomes of rice (OSA; green), *B. distachyon* (BDI; blue), barley (HVU; purple) and wheat (TAE, DD genome; brown).

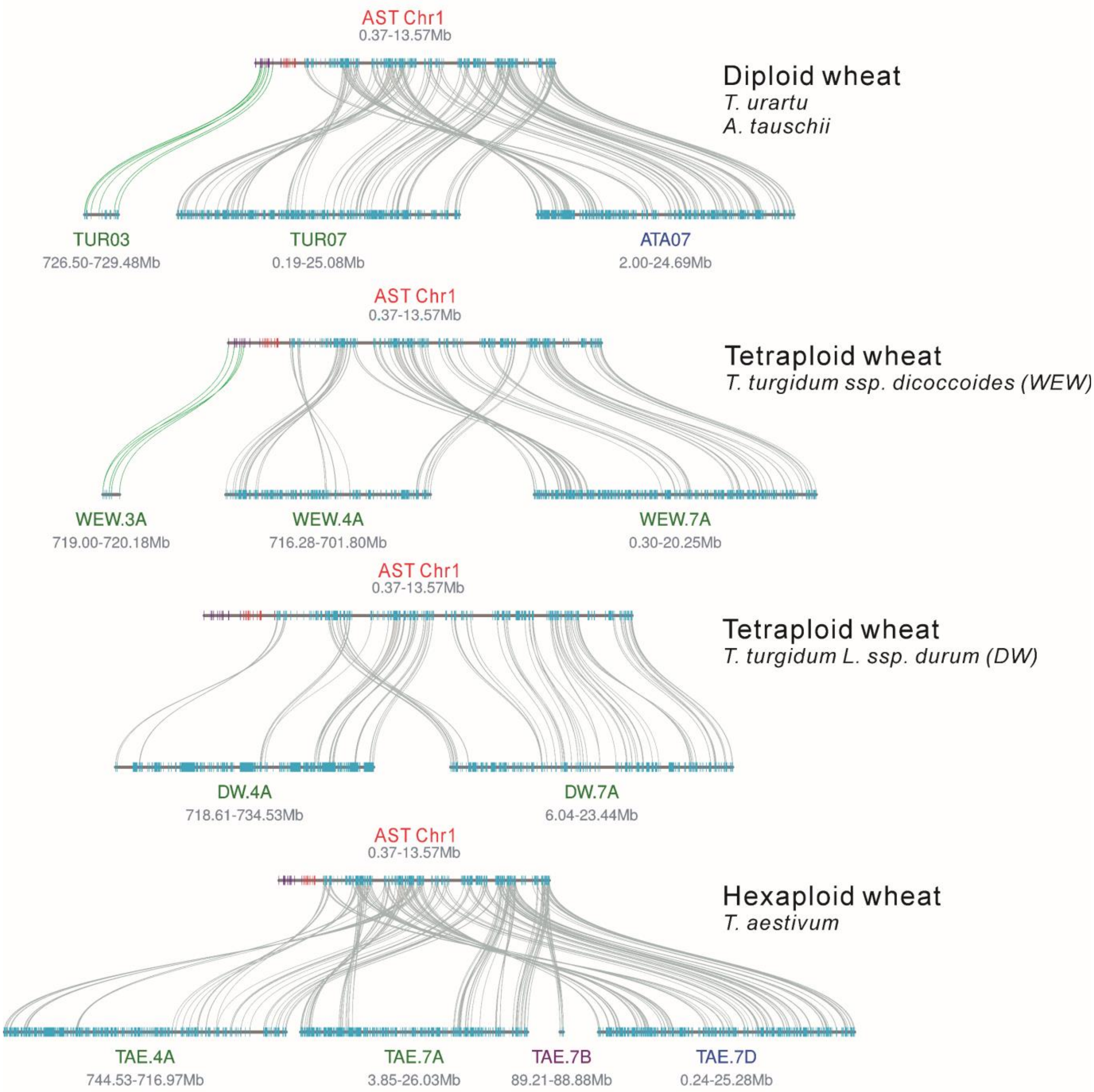

Supplementary Fig. 15. Local synteny of the end of chromosome 1 of *A. strigosa* S75 with diploid, tetraploid and hexaploid wheat. The avenacin pathway genes are shown in red, and the ten genes to the left of these at the end of the chromosome in purple. Other genes are in green. The avenacin pathway genes have no orthologs in diploid, tetraploid or hexaploid wheat. However, the genes in purple have orthologs on chromosome 3 of *T. urartu* and *T. turgidum* ssp. *dicoccoides* (wild emmer wheat) but not in *A. tauschii*, *T. turgidum* spp. *durum* (durum wheat) or hexaploid wheat (*T. aestivum*).

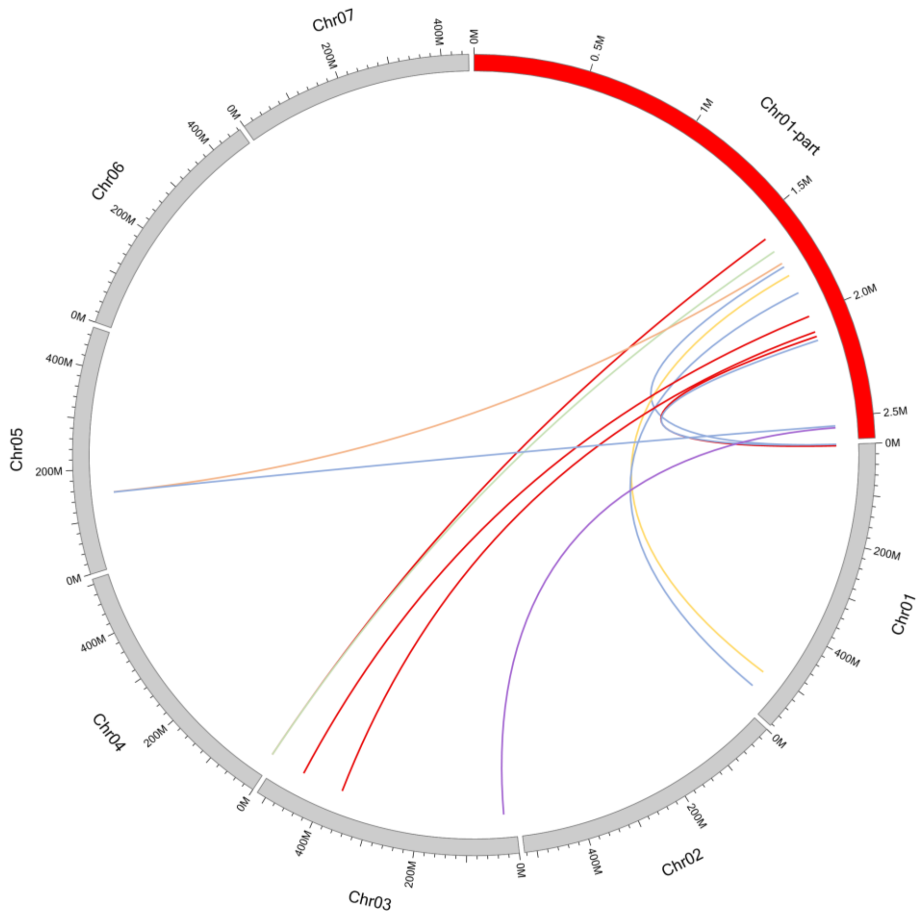

Supplementary Fig. 16. Circos plot showing the locations of the closest orthologs of the avenacin cluster genes on the chromosomes of *A. strigosa* S75.

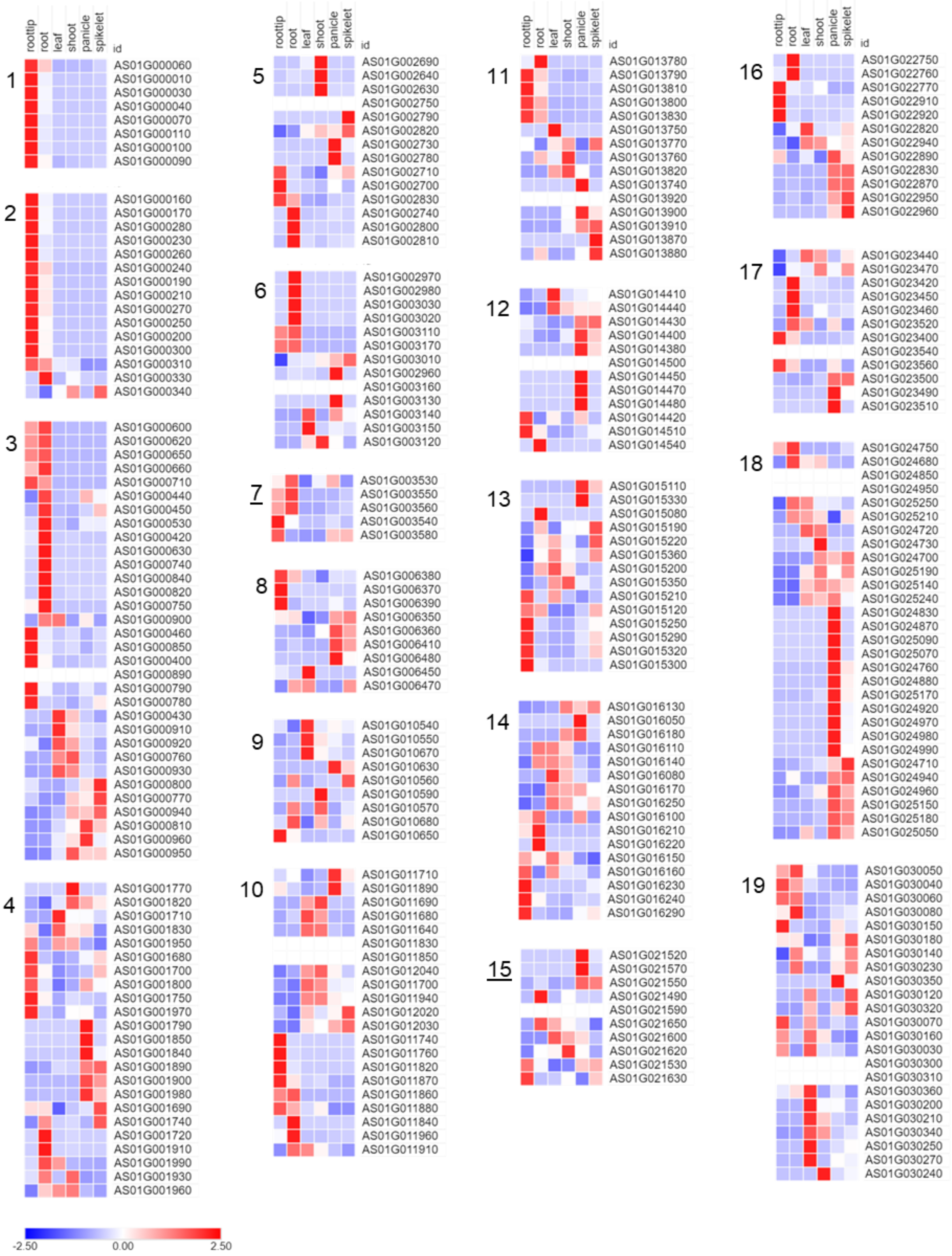

Supplementary Fig. 17. Gene expression heatmap of plantiSMASH-predicted biosynthetic genes clusters on *A. strigosa* Chr.1 100 Mb terminal region. Clusters are numbered according to their position on the chromosome- cluster 1 is nearest to the telomere. Clusters 7 and 15 (underlined) do not include a group of three or more co-expressed genes. Genes within each cluster are ordered by Pearson correlation-based hierarchical clustering. Heatmap colors represent Z-scores derived, per each gene, from DESeq2-normalized RNA-seq expression data.

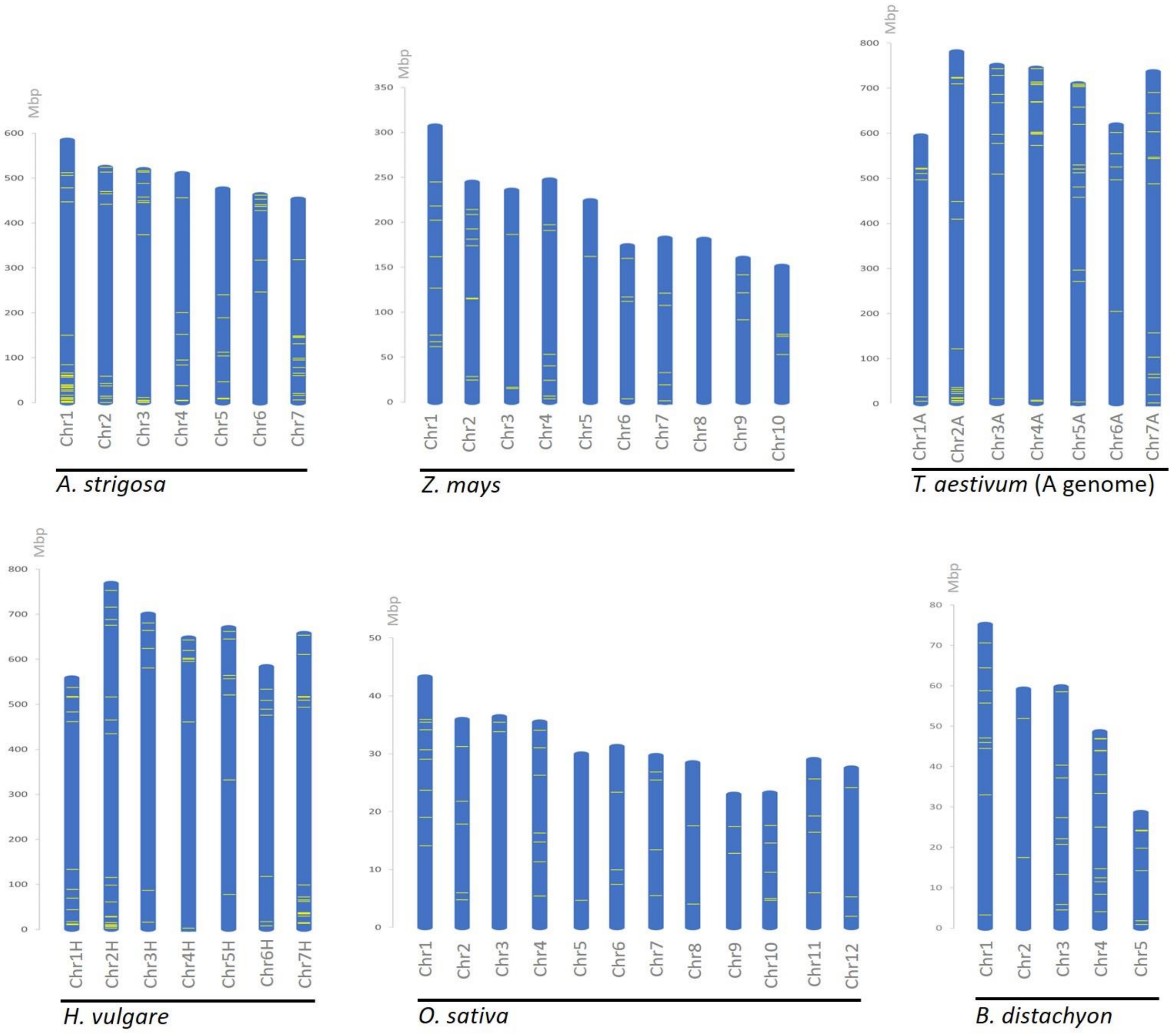

Supplementary Fig. 18. Comparison of plantiSMASH-predicted biosynthetic gene clusters in six grass genomes. Locations of predicted clusters are marked with yellow lines.

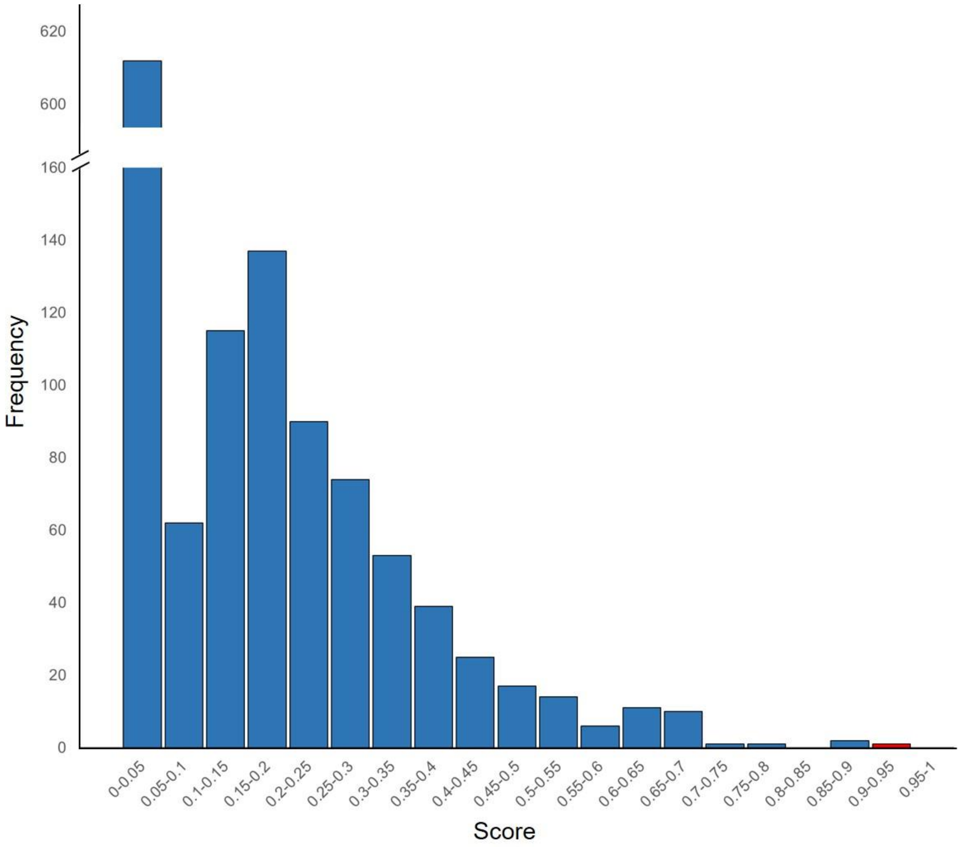

Supplementary Fig. 19. Frequency distribution of normalised cluster density score. Cluster density scores in all chromosomal positions of 100 Mb-sized sliding windows, across the six grass genomes analysed, plotted on a 20-bin histogram. The *A. strigosa* 100 Mb terminal region of chromosome 1 is represented in the red-colored bin.

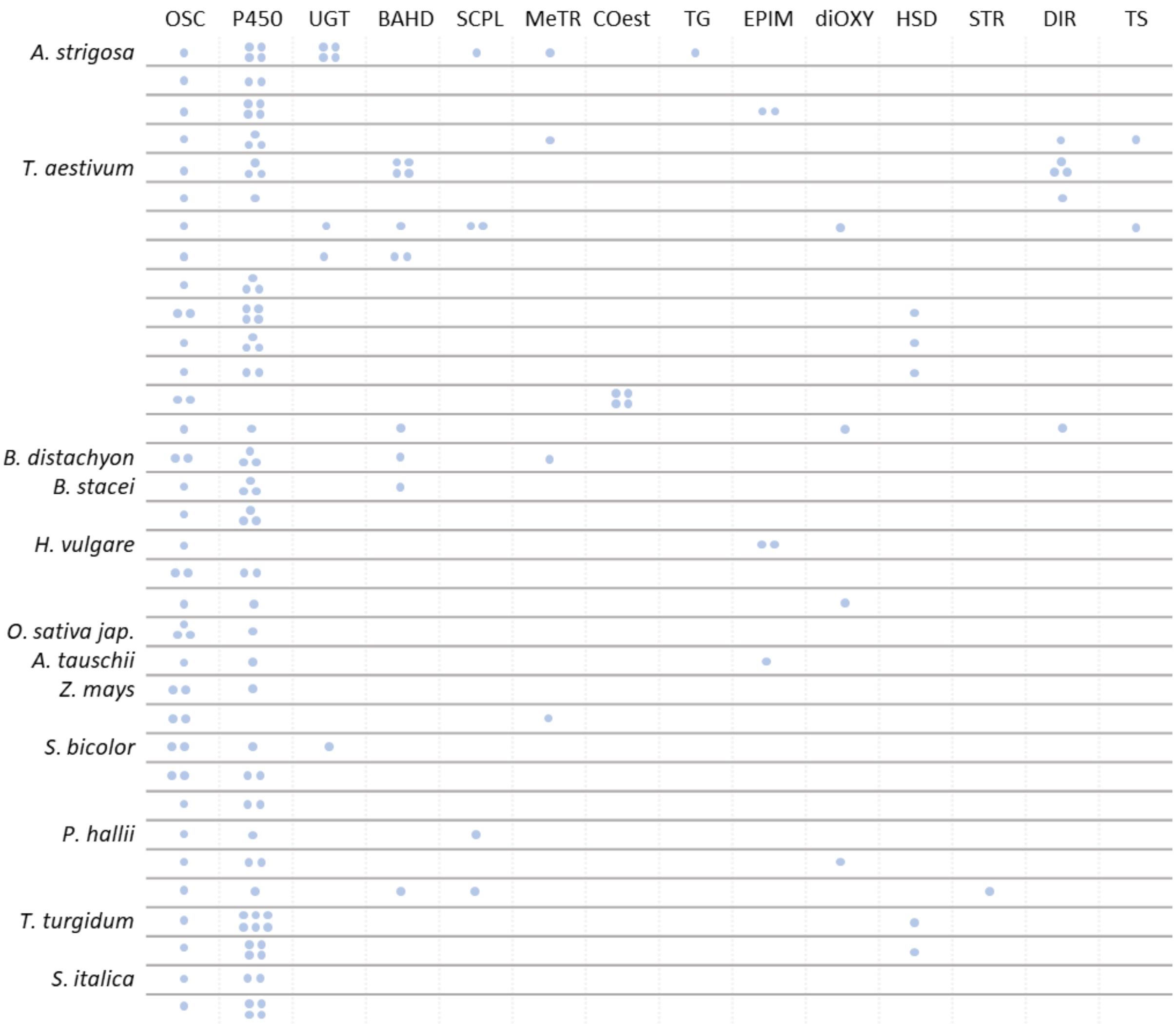

Supplementary Fig. 20. Number of genes and gene super-families in plantiSMASH-predicted triterpene biosynthetic gene clusters in cereal and grass genomes. Each row represents a single predicted biosynthetic gene cluster. The avenacin cluster is shown at the top. The number of dots depicts the number of genes represented for each enzyme superfamily: OSC, oxidosqualene cyclase; P450, cytochrome P450; BAHD, BAHD-type acyltransferase; SCPL, serine carboxy peptidase-like acyltransferase; MeTR, methyltransferase; COest, carboxylesterase; TG, transglucosidase; EPIM, epimerase; diOXY, dioxygenase; HSD, hydroxysteroid dehydrogenase; STR, sterol reductase; DIR, dirigent protein; TS, terpene synthase (non-OSC type).

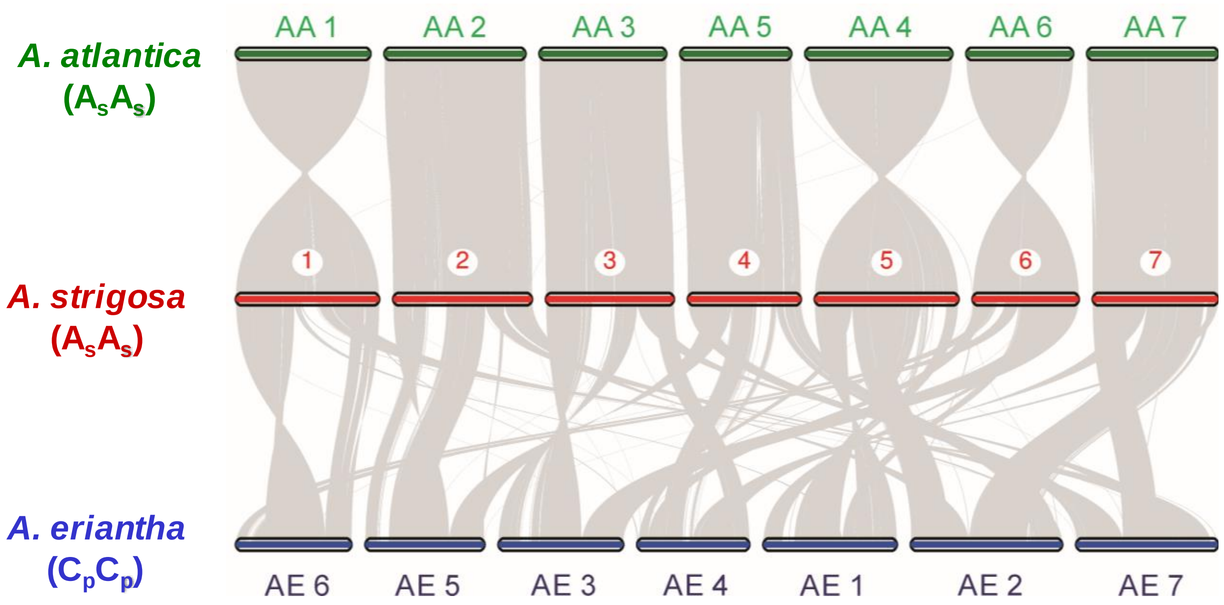

Supplementary Fig. 21. Synteny with *A. strigosa* S75 and *A. atlantica* (both AsAs genome diploids) and with *A. eriantha* (a CpCp genome diploid). The *A. atlantica* and *A. eriantha* genome sequences are from Maughan et al.[3]

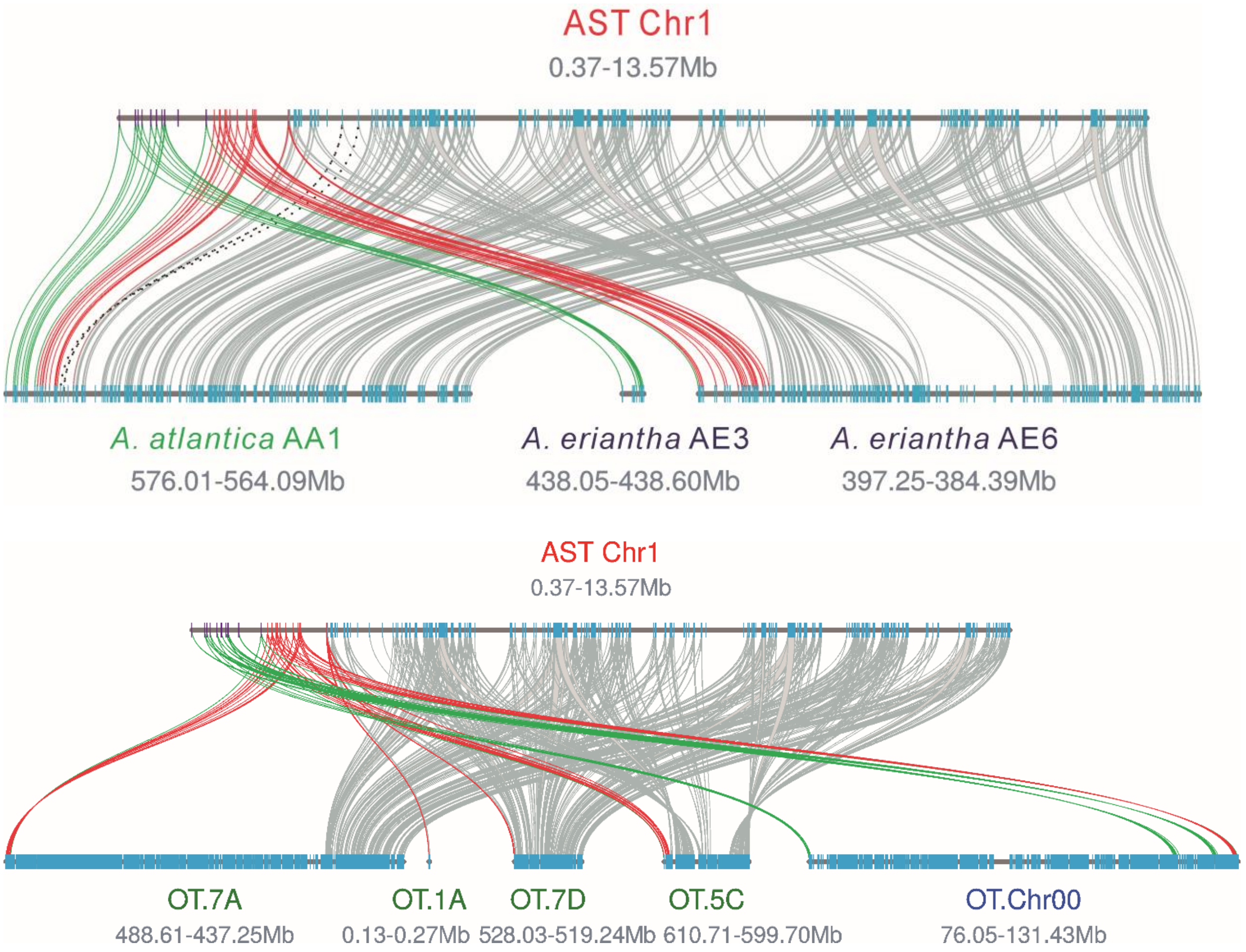

Supplementary Fig. 22. Local synteny of the end of chromosome 1 of *A. strigosa* with the diploid oat species *A. atlantica* and *A. eriantha* (top) and hexaploid oat, *A. sativa* (bottom). The avenacin cluster genes are shown in red in *A. strigosa* and the locations of their closest matches are indicated in the other oat species. The genes between the avenacin cluster and the telomere are shown in purple, and the locations of their closest matches in the other oat species are indicated in green. Other genes are shown in blue, and synteny is indicated by the grey lines.
The A-genome species *A. atlantica* has a very similar region to the *A. strigosa* avenacin cluster in the syntenic position on chromosome 1. The C-genome species *A. eriantha* has a similar region located on chromosome 6, while the subtelomeric genes to the left of the *A. strigosa* avenacin gene cluster are present and conserved on *A. eriantha* chromosome 3 (further detail for these two diploids shown in Supplementary Fig. 22). The A subgenome of *A. sativa* has a conserved region of avenacin cluster on chromosome 7, but the homologs of the last two genes (UGT91 and TG1) are located on chromosome 1. In the C subgenome of *A. sativa* there is also a roughly similar region, but with several rearranged genes. In the D subgenome of *A. sativa*, only the homologs of the last two genes (UGT91 and TG1) of the avenacin cluster were found on chromosome 7. The homologs of the other ten genes were found on Chr00, which is not assembled into the chromosomes. Two conserved regions of the genes in between the avenacin cluster and the telomere were also found on Chr00 (further detail for hexaploid oat shown in Supplementary Fig. 23).

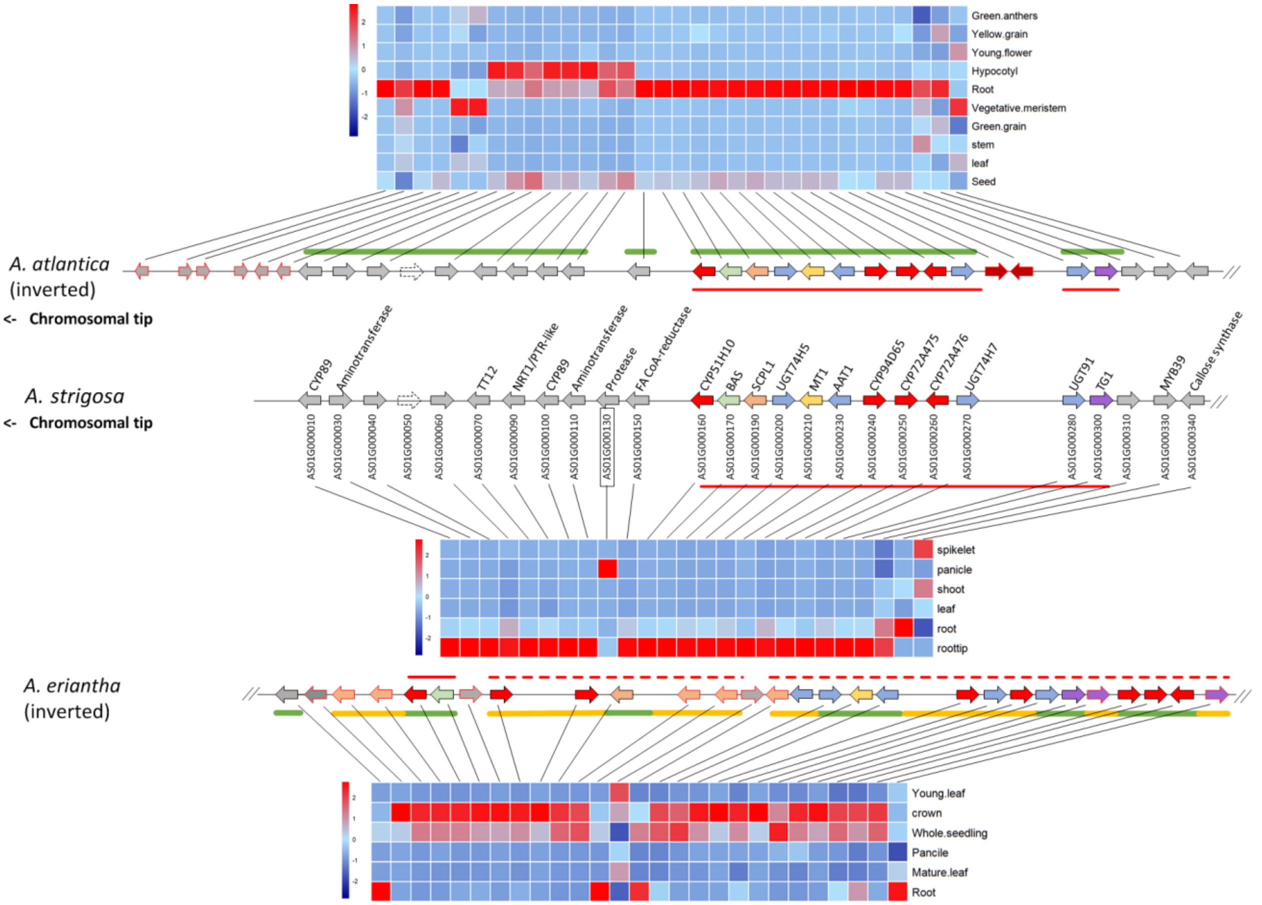

Supplementary Fig. 23. Comparison of the *A. strigosa* avenacin cluster region with related regions in *A. atlantica* and *A. eriantha.* The *A. strigosa* avenacin cluster is indicated by the red line. *A. atlantica* has a very similar region in the syntenic position on chromosome 1, which is also root-expressed. There are two additional *A. atlantica* genes in between the homologs of *UGT74H7* and *UGT91* that are absent from the *A. strigosa* cluster. However, homologs of these two genes are located ~20 genes to the right of the *A. strigosa* cluster. The genes in between the avenacin cluster and the telomere are also conserved in *A. atlantica*. However, *A. atlantica* has an additional six genes (indicated by grey arrows with red outlines) beyond these at the end of the chromosome. No corresponding syntenic block of six genes was found anywhere in the *A. strigosa* genome. In *A. eriantha* there is a loosely similar region but with numerous additional genes. Although most of these *A. eriantha* genes are expressed at moderate level in the roots, they are primarily expressed in whole seedlings and in the crowns. Source data are provided as a Source Data file.

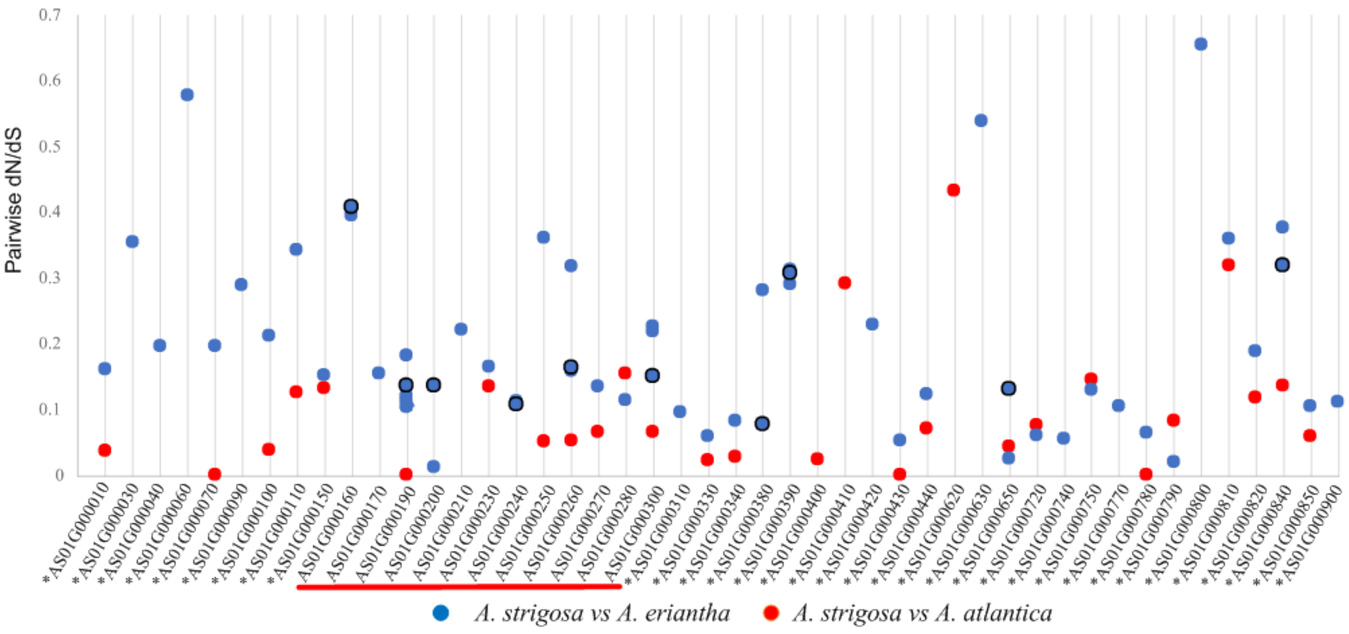

Supplementary Fig. 24. Pairwise dN/dS analysis for the avenacin gene cluster region in the sequenced diploid oat species. The genes of the avenacin gene cluster are indicated by the red line, and the flanking genes by asterisks. Notably, some of the genes in the avenacin cluster in *A. eriantha* are duplicated; the best-matched homologous genes are marked with a black circle. For each dataset, the likelihood ratio test (LRT) was conducted to assess the statistical significance. The p value was determined by comparison to $\chi 2$ with one degree of freedom. Only dN/dS ratios with $p < 0.05$ were considered as significant and plotted. Source data are provided as a Source Data file.

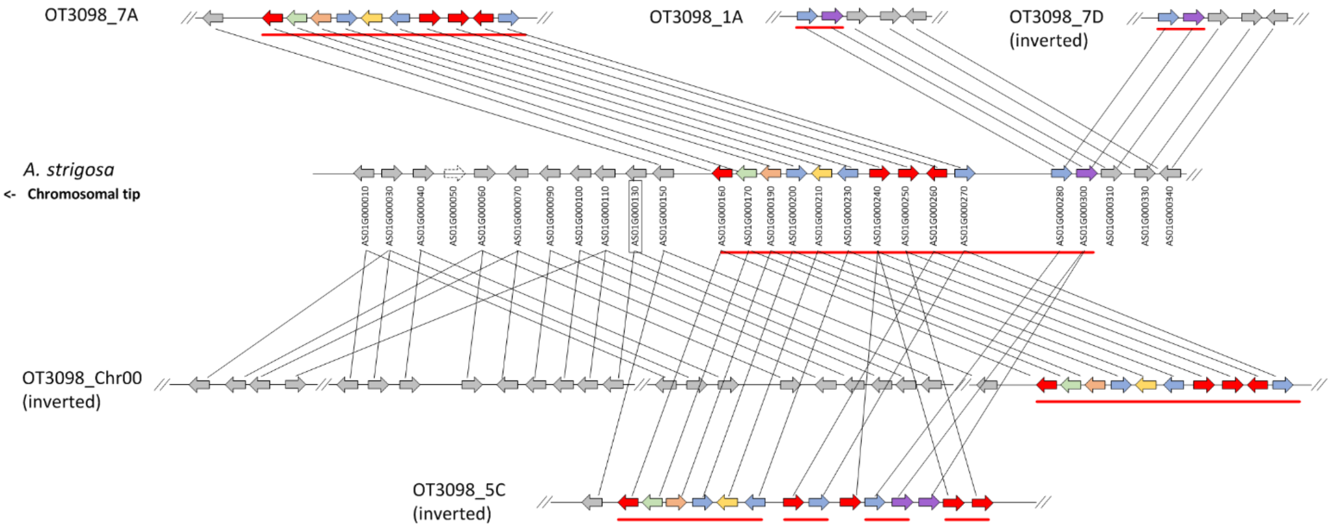

Supplementary Fig. 25. Comparison of the *A. strigosa* avenacin cluster region with related regions in the hexaploid *A. sativa* genome. The A subgenome of *A. sativa* has a conserved region of avenacin cluster on chromosome 7, but the homologs of the last two genes (UGT91 and TG1) are located on chromosome 1. In the C subgenome of *A. sativa* there is also a roughly similar region, but with several rearranged genes. In the D subgenome of *A. sativa*, only the homologs of the last two genes (UGT91 and TG1) of the avenacin cluster were found on chromosome 7. The homologs of the other ten genes were found on Chr00, which is not assembled into the chromosomes. Two conserved regions of the genes in between the avenacin cluster and the telomere were also found on Chr00.

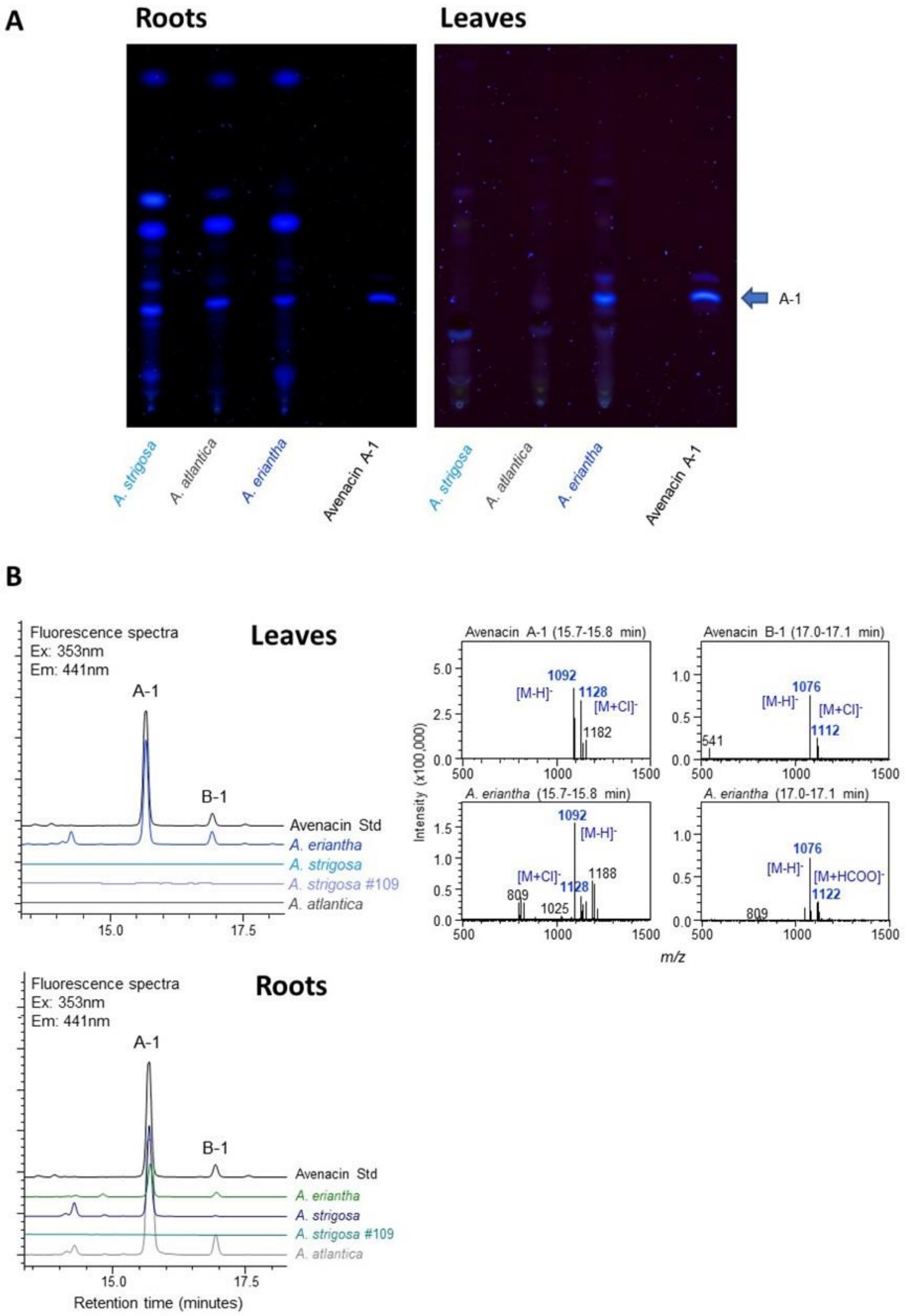

Supplementary Fig. 26. Analysis of avenacins in extracts from the roots and leaves of diploid oat accessions. TLC analysis (A) and LC-MS analysis (B) of extracts from the roots and leaves of *A. strigosa*, *A. atlantica* and *A. eriantha* reveal presence of avenacins in the leaves of *A. eriantha.*

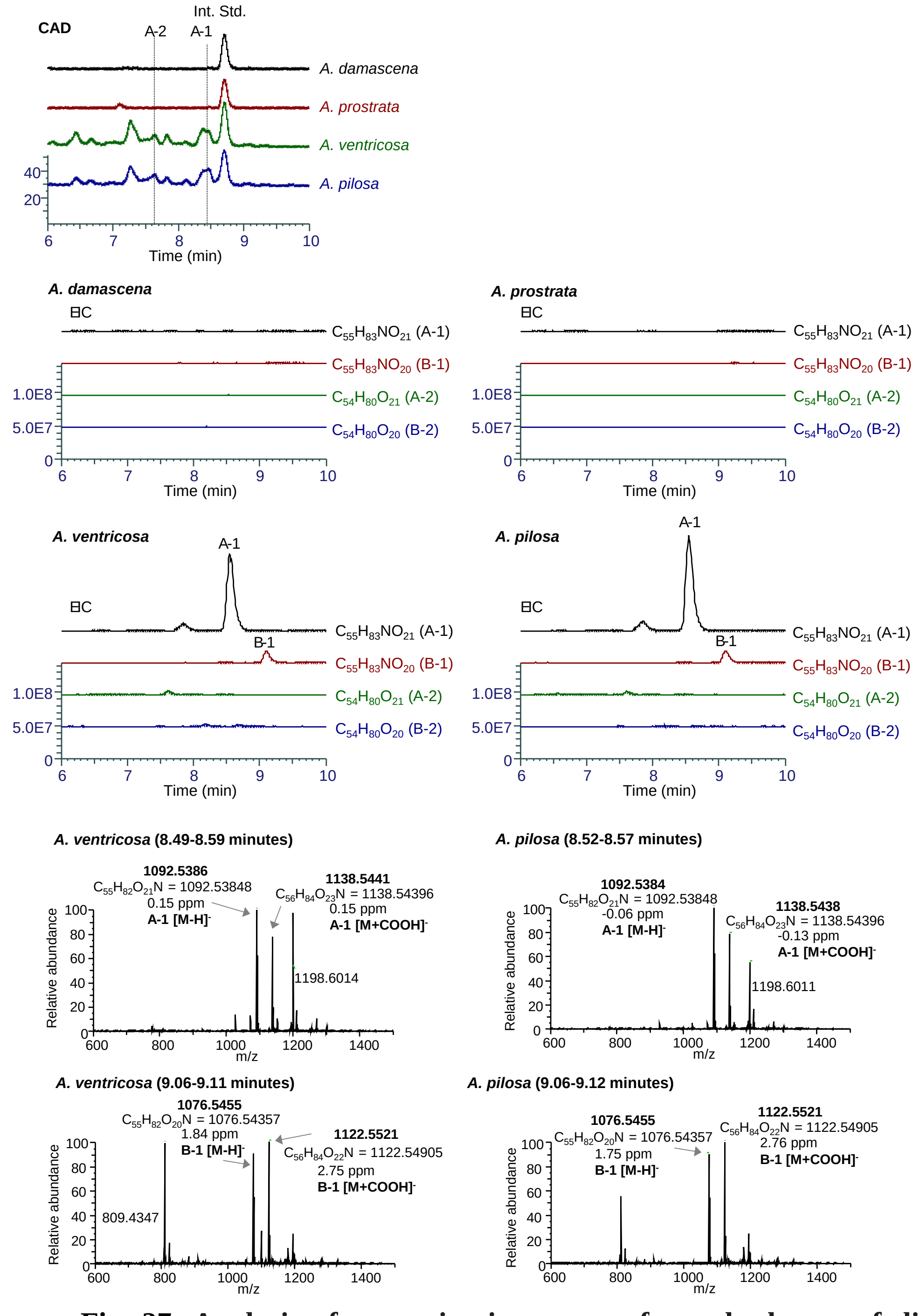

Supplementary Fig. 27. Analysis of avenacins in extracts from the leaves of diploid oat accessions by high resolution LC-MS. LC-CAD-MS analysis of extracts from the leaves of two A genome oat species (*A. damascena* and *A. prostrata*) and two C genome species (*A. ventricosa* and *A. pilosa*) reveal the presence of avenacins in the leaves of the C genome species. Extracted ion chromatograms (EIC) for each avenacin were based on the predicted mass of the [M-H]$^-$ ion with an error of 5 ppm. Int. Std, Internal standard (digitoxin).

Supplementary Table 1. Summary of Oxford Nanopore Technologies (ONT) assembly metrics.

	Contig (Canu+Smartdenovo)	Contig (Canu+Smartdenovo+Pilon3x)	Contig (corrected with Bionano)	Scaffold (Canu+Smartdenovo+Pilon3x+Bionano)
**Total length**	3,440,708,650	3,507,464,261	3,530,496,489	3,530,496,476
**Number of contigs**	1384	1384	1,478	289
**Average contig length**	2,486,061	2,534,295	2,373,115	12,216,250.7
**Maximum contig length**	28,586,057	29,027,854	28,939,783	272,903,458
**N50**	4,680,507	4,770,050	4,651,426	73,363,070
**N60**	3,748,623	3,824,069	3,755,767	54,680,239
**N70**	2,897,678	2,960,716	2,850,768	40,874,734
**N80**	2,059,184	2,097,259	2,060,138	31,630,497
**N90**	1,313,441	1,336,001	1,297,496	16,293,074

Supplementary Table 2. Characterization of the seven *A. strigosa* pseudomolecules.

Chromosome	Pseudomolecule length (bp)	No. of scaffolds	No. of high confidence genes
Chr01	583,880,110	35	6,006
Chr02	523,817,500	48	6,197
Chr03	518,309,500	36	5,554
Chr04	509,801,500	40	4,898
Chr05	475,748,926	44	6,565
Chr06	462,827,500	28	4,738
Chr07	452,504,500	26	5,855
Total	3,526,889,362	257	39,813

Supplementary Table 3. Characteristics of high-confidence (HC) and low-confidence (LC) protein-coding genes annotated in the *A. strigosa* S75 genome.

Metric	HC	LC
Total genes (no.)	39,885	36,816
Single-exon genes (no.)	11,675	13,444
Multi-exon genes (no.)	28,210	23,372
Mean gene length (bp)	3,721	2,518
Mean CDS length (bp)	1,267	741
Max CDS length (bp)	16,176	8,688
Min CDS length (bp)	150	150
Mean exons per genes (no.)	4.5	2.6

Supplementary Table 4. Functional annotation of HC genes in the *A. strigosa* S75 genome.

	**Number**	**Percent (%)**
**InterPro**	29,863	74.9
**GO**	21,781	54.6
**Pfam**	28,677	71.9
**Homologous gene***	33,928	85.1
**Annotated**	34,928	87.6
**Unannotated**	4,957	12.4
**Total**	39,885	100

*****Annotations are based on comparison with barley and wild einkorn (AA genome) wheat.

Supplementary Table 5. Completeness of the assembled scaffolds and the high-confidence (HC) gene annotation as assessed by BUSCO.

BUSCOs	Assembled scaffolds	HC genes
Complete BUSCOs (C)	1,339 (97.4%)	1,314 (95.5%)
Complete and single-copy BUSCOs (S)	1,291 (93.9%)	1,268 (92.2%)
Complete and duplicated BUSCOs (D)	48 (3.5%)	46 (3.3%)
Fragmented BUSCOs (F)	10 (0.7%)	35 (2.5%)
Missing BUSCOs (M)	26 (1.9)	26 (2.0%)
Total BUSCO groups searched	1,375	1,375

Supplementary Table 6. Repetitive sequences in the *A. strigosa* S75 genome.

	Length occupied (bp)	% of sequences
**Class I elements (Retroelements)**	**2,390,363,061**	**67.71**
**LTR Retrotransposon**	**2,346,868,675**	**66.47**
LTR/Copia	614,003,388	17.39
LTR/Gypsy	1,465,380,999	41.51
unclassified LTR	267,484,288	7.58
**non-LTR Retrotransposon**	**43,494,386**	**1.23**
**LINE**	**39,620,971**	**1.12**
LINE/L1	36,128,796	1.02
LINE/Penelope	2,570,890	0.07
LINE/R1	821,886	0.02
LINE/RTE-BovB	9,999	0.00
**SINE**	**3,873,415**	**0.11**
SINE/L1	3,349,932	0.09
SINE/tRNA	412,533	0.01
unclassified SINE	110,950	0.00
**Class II elements (DNA Transposons)**	**219,943,352**	**6.23**
**DNA Transposon**	**219,943,352**	**6.23**
DNA/En-Spm	173,968,889	4.93
DNA/Tourist	3,829,005	0.11
DNA/Harbinger	5,865,386	0.17
DNA/hAT-Ac	777,259	0.02
DNA/hAT-Tip100	942,426	0.03
DNA/MuDR	18,051,295	0.51
DNA/TcMar-Stowaway	10,360,082	0.29
unclassified DNA Transposon	6,149,010	0.17
**Unknown repeats**	**254,548,795**	**7.21**
**Total transposable elements**	**2,864,878,992**	**81.15**
**Satellites**	**12,214,883**	**0.35**
**Simple repeats**	**2,379,586**	**0.07**
**Low_complexity**	**0**	**0.00**
**Total bases masked**	**2,873,875,928**	**81.40**

Supplementary Table 7. Predicted/known functions of the genes on the three scaffolds shown in Fig. 2A.

A. strigosa protein ID （Chr01）	*A. strigosa* protein ID （Scaffold）	Closest match in NCBI database	% amino acid sequence identity	E value	Species	Protein sequence ID
AS01G000010	AS02_290_00013	CYP89N1	100	0	*Avena strigosa*	AYG99329.1
AS01G000030	AS02_290_00011	Predicted protein	89	0	*Hordeum vulgare subsp. vulgare*	BAJ95346.1
AS01G000040	AS02_290_00010	5-Pentadecatrienyl resorcinol O-methyltransferase	77	0	*Triticum urartu*	EMS35720.1
AS01G000060	AS02_290_00008	Unnamed protein product	55	1.00E-43	*Triticum turgidum subsp. durum*	VAH46091.1
AS01G000070	AS02_290_00007	Protein TRANSPARENT TESTA 12	91	0	*Triticum urartu*	EMS59510.1
AS01G000090	AS02_290_00005	Unnamed protein product	65	0	*Triticum aestivum*	SPT21097.1
AS01G000100	AS02_290_00004	Cytochrome P450 89A2	80	0	*Triticum urartu*	EMS68396.1
AS01G000110	AS02_290_00003	Serine palmitoyltransferase 2	92	0	*Triticum urartu*	EMS46639.1
AS01G000130	AS02_290_00001	Subtilisin-like protease SBT5.3	88	0	*Brachypodium distachyon*	XP_003571633.1
AS01G000150	AS02_289_00002	Fatty acyl-CoA reductase 2-like	75	0	*Aegilops tauschii subsp. tauschii*	XP_020148192.1
**The avenacin biosynthetic gene cluster:**						
**AS01G000160**	**AS02_289_00003**	**Cytochrome P450 (CYP51H10/SAD2)**				**ABG88961.1**
**AS01G000170**	**AS02_289_00004**	**Triterpene synthase (bAS1/SAD1)**				**AAT38897.1**
**AS01G000190**	**AS02_289_00006**	**Serine carboxypeptidase-like acyltransferase (SCPL1/SAD7)**				**ACT21078.1**
**AS01G000200**	**AS02_289_00007**	**UDP-glycosyltransferase (UGT74H5/SAD10)**				**ACD03250.1**
**AS01G000210**	**AS02_289_00008**	**Anthranilate N-methyltransferase (MT1/SAD9)**				**AFU52936.1**
**AS01G000230**	**AS02_289_00010**	**UDP glycosyltransferase (UGT99D1/AAT1)**				**AZQ26921.1**
**AS01G000240**	**AS02_289_00011**	**Cytochrome P450 (CYP94D65)***				**XP_004970286.1**
**AS01G000250**	**AS02_289_00012**	**Cytochrome P450 (CYP72A475/SAD6)**				**AYG99326.1**
**AS01G000260**	**AS02_289_00013**	**Cytochrome P450 (CYP72A476)***				**AYG99327.1**
**AS01G000270**	**AS02_289_00014**	**UDP glycosyltransferase (UGT74H7)**				**ACD03246.1**
**AS01G000280**	**AS02_026_00105**	**UDP glycosyltransferase (UGT91)**				**QHG10987.1**
**AS01G000300**	**AS02_026_00103**	**Glucosyl hydrolase (TG1/SAD3)**				**QHG10988.1**
AS01G000310	AS02_026_00102	Binding partner of ACD11 1-like	91	3.00E-172	*Aegilops tauschii* ssp. *tauschii*	XP_020151469.1

AS01G000330	AS02_026_00100	Transcription factor MYB39-like	82	0	*Aegilops tauschii* ssp. *tauschii*	XP_020179049.1
AS01G000340	AS02_026_00099	Callose synthase 10	94	0	*Brachypodium distachyon*	XP_010228114.1
AS01G000350	AS02_026_00098	Alanyl-tRNA synthetase	62	0	*Triticum urartu*	EMS67609.1
AS01G000360	AS02_026_00097	None				

*Functionally validated in this study.

Supplementary Table 8. ^{13}C & ^{1}H δ assignments for 23β-hydroxy-β-amyrin

Carbon numbering scheme and selected COSY and HMBC

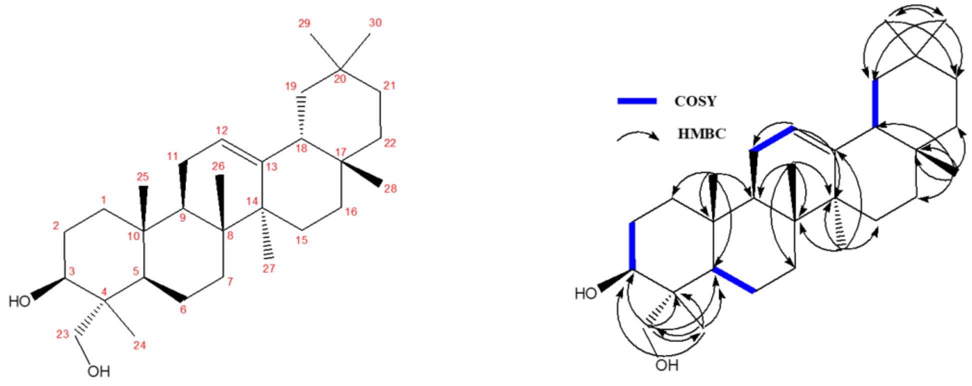

Carbon #	^{13}C δ (150MHz)	^{1}H δ (60 MHz)	Carbon #	^{13}C δ (150 MHz)	^{1}H δ (600 MHz)
**13**	145.17	/	**29**	33.34	0.87 (3H, s)
**12**	121.64	5.18 (1H, t, *J*= 3.5)	**17**	32.49	/
**3**	77.01	3.64 (1H, t, *J*= 7.5)	**7**	32.44	1.50 (1H, m)<br>1.32 (1H, m)
**24**	72.29	3.74 (1H, d, *J*= 10.3)<br>3.45 (1H, d *J*= 10.5)	**20**	31.09	/
**5**	49.82	0.87 (1H, m)	**28**	28.40	0.83 (3H, s)
**9**	47.62	1.58 (1H, m)	**2**	26.92	1.99 (1H, m)<br>0.81 (1H, m)
**18**	47.21	1.95 (1H, m)	**16**	26.83	1.62 (2H, m)
**19**	46.82	1.66 (1H, m)<br>1.01 (1H, m)	**15**	26.14	1.76 (1H, m)<br>0.96 (1H, m)
**4**	41.83	/	**27**	26.01	1.13 (3H, s)
**14**	41.74	/	**30**	23.69	0.87 (3H, s)
**8**	39.79	/	**11**	23.51	1.87 (2H, m)
**1**	38.25	1.64 (1H, m)<br>0.98 (1H, m)	**6**	18.55	1.46 (1H, m)<br>1.37 (1H, m)
**22**	37.13	1.42 (1H, m)<br>1.22 (1H, m)	**26**	16.82	0.97 (3H, s)
**10**	36.84	/	**25**	15.87	0.99 (3H, s)
**21**	34.73	1.32 (1H, m)<br>1.10 (1H, m)	**24**	11.36	0.91 (3H, s)

^{13}C & ^{1}H δ assignments. $CDCl_3$ [referenced to TMS]. Coupling constants are reported as observed and not corrected for second order effects. Assignments were made via a combination of ^{1}H, ^{13}C, DEPT-135, DEPT-edited HSQC, HMBC and 2D NOESY experiments. Where signals overlap ^{1}H δ is reported as the centre of the respective HSQC crosspeak. Multiplicities are described as, s = singlet, d = doublet, dd = doublet of doublets, dt = doublet of triplets, t = triplet, q = quartet, quint = quintet, tquin = triplet of quintets, m = multiplet, br = broad, appt = apparent

This structure is consistent with other published literature on characterization of triterpenoids from *Celastrus hypoleucus*[4].

Supplementary Table 9. Cluster density scores in six cereal and grass genomes, based on plantiSMASH-predicted biosynthetic gene clusters

Species	Chr. number	Total no. of genes	Total no. of clusters	Avg. score	Max score
A. strigosa	1	6006	24	0.174	0.923
	2	6197	11	0.065	0.387
	3	5554	12	0.078	0.301
	4	4899	8	0.123	0.305
	5	6565	7	0.071	0.203
	6	4738	7	0.086	0.266
	7	5855	14	0.197	0.513
T. aestivum (A genome)	1A	4344	6	0.066	0.421
	2A	5797	16	0.162	0.712
	3A	5235	9	0.089	0.378
	4A	4861	12	0.094	0.491
	5A	5429	14	0.203	0.881
	6A	4127	5	0.131	0.515
	7A	4573	12	0.207	0.851
H. vulgare	1H	4634	12	0.183	0.665
	2H	6518	17	0.180	0.671
	3H	5760	6	0.052	0.201
	4H	4380	7	0.069	0.372
	5H	6165	7	0.094	0.289
	6H	4544	7	0.119	0.482
	7H	5576	16	0.188	0.675
Z. mays	1	5904	8	0.203	0.355
	2	4738	9	0.242	0.381
	3	4153	3	0.042	0.180
	4	4116	7	0.126	0.337
	5	4481	1	0.034	0.085
	6	3291	4	0.109	0.146
	7	3109	5	0.192	0.323
	8	3561	0	0.000	0.000
	9	2973	3	0.123	0.187
	10	2684	3	0.197	0.251
*O. sativa jap**	1	6269	8	0.128	0.128
	2	5015	5	0.100	0.100
	3	5359	2	0.037	0.037
	4	4155	7	0.168	0.168
	5	3704	1	0.027	0.027
	6	3844	3	0.078	0.078
	7	3554	4	0.113	0.113
	8	3249	2	0.062	0.062
	9	2667	2	0.075	0.075
	10	2628	5	0.190	0.190
	11	3127	4	0.128	0.128
	12	2774	3	0.108	0.108
*B. distachyon**	1	9916	9	0.091	0.091
	2	7631	2	0.026	0.026
	3	7573	9	0.119	0.119
	4	5859	12	0.205	0.205
	5	3324	6	0.181	0.181

*100 Mb-sized window spans entire chromosomes in *O. sativa* and *B. distachyon.*

Supplementary references

1. J. Doležel, J. Bartoš, H. Voglmayr, J. Greilhuber, Nuclear DNA content and genome size of trout and human. *Cytom. Part A* 51, 127-128 (2003).
2. R.G. Latta *et al.*, Comparative linkage mapping of diploid, tetraploid, and hexaploid *Avena* species suggests extensive chromosome rearrangement in ancestral blocks. *Sci. Rep.* **9**, 12298 (2019).
3. P.J. Maughan *et al.*, Genomic insights form the first chromosome-scale assemblies of oat (*Avena* spp.) diploid species. *BMC Biology* **17**, 92 (2019).
4. K.-W. Wang, H.-X. Sun, B. Wu. Y.-J. Pan, Two novel olean triterpenoids from *Celastrus hypoleucus*. *Helvetica* **88**, 990-995 (2005).
