# Supplementary material for: Subtelomeric assembly of a multi-gene pathway for antimicrobial defense compounds in cereals
Source: Nat Commun. 2021 May 7;12:2563. doi: 10.1038/s41467-021-22920-8 (PMC8105312; doi:10.1038/s41467-021-22920-8)
Supplement: Supplementary file 4 — Supplementary Data 3 [file 41467_2021_22920_MOESM4_ESM.docx]

**Supplementary Data 3. *Sad1* and *Sad3* probes used for FISH analysis**

Sad1 domesticated gDNA insert(11.4kb)in the pL1-R2 pICH47811 vector

**CACTTCGTG**cagaagacaattgc**ggag**gtccctatctatatttgagtttatatagatatatcttatttttctgcaaattttgatatattatatgtatttttctgaatttaaataatttagttatgatttttctaagattaatgtggcaaaaaaaagaaaaatagctaagccgtcggcgtaggtctgaaacctacgcgtagacttgacgccgttgctctaccgttagcccaatgggctacgccgacggtcatgtttacgccgacggcggccgtcggcctatttattctacgccgacggcaaaattgggccgacgccccgtcaagctacgccgacggtcccgacattttgccgtcggcgtataaaaagaccgtcggcctatttagttattcccgtagtgtgtcggtgttgacactattatggtaactatcctaaccggtaggttacaattatcagggctttgtcatgcacatttataaatgtgaatcaggttgaactttatttggtgttcccaatcagacgttagtaaacacaatatcagattaacctgggaaatcttcatgtaacttagactaataaaatgcatctgttaccgtgtacaaatactatcactaaccagatccctgcaagacaagatccacggatcatggtgcagcgatttacgagataatctattgactaattatacttgttctagtactatctactgacccttctctggaaga**T**aaccatcgtgtattctgcaccgatggaagtgaatagatctttcttgtattatccctcatgaaggcactcagagcaaacttgagcgaaccttccgcattcatttcttcacatgcggtgtctgatcagtcaaacaacctccagagatttagtaaaaacaatgtctcgggattccgcgattaatttagtcgtcttatggcctcgagtacttgttataataagatgatttgatacttgcagtatctttacaaactgctagctaaattggacagtagctagttttgtcagtctagtacgtactacatagtatttttttctgtatctagtggcactactgaaatctcactttccacgatttcaaataaaaattacctgatctgacatgatcactggctacgccgagattctacaaatatttctataagtagtttgtggattccaatatatatacggattccgtaaagctctcttaccgatggtatgactttagtagtaacaaaatcataggcttcgagtgaagattggctaccaactgtaatgtaagattgttgtccaagataagatactcaagttacagatgcactactctaatactaagagttattgatctatattacggctcccgtaccgtagagatattgattctacgttcaccttcttaaaaggagattcttgtacaatcaaaacaaatgggtctagctaccttggtcaatatgtatttctatcggtatttagttataaaggagaggaatacagaataatttttttaactccatagtacctctattgctttcagtataaagagtttgatgcacggttctctgtactaataaatgttctattgttgattgattcttaaccgcatcctatgcaattttaacctcaaaaaagtttcacggtacaccgacttgccttactagccctactgttttcttgagaaggatgttcaaactttgggcttttgcatctaaaataagacacacatcatttttggtttattattcaacaatgtgtgggaaaagcatacaacaatcaactcgatataccaccttcgcggagggcctcctctttaaatgtctgggagtactacacatatgtaaagatgatgcccacttacaaagaacgaggacaccacttaaaccgggtgtacaaagtactacacatatgtaaagatgaggccatagaacaagcaagagcaccaagatatttagatccactaaaatgcaaccacctcgatgtccataaaaaatgatggtgacgtacaacactcaacaaatatcgataaaaatgatagtgtcctagttgcacatcttctaacatgttggtgtctattatgcacaagtgggcatggaagcaagtaaatattgtgtactatagctactggtgactcgagtgtatctccaagactcgatagcaaacccgaagcctcttcagcttgtccacatatcattgtggaatgttcactacgactcgccacgccaagcataacctggataagccacgtgggatatgagatttcccgcagcttccctctgagtgaggaggcagaactatacgcctcaacacgacgagccaccccctaaggctagtcatagtgggagtaacttgggtagtaacatattcctacatatattgcgaactaagcatttagatgacatgacatgcaattaaatgatgagagagagtcttatgataactagctatgttaccataacatcacacatttctaaaaaaataaatctatattataataaataaggttttgcatgataccacatctatgttattttgcactatgaagatagtaacttagactagtaacatatacatgttactactctaagttactccccacaatgaccagcctaacaccttttgtactgttttgcacatttgcagtttactttttcttaggtgaagagaaaacacaagacataattttaatatttcaacttcattacgtgctggtgcaaataatttttacggtgcaattttcgacatgatttattgtatatttacagaaatttatgctccaaatttgtttggtaccttcagtattagtttctggacattgtacatattatgttgccgtataagctgagctagaaggatcattagtgtaattccatatatatctaaatgtacctgtggaatcacatttgaggaagttccaatgatgccctttttgccctgcacacgcatatataagaaccctttgcccgcagcatagagctagtactagctagtatcccattgcttgttttcctcgcatacactgcccgttgttggtgcgcacc**aatg**tggaggctaacaataggtgagggcggcggtccgtggctgaagtcgaacaatggcttccttggccgccaagtgtgggagtacgacgccgatgccggcacgccggaagagcgtgccgaggttgagagggtgcgtgcggaattcacaaagaacaggttccagaggaaggagtcacaggaccttcttctacgcttgcaggtacatgcgtc**A**tctttcccctacttccatatacacccagtagtatatgttgccactgccgttagctctagctttaggactgagaaaagggctctcagataatccatatctctctttagatggagggtttgcttttatttattattacatactgttcaatccttgctgtgtatatcatcaactgcagtacgcaaaagacaaccctcttccggcgaatattccgacagaagccaagcttgaaaagagtacagaggtcactcacgagactatctacgaatcattgatgcgagctttacatcaatattcctctctacaagcagacgatgggcattggcctggtgattacagtgggattctcttcattatgcctatcattgtaagtattttactattattttatgatacagcaatttggcaattaatatatgcatacgaggtttcttatttcgtaaatactcaagacaatatagcatgtggaatcttataatttctataatgaatatgtaccgtcttgtgtgcgcaatacgtatactatattattccgctatgcatatagtattacataccaatattgatagatgttcaaaccaattatgaagagtttaactacaagatttaatatagtagtttctgttattctagcagcaagttacctccattaggttccggaagttctactcttaccacctatatatatgtattattgcttatactaccttcgtct**T**aaagtttaagacttttttttaaagtcaatttatggaaagtttgaactaacttttataaaactatcaagaactatgatattatatttgccatgtgaaaatatgttttattatgtatcaaagggtatggatttcgtaccgtaaatataatattgttgtctaaaatcttggttgaactttacttagtttgacttttggaaagtatataagccttaaactttaaaatagacgtagtaattcagatgcacttcactgatatcccgacaaaagtacaaaatacatttatggaatgtcaaatttatttgaaaacaacacatttggtttagcttcaatatttcggaaaagaaaatatgaggagtgatttaaataagttcttaaggttttcatgaaaaacaaatctgttatggggactttatgcaaagagaacaagattggctcttagaaatttctttagatatgattaaattaaaatacagtgtttgcactaaaaccacatttggtttgatttgaatatttgaaagagatagaaaatcttgaacatttatttttagggaatataggctttattactaccatcctatgtatcatcgatggtggctcatcacattgatcacaactctgaaaactaagaagtctccaacatttagacaatgatattggtttttcaaatttcagtaacacttacaagaattccgttgattttattctccatccgagaactcatttctcctctcctaataatgatgcacatatatgatgggatcttttctttatgttgcagatattctctttatatgttactagatcacttgacacctttttatctccggaacatcgtcatgagatatgtcgctacatttacaatcaacaggcatgggattaaacctaacacatatttccatatttgttttctatatgtttgtgattttgtgaccaaaataaaaacagtacttaatgcaacatatattgagcagaatgaagatggtggttggggaaaaatggttcttggcccaagtaccatgtttggatcgtgtatgaattatgcaaccttaatgattcttggcgagaagcgaaatggtgatcataaggatgcattggaaaaagggcgttcttggattttatctcatggaactgcaactgcaataccacagtggggaaaaatatggttgtcggtatgttaaataacacaagatatcaatgctcatatatgttctcttctgaactaacgttaaatcaacctactatttgataacatcatagataattggcgtttacgaatggtcaggaaacaatcctattatacctgaattgtggttggttccacattttcttccgattcacccaggtatttctatctagcttgcatatataacaaaattgttgtagaacgcatgcttagaccatcattctgtggaattattctgtgcaatttgttgcttgtggaagcaatttaaccatatatcaaacaaggaatattgaggcatggtacctgaaatagttttttgaaaaatacatgccgaaaaggaaatcaatgtttcaattaggcatgtttgcacgtagattccacaagattctcttgtatatgttttgatcttggagatacatgtatatatttatgtatctttcatattatctcaaaaaaataacatgttactaccccctctatccataataagtgtcggtcacttagtacaaactttatactagcttagtacaaaatggacgactcttattatggattgcagggagtactaaatattatgaagttgaaccttatcattcacaagtaatttattggaaaataatccttcatatgtaggtcgtttttggtgttttacccggttgatatacatgtcaatggcatatctctatggtaagaaatttgttgggcctattagtcctacaatattagctctgcgacaagacctctatagtataccttactgcaacattaattgggacaaggcgcgtgattattgtgcaaaggttagttagttaatcaatcactatatatatgtattcagtttgttagaatatattaatttagcccatgtcactacataatattttcatggattcaagattaagaacatcacgtagaataatgaagtacatcatttcagtacttggtatctcagaaaaaatatagactaagaaagctagtgttcttcaaaaattttatgttgtttcaggaggaccttcattacccacgctcacgggcacaagatcttatatctggttgcctaacgaaaattgtggagccaattttgaattggtggccagcaaacaagctaagagatagagctttaactaacctcatggagcatatccattatgacgacgaatcaaccaaatatgtgggcatttgccctattaacaaggtgaaattattttcaaattgatttgcaccttttactttaataatgacggatgttattccattctaatgttttaacatgtttattgtaattaggcattgaacatgatttgttgttgggtagaaaacccaaattcgcctgaattccaacaacatcttccacgattccatgactatttgtggatggcggaggatggaatgaaggcacaggttggtatagagctcttgtcagatattttgtcaatttaactacgtgccaattcttcacaaccattaacctttttcatgaatatatattttctcaaacaaaatgtgagaatcttttgggttacaaggattttttattttcatctatatctaggttgcattcaataagcatgtttgtgcatgtccgagttctcctgaaccaaactaaaatgcatattctctttagctgc**T**catagtgtatatgaaataaaattatggtaataatatttttactttagttaattctaatgacgaaatagttgatatgcctatatcgtttcgaatatataaatcagaggtagttagaaaaattattggacttacatcaaatgcaaactgtgaatgtataagtaatatgtatacaatcgcaggtatatgatggatgtcatagctgggaactagcgttcataattcatgcctattgttccacggatcttactagcgagtttatcccgactctaaaaaaggcgcacgagttcatgaagaactcacaggtttgttgttctccatattatattattgctcaaattctgaaaagatctaacattaattgtctacccttgaaggttcttttcaaccacccaaatcatgaaagctattatcgccacagatcaaaaggctcatggaccctttcaagtgtagataatggttggtctgtatctgattgtactgcggaagctgttaaggttaacataagaaccatg**C**cttccaattgtacatatataagtacatatgtgaatacatgacgggttaccctgtataagttgaaatgaactattcatgaatatattgaatctacattaatattcattattttttcaggcattgctactattatcaaagatatccgctgaccttgttggcgatccaataaaacaagacaggttgtatgatgccattgattgcatcctatctttcatggtatgagaatctaaattggatcaattaacaaacgtacattactaaacaagggaaactatgcagacccattactaaagaaatgtgagcccacctagctagataattttatctaaaagtattaaattatattttgcacaacatacaaaagttaaatttgttgtacaatgcatattattttctaaaaaaaatgcaaaaataatttggagaaattttataaggtagtccacggtaatttaatccatttctataatgcaaatagagtctcactaatagcag**T**tctccttttctcgttttgaagaatacagatggaacattttctacctacgaatgcaaacggacattcgcttggttagaggttagtgatattcctttaaagttttataacatggtacaattaagatgaaatatcatttttgtattgtatgacttgtccatgagaacaaggtattgggattgaataagaagtcaaaagaaaaccaaatacaacaatgatatattaattgtaattcttatggtcattttgcatttctctttcatacccaagaaattttttctcctgaacaataagtttggataaccctatcccctttaacaaaatatctcttctacgagctaggttctcaacccttctgagagttttcggaacattgtcgtggactatccgtaagacaaaaaacacctacttcataaattatctttacttctatattcaaatattcattttcgcgaactgacttgatatacataataatggtcagatctgttgaatgcacatcatctgtggttgatgctctcatattatttaaaga**A**acgaatccacgatatcgaagagcagagatagataaatgcattgaagaagctgttgtatttattgagaacagtcaaaataaggatggttcatggtaagtgacatgatataaattatgcgttacaataacttttacttttgattaaatttgaaaatttattacttcttgtatctcataggtatggctcatggggtatatgtttcgcatatggatgcatgtttgcagtaagggcgttggttgctacaggaaaaacctacgacaattgtgcttctatcaggaaatcatgcaaatttgtcttatcaaagcaacaaacaacaggtggatggggtgaaga**T**tatctttctagtgacaatggggtaatataacaaactactttacccctataacattttactaatggtaaatcaaatccatcatgattattcatagatttaagtcatacatgatatagtaaacatagaaattgatactagttgagagttttgttgttctataaatatactagttgagagttcagtagttctataagccatgcatggaattacgaaacattataaactcaacgcaagatgatatagttgacaaattttaaaaatatcatatgtcttgttaaaaaaaagatcctagttatattttgagcatgaatatcttcaaatattgtgtatatgtgagaaaagtgatgtttaattgcacaaggtacactaaataaaatggttaatttgtgtatgcccaaaaaagagagatatagaaagagctaaagtaaacttttaattgacacatcttgttcttaacatttattttttatgaaagtctcagtacattcacgatacctagcaatatgaaaattcattgattagacagaagataaaattgccattccacaattattaaatcatatatgttaatttttgcctttttgcttatttttgtcatgataatggatgcataacaccatgttttccaatggtc**A**catctactaatctcagatatcttaatacagcttggtctacaactgttacaccccagttttgatgaccgtgtccacaaatacattataaactactactaatgacctaacacaaaaaaatgacgacaaagacaaatatccaatagaaggatcttttgtactgaacaaatgaagaaaattgtacatatatattgtgtaatatttaatttgttttcctttagtgtactccatccatgaaaatgatattaaatcaatatttgcaatcatgcggtcaaatctgttcttctagatgcgtaagctaaagtcattatatgtatatatatattttcaagaacacataggcatgttgtgttttctaatcacgttttgtacaggaatatattgatagcggtaggcctaatgctgtgaccacctcatgggcaatgttggctttaatttatgctggacaggtttgtcaaatatttttccttgtttgtctagaatatgaattttttattaaaaaggaaaagttctcactattcttgaatagtcgagttatctaacagaataatttatattttgtttttttaataaggttgaacgtgacccagtaccactgtataatgctgcaagacagctaatgaatatgcagctagaaacaggtgacttcccccaacaggtaatatgtttccgtcctacatgttttcaaacaaaaatgcaaagtaatcttaagtttaattgaaactgatcttttgttaatgaaactcaatgtagaccttaagggaacaaccagtagaaataaaatacttgtgaattaataactctggaaagtgtatgcattaatgttggtgtgaatgtggtaaatgttggcattgcgtcataatttttgcatcggtacttacaaagtttaattaacactaatctcttgtcagattcaatgatcattaaaaattaagatataacctccatctagcttcttacttacagtttcaccttggtaataggaacacatgggttgcttcaactcctccttgaacttcaactacgccaactaccgcaatctatacccgattatggctcttggggaacttcgccgtcgacttcttgcgattaagagctga**gctt**tatggaaacaaacatggatgtctaggctgcgaggaataagaacattgctcccacgagcattcatgcgttattttctttgagaaataagttctcttcctaccgatgtcatcatgtaacttttcggaatattttatgtgtaattggaggtgcacatttactagccatgtatggcagttgaggaaagtcccgcggtttaatcgttgtttttcatagccataggggcggttttgggatgataaaaaaggaag**G**cacggatcagcaaagatggaatttaatttacccctccaccacggagagatattctttggattccctcgtttgatatgattcggaaatcatggctagatgcaagcaagcagcaagcaagattgaatataccaggggcagaggggctcggccgatcgatcgggatcgagtctccatagcagtgctgattgcatgcgccgtgggtctatcacctgaacgtgagagtcatcaggaggagtgtattagtggagatggatcgcctattttctcttccagcaggaaacgaaacgtggggcgtc**A**cttccaccagcgacctcttcatccattggagtatcagtgctcaacggtgatatcgacttcgtggatgatgccaacatggcaataggtgcgagcgtgttttctctccacaagcaaagtcagcctaattaaagagaagcctcttctcatccaaatgtcaagatatggtgggatgagagatttgggtccacacaatgcaccaaaactctgacaacaccattctgcctagtatatggcatatccacagcctttgttttaccaaaaacagagccaatacccaatattgataggaaacccctctgatatccgttgggacaccggaaatgtcaactcaaactcacccaagccggaagaggaccacaagtcaaaaatgcaggtctacatatatcctgacgaggggtgaccacggcaatgcgcgcccatggttatgtacttaggtttcgtgggaaagagagcg**cgct**ggatccgaattcggcattgtctt**CACAGAGTG**

Sad3 domesticated gDNA insert (6.4kb) in the pL1-R2 pICH47811 vector

**CACTTCGTG**cagaagacaattgc**ggag**tctagttctaaccagg**C**agacgcgatcagaaaaagaagtaccattgagcaacgttatctaacaacatatactatttttatagagagactggcatctggaaaccaacaacgtgactaaatggttgatgcattgagcaaagttatcaagttcaaaatcg**C**aagacgcgagcaaataaagtccatcatacatatatttttccaataaagaggctgacgtcgggaacgaattcgagccggaaatctaacatctatcacgaaccgatcgagattttgtcacgtttctgacaattctgtggcagtagagagctgtgaaaatggggaaaacttgcatttttgactaaacggtgatgcatcccaaatcaaac**C**agacgagagttaccgatactgtttgcatcgggattctaaccgtattaattcagttcaggtcaaacgagagaggaaattatggaggctgttgcatcggaatcctaacagttttaattcaagatcacttgcatcgggatgctttcaagatttgattcagttcaagtcaaac**C**agacgagagcttggcaggttgtttgtgtcaggattctaacgtcataagcttcgttcagatcaatggaggcgagagacatcaagattgtttgtgtcggtattctaacaacactaattcatcacaagtcaaat**C**agacgtgagctactctcagtttcggtttataagtcacgcgcctatatctaagtcagacaatttaaatattaaaatgtaaaatgtaatagttgttagatttttaaatgatataacttttatgttatataatttattttttcagtggtcaaattgagaatctaggtatacgtgcaggacttataagactggtcatagtgagagtatcatactagtagtatcatgcatataatactagtatgatactagctctatagtgtatggcatcatatgttagtactccctctgtttgggaatactagttcggctattatttccaaatggagggagtatcatagtagtattatatttaatgatttgtaaaatttctagttttacgtgctatgatacagtatttagctatgatattctaattatctctttcttcattaattgttgtgccacatcagatttttggcaacatggtatgcataatattagttatgatactcccactaggactagcctaaactgaaatggagagagtaccgagttgtttgtctcgagattctaagatcattaaaaaaattggtcaggcaagaagatagttaaaacatttatagtttctcagattttcaaaatgcattgttcataattagatctaccccgtttgagtagatctaccccgttttgagtatgttaattaggagggtcgtggtggcgtcaaaaattgcggtagggtcatggtggcgtcgacggaaggcctcatagggtcatggtggcatcgacgaaaggccccatagggtcattgcattgatccctatcctgaagatgggtttgtaaaagatggaagcggtgatctctgtagcaagtgcatatggtgctcgctcggggtctgctggaccggtgcaccggttttgttgatgtgctttggtgaaaggttagggcatacggctttgattctgacctcccccttcatgtgattagtgattttattcgccaaaagggtggcttcaggttgatcattataatatcttataaggcttcgaggaataattaatgaaagcatgttgtgtgcatctatttgatgcataggctggggttacaccctccattctgaaacaaaaatagatcacattataacacccatttggtataggccttgacagttatctaggtggtaggtcacataagatgacgccacatgaaggtttcatattttttggatttttttgaatttagttgtgcatacccttaccctaaacactatgggtacaccctaaaccctaacactatcatatttctaggaatttttggaaaatatttgatgaaatgtcatattgtggacctataaatgtcaccccgggtgcaaagagaagtctagtatatactagacgtgctacttaccaagtttttagtctcatggtgaagtcagtcatggagaagggcatggaagccgaagatctgaggacgactaagggtggttcgagtgctggtgatgatgaggtttcggctaggtgatttgtgacttggagcagtgacaacagcaagtggggggtgacaacacaggagtaattctcggtctaatcttacagggtgaaagcccaaggtctggtcttagttggttgtgcctagcaataaccttgttggaggcattgttttatgagttcagacc**C**tctcctgggtgaacccctgtgatcgatgattagggcgatgacggcgtgtgcactatttccttcttgcatgcgtcgtttttggagacattagatttccggtgttttttttgtggtggttggtgcgctgcaaaaaggagtacatcactataacaggacttttcttcttttttgtaactcttttttttggaccgtgtgtatcgtccacaaggtcgcaaagccatgcgttgtttgcagaagatagatgtaattggtatctccgcgatattaatatatactctttttaaaaaaaaatcctggtcatgtacaggtgtaacaatgagcacatgatgatcatatcagcatcacggtgattaacaaagtgaatgaagttaatagaacccatataagaagaaaaaatgaaggaatgcaaatgcaactaaaaaattgttaatccaaatcgtgatcttatttatttatgagaaggatttgat**C**tcttccaggctagtgggatcttttacggtgtcctttaaacttattattacatggtgaagattcaccacccaaaaaaaaatagacgagctagctacccttcctccaaaaaaaaaaaaatctgagtcaacgatccacacggcaagtgtcatactagtactactagtaaacatttcgggcagtggtgcactccctagctccagtcccgtggcc**aatg**gcactgctgctctgcttgtttctcttcagcctccggcttgccgccctctcggg**T**gacgttgtggtggcggcgctgacccgccgtgacttccccgacggcttcatcttcggcgccggcacgtcgtcctaccaggtattgctagctactatttttcgctcctccatctatcgtgtaagccgaatagaaagtagtaatttgtatgagtcgatcaggtggaaggggcggttgcggaggatggaagaaagcccagcatttgggacaccttcagtcagggaggttactctgccgacaaatctaccggagatgttgcagcagatcagtaccatcattacaaggtgcccctcgatcgtttactcttgataattacactagattgagtttcttcataataactagattgagtttcttcatatatatattaggaggacgtaaagcttatgcatgagatgggtctagacgcctacagattctccatcgcatggccgcggcttatcccagatggaagaggagatgtgaacccaaagggattggagtactacaataacttgatagatgaactcatacgccatggtaatatatatatatatatatataccttcacttgctttaattttgaggattataagcaagccgccgcgccggccttcaacccgggctgatctgatctgatctgactgactgattgattgcaggcatccaacctcatgtcacaatctaccatttcgatctccctcagtcccttcaggatgaatacgacggactgctgagcccgagattcgtgtaattattaagaacttttccatgcatatatcgatcgatgctacctactatctctgcttgttggttggtttctgaatgaattgggatgtgcttgtatatatgcatgcagggatgattacaccatgttcgcgga**A**acgtgtttcaagagcttcggggaccgggtgaagcattgggtgaccctgaacgagcccaacgtggagggtttaggcggcttcgacaccggcaccatgccgccgcgccgctgctcctaccccttcggcgccaactgcaccgccggagactccaccagggagccctacatcgcggcgcaccacctcctgctcgcgcacgccgccgccgtgtccctctacaggagcaagtaccaggaagcacagagggggcaggtaggaatcaccctgctggccgcgtggtacgagcctgccaccgctgcgtcgccggacgacgtggctgccgctgccaggatgaatgacttcgagatcggatggttcatgcatcctttggtgtatggggactaccctccggtgatgaagagccgggtcggcgcgaggctgccggccataaccatggacctctccaagaatttgaccggatcgttcgacttcatcggccttaaccactacctcatgcttaacgcacgccacgacgagcacgccttcaatctcaagcacagggactacgccgccgacacagcaatagcaggtaattaacactaagtacaactagtatcatattagttagtacaaagccatatagctaaatgtatataacacctttcctgcctggattggattggattcagatgcaatgaaagacatccaagaggtacgtacatacggtgaagcaaaggaaactctctctctttcacacaaagtctccttgggccacggtaaatttatatatgtttctctattaatgggtgcagggccacggtaagtacgctccttgggctctcgggagtctactcgaccacatgcgtgtcaactacggaaatcctcctatcatcatccatgaaaacggtacttgctgcaaattaaaggttttttgtttttcttactgtatataagagatgatgagtggactctgcaaaatagtttagggtgaataatacatatgtttacctctgtaaaacaaaatcagcataatttcttgatacttcattcaggacgggctgacttcgtcaaagacccgagcacgatcagaaccgacgactaccctagatcagaggtcctgcaggactacttggaggttctccacatgtccatacggtgagtttgtttacactgcttattttaaaaaatgccagagccacatatatatacccacaaaaatgatctgcaaactattcagttgaaacacttctcttgaagaatcacaaaaggaattctgttgacatcaggtggttctcgatcttgagtagtagtttctgattatgacagagctatacatgttcttgctgcagtaacggatcggacgcgcggggatactttgtatggtcgtttcttgaccttttcgagctcgcaaatggaaacaccctgcgctttggcctgatcggtgtcgacatgaccgtaaaggagaggacgcggtatgttaggaactccgccagatggtactctagcttcctaaacggcggcgagctgcggcaaccggcggcgcccaagaaacaatattacgactctgcgtga**gctt**tgaggcacctaaggattcatgctgcatcgccggtctactccgcctccgccggtggccttggggtcttggaggtgtggtggactatggctctttgtcggcaagaggtttatcgttctctacttaggtcttttttagtgtcttttttggaattctgaggcggcagttatatcttgatgtcagaataaagttatttccaccctatcctcgcacatcggcagatggcgtgtggagttgtgtatcaggcgg**G**tctctttggatcctaccgtttttcgtgttagtttgtgtggtttcagtttagtcccttccaatctatattgttatctttgacaatggttgctgctctggtgcgtttgtctttcgggtccttagcacgatgattttctcgtttgtctactataataagctcttctacgacaagttttggccttctctggggatggacgagcgagaacggctcgcactaatgtttatagtcgtcgatcgctaggtggtccaacaacctatttgtaatatttattacttttatgtttttttataccaccgttgatgaatattaatagatcggtgaaatttttgcaaaacaagtttga**C**accgcgcggttattcataccgctttttttactgctttataatagccattttcacattgggttaataaagttggtttttttcgtgaagctaatactgctattgccttcttgcacgtggctgcagttagttagcttgttttcagctcaactttcagtttctgcgagaagtgggttcttgctttgtacagtgttctccttaaaaagcagaatggaagtcgcgccggcacaaaatcttctgtttttgctgtgtcaaatttcagagtttggtaatcgttgccacactccagacaggaaggatccagccgccgttccggcaacgattagcgacagccacgtgcgagggtcctgcactgcacatggatgcgcgctcgcgtagccgttcccatcgctctcctccacgtcctcaacta**cgct**ggatccgaattcggcattgtctt**CACAGAGTG**

Color legend in the DNA sequences:

**BLUE BOLD UPPERCASE** DraIII restriction sites used to excise the gene sequence from the pL1-R2 pICH47811 vector backbone

**Black bold underlined** 4 bp linkers used to assemble the pL0M gene parts into the pL1-R2 vector backbone

Red lowercase BpiI, BsaI, Esp31, DraIII restriction sites that were removed by introducing a silent mutation during the Golden Gate domestication process

**RED BOLD UPPERCASE** Silent single nucletotide mutations introduced to remove the endogenous BpiI, BsaI, Esp31, DraIII restriction sites present in the genes of interest sequences
