# Supplementary material for: Subtelomeric assembly of a multi-gene pathway for antimicrobial defense compounds in cereals
Source: Nat Commun. 2021 May 7;12:2563. doi: 10.1038/s41467-021-22920-8 (PMC8105312; doi:10.1038/s41467-021-22920-8)
Supplement: Supplementary file 7 — Description of Additional Supplementary Files [file 41467_2021_22920_MOESM7_ESM.docx]

Additional supplementary information

Title: Supplementary Data 1

Description: The most closely related matches to the genes within the region of *A. strigosa* chromosome 1 shown in Fig. 2A in barley, wheat (DD genome), *B. distachyon*, and rice.

Title: Supplementary Data 2

Description: Alignment of scaffolds to sequences available in GenBank.

Title: Supplementary Data 3

Description: *Sad1* and *Sad3* probes used for FISH analysis.

Title: Supplementary Data 4

Description: Golden Gate constructs used for reconstitution of the avenacin A-1 pathway by transient expression in *Nicotiana benthamiana.*

Title: Supplementary Data 5

Description: Pearson correlation output file for co-expression of plantiSMASH-predicted *A. strigosa* biosynthetic gene clusters.
